# The influence of phylogeny and life history on telomere lengths and telomere rate of change among bird species: A meta‐analysis

**DOI:** 10.1002/ece3.7931

**Published:** 2021-09-10

**Authors:** François Criscuolo, F. Stephen Dobson, Quentin Schull

**Affiliations:** ^1^ CNRS Institut Pluridisciplinaire Hubert Curien UMR 7178 University of Strasbourg Strasbourg France; ^2^ Department of Biological Sciences Auburn University Auburn AL USA; ^3^ MARBEC IFREMER IRD CNRS University of Montpellier Sète France

**Keywords:** evolution, life history, MCMCglmm, multivariate analysis, phylogenetic correlation, telomere

## Abstract

Longevity is highly variable among animal species and has coevolved with other life‐history traits, such as body size and rates of reproduction. Telomeres, through their erosion over time, are one of the cell mechanisms that produce senescence at the cell level and might even have an influence on the rate of aging in whole organisms. However, uneroded telomeres are also risk factors of cell immortalization. The associations of telomere lengths, their rate of change, and life‐history traits independent of body size are largely underexplored for birds. To test associations of life‐history traits and telomere dynamics, we conducted a phylogenetic meta‐analysis using studies of 53 species of birds. We restricted analyses to studies that applied the telomere restriction fragment length (TRF) method, and examined relationships between mean telomere length at the chick (*Chick TL*) and adult (*Adult TL*) stages, the mean rate of change in telomere length during life (*TROC*), and life‐history traits. We examined 3 principal components of 12 life‐history variables that represented: *body size* (PC1), the *slow–fast continuum of pace of life* (PC2), and *postfledging parental care* (PC3). Phylogeny had at best a small‐to‐medium influence on *Adult* and *Chick TL* (*r* ^2^ = .190 and .138, respectively), but a substantial influence on *TROC* (*r* ^2^ = .688). Phylogeny strongly influenced life histories: PC1 (*r* ^2^ = .828), PC2 (.838), and PC3 (.613). *Adult TL* and *Chick TL* were poorly associated with the life‐history variables. *TROC*, however, was negatively and moderate‐to‐strongly associated with PC2 (unadjusted *r* = −.340; with phylogenetic correction, *r* = −.490). Independent of body size, long‐lived species with smaller clutches, and slower embryonic rate of growth may exhibit less change in telomere length over their lifetimes. We suggest that telomere lengths may have diverged, even among closely avian‐related species, yet telomere dynamics are strongly linked to the pace of life.

## Graphical abstract

Telomeres influence cell death and perhaps animal longevity. Telomere dynamics and life histories were examined in a phylogenetic comparative study. Rate of loss of telomeres was lowest in species that have long lives and a slow pace of life.

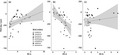

## 1 INTRODUCTION

Senescence affects most living organisms (Gaillard et al., 2020). With advancing age, individual performance in reproduction, physical activities, or cognition diminish, while concomitantly the rate of actuarial senescence (i.e., mortality rate with age) increases (Nussey et al., 2013). The age at which senescence occurs varies among species and individuals (Gaillard et al., 2020), while energy gathered from the environment may be differently allocated to promote self‐maintenance and thus delay aging (Bouwhuis et al., 2010; Jones et al., 2008; Ricklefs, 2008). The view of senescence as an outcome of energy trade‐offs that underlie life‐history trajectories has shaped our understanding of the evolution of animal longevities. Fast‐living species, with rapid growth, early sexual maturity, and high reproduction rates, experience senescence earlier (Jones et al., 2008). However, whether trade‐offs are the only determinants of the rate of senescence has been debated, and environmental and biological constraints may also be influential, particularly in comparisons among species (Cohen et al., 2019). In addition, senescence might differently affect phenotypic traits in relation to their impact on fitness (Hayward et al., 2015), with senescence in reproduction beginning at different points in the lifecycles of different species, compared to senescence in other traits that are directly linked to survival (Gaillard & Lemaître, 2017). Studying aging among species will reveal how longevities covary with other organismal traits, and under which trade‐offs among patterns of growth, reproduction, and life span (Boonekamp et al., 2020; Cohen et al., 2019). Further, studies among species may also reveal underlying cellular and physiological mechanisms of interest that sustained the diversity of animal longevities.

When comparing species, there is a strongly supported pattern of larger body size associated with longer life expectancy (Lindstedt & Calder, 1976, 1981). This association should reflect physiological traits of large species, like lower mass‐specific metabolic rates, which have not been fully characterized (Speakman, 2005a). Metabolism produces damage, as from radical oxygen species (ROS), that are by‐products of aerobic respiration. Radical oxygen species were proposed as the main underlying mechanism of aging, thus linking together the coevolution of body size and life span *via* the higher ROS production of fast‐living, and generally small‐sized species, with higher rates of oxygen consumption (Beckman & Ames, 1998; Buttemer et al., 2010; Delhaye et al., 2016). However, there are caveats to the so‐called free‐radical damage theory of aging (e.g., lower ROS production at high metabolism due to uncoupling of mitochondrial respiration; (Speakman et al., 2015). Cohen et al. (2019) called for additional study of age‐related mechanistic processes that may be integral to the important trade‐offs in play. Thereby, such research might yield a more complete explanation of interspecific variation in life span (Hulbert et al., 2007).

Shortening of telomere length is well known for its role in cell senescence in vitro (Harley et al., 1990). Within species, an increasing number of studies have examined the link between telomere length or its rate of erosion (i.e., telomere rate of change, TROC, expressed as *bp* lost per year, a mostly negative variable) with aging and life span (e.g., a seminal paper by Haussmann et al. (2003)). For instance, in recent comparative analyses, short telomeres have been associated with higher risks of mortality in nonmodel and wild animals (Wilbourn et al., 2018) and TROC was found inversely related to maximum life span in birds and mammals (Sudyka et al., 2015; Tricola et al., 2018; Whittemore et al., 2019). However, the sample size in the latter study was small, and a negative relationship between TROC and life span was lacking in intraspecific studies (Beaulieu et al., 2011; Sudyka et al., 2014). In addition, experimental evidence is lacking for a causal link between telomere length or TROC and life span (Simons, 2015; Young, 2018; but see Varela et al., 2016). Telomere length and TROC need not be highly correlated (Tricola et al., 2018), since the telomere length an individual starts with in life with may have a strong heritable basis (Vedder et al., 2021; but see Becker et al., 2015; Viblanc et al., 2020), and both variables may be differently regulated by up‐stream cell mechanisms (i.e., oxidative stress or telomerase activity; Grasman et al., 2011). As a consequence, they may not display similar associations with life span when comparing species.

Among species, TROC seems more strongly and negatively associated with mean longevity compared to a lack of significant association of mean telomere length and longevity (Dantzer & Fletcher, 2015; Haussmann et al., 2003; Sudyka et al., 2015; Tricola et al., 2018). Those studies also showed that TROC is phylogenetically conserved within bird families, thereby suggesting that mechanisms modulating telomere loss coevolved with life span similarly among closely related species (Tricola et al., 2018). However, Dantzer and Fletcher (2015) extended analyses to associations of TROC and life‐history traits, finding that among 14 bird species, those with a more rapid pace of life also had greater TROC (Dantzer & Fletcher, 2015). The pace of life was calculated using a principal component analysis (PCA) that merged 9 variables on a single PCA axis, including adult body mass. However, body size effects must be removed from pace of life analysis, because the slow–fast continuum in life histories is statistically independent of body size (Gaillard et al., 1989; Read & Harvey, 1989; reviewed by Harvey & Purvis, 1999; Dobson & Oli, 2007). The results of Dantzer and Fletcher (2015), therefore, could not differentiate associations of body size and the slow–fast pace of life with TROC. Body mass was further used as covariate in comparative analysis of interspecific link between TROC and life span among 19 species of birds, and this analysis likely adjusted for variation among species in body size (*via* body mass versus life span and TROC; (Tricola et al., 2018). Body size may also modulate the interspecific variation of telomere length through its interaction with telomere maintenance enzyme, *that is*, telomerase, which appears inoperative in larger rodent species (Gorbunova & Seluanov, 2009), perhaps with an associated risk of cell immortalization (Risques & Promislov, 2018; Seluanov et al., 2007; Tian et al., 2018). In addition, body mass may be a key determinant of survival in animals (Miller et al., 2002), including bird species (e.g., Briga, 2016; Stier et al., 2014; but see Briga et al., 2019), and has a significant association with senescence rate among species (Jones et al., 2008). Thus, one needs to statistically account for differences in body mass among species, to avoid spurious correlative conclusions about relationships between TROC or telomere length and longevity (Speakman, 2005b).

Telomere length at the end of growth seems to predict individual life span (Asghar et al., 2015; Heidinger et al., 2012; Lieshout et al., 2019). Early growth is the period when the greatest amount of telomere loss occurs, due to either rapid cell proliferation or high metabolism during early growth (Monaghan & Ozanne, 2018). Alternatively, longer telomeres early in life may inevitably erode faster (Grasman et al., 2011). Early growth is also the period during which physiological maturation of tissues occurs (Cornell et al., 2017; Ricklefs et al., 1994; Starck & Ricklefs, 1998), a phenomenon influenced by both genetic and environmental factors. From such cell and physiological mechanisms, ontogeny produces the adult phenotype. Rapid growth or stressful early‐life conditions may increase telomere loss and shorten individual life span (Boonekamp et al., 2014; Herborn et al., 2014; Metcalfe & Monaghan, 2003; Tarry‐Adkins et al., 2009). One hypothesis is that growth and longevity are connected *via* the processes that shape telomere maintenance during early life. Still, only a tenuous and indirect relationship presently relates short telomeres to reduced long‐term survival (Quque et al., 2021) and more rapid growth to increased telomere shortening (Vedder et al., 2018), both in wild birds. However, in a related study, interindividual differences in telomere loss were only moderately sensitive to early‐life conditions (i.e., hatching order and resource acquisition ability), termed “canalization of resource allocation” toward telomere maintenance (Vedder et al., 2017). If we hypothesized that this extends to the interspecific level, it suggests selection for reduced erosion of telomere length during growth in long‐lived species. Since those species generally exhibit extended developmental periods, slow growth patterns might have been associated with reduced shortening of telomere length. This hypothesis needs to be tested at the phylogenetic level, as well as within long‐lived avian groups of species like procellariforms, where body size effects can be statistically controlled due to a broad range in body mass (about 30 g to nearly 10 kg (Dobson & Jouventin, 2007). Further, the relatively small procellariform species *Oceanodroma leucorhroa* exhibits an intriguing apparent telomere lengthening over life and maintains telomerase activity in adult somatic cells (Haussmann et al., 2003, 2004, 2005; Tricola et al., 2018).

In the present study, we tested the association of telomere length and TROC with life span, reproduction, and growth patterns among 53 bird species. Our study described the correlative relationships between life‐history traits and telomere variables at the interspecific level. We applied a principal component analysis to typify major elements of life histories, including body size, the pace of life syndrome, and parental care (after, e.g., Bennett & Owens, 2002; Dobson & Jouventin, 2007; Dobson & Oli, 2007). This approach has the advantage of separating body size effects from influences of the pace of life on telomeres and will nicely complement previous study on evolution of telomere length and dynamics in birds (Tricola et al., 2018). We then examined the association of these life‐history indices and telomere variables, both without and with statistical adjustments for phylogenetic influence. Because life span is more closely associated with TROC than with telomere length (i.e., TROC being estimated using between‐individual data; Tricola et al., 2018), we predicted that after statistically controlling for body size, species with a fast pace of life should be characterized by higher TROC (i.e., greater telomere loss *per* time unit) and little pattern of change in adult telomere length (*Adult TL*). In addition, we tested the predicted negative association of body mass and telomere length, previously found in rodents (Seluanov et al., 2007). We expected that *Adult TL* would be negatively associated with body size, and TROC would be positively associated with body size (TROC is a negative value, and large‐bodied species lose less TL). Predictions for *Chick TL* are more difficult to make. Since early growth is when telomeres are shortened the most and long‐lived species have slower developmental rates, other things being equal, fledgling telomere lengths (*Chick TL*), once controlled for body size, might be relatively longer in species with a slow pace of life because telomere shortening is drawn out over a longer period (based on low growth rates and reduced telomere shortening). But *Chick TL* should also be longer in those with a rapid pace of life (based on greater TROC, so long *Chick TL* and short *Adult TL*). Following results of Tricola et al. (2018), we did not expect phylogeny to have a strong influence on *Adult TL* or *Chick TL*, even after controlling for the possible influence of body size (via body mass). On the other hand, phylogeny was found to explain a significant part of interspecific variation of TROC (Tricola et al., 2018). We hypothesized that extracting the influence of body size from the pace of life would lead to a comparable result, if closely related species with identical paces of life have evolved similar TROC independently of their body masses. This expectation seemed reasonable because some orders, like the Procellariiformes, have evolved long life spans along with considerable variation in body masses among species (Dobson & Jouventin, 2007), so they should present different telomere lengths (in relation to body size) but similar TROC (in relation to life span).

## 2 MATERIAL AND METHODS

Our meta‐analysis followed the recommendations of the Preferred Reporting Items for Systematic Reviews and Meta‐Analysis (PRISMA) statement (Liberati et al., 2009).

### 2.1 Data collection

Complete data and references list are presented in the Electronic Supplementary Material (ESM) 1.

We first identified published articles in peer‐reviewed journals that reported telomere length or rate of telomere erosion in birds. We (a) screened our own reference lists, (b) backward searched references sections of selected articles from the list, and (c) collected publications using Google Scholar with the following terms: “telomere” OR “telomere shortening” OR “telomere erosion” AND “birds” OR “avian.” For each retrieved paper, methods were individually appraised by one author (FC) to restrict the selection to studies using the telomere restriction fragment (TRF) method to assess telomere length or erosion. Our meta‐analysis therefore only relies on published telomere length data expressed in absolute length (kilobases, kb). Using absolute values of telomere length (TL) allowed us to do interspecific comparisons of telomere length relationships with other life‐history traits, based on the calculation for each species of a *mean adult telomere length* (hereafter *Adult TL*), *mean chick telomere length* (hereafter *Chick TL*), and *mean telomere length rate of change* over the lifecycle (hereafter *TROC*), allowing interspecific comparisons. We primarily used studies that sampled erythrocytes, with one exception of a study that used tissue attached to feathers. Data extraction is detailed in ESM 2.

Data on life‐history traits were compiled for species for which TRF measurements of *Adult TL* were available. ESM 1 references the principal sources used to collect TRF values and 12 life‐history variables for each species. We included publications in peer‐reviewed journals, online peer‐reviewed databases, and books. The protocol for collecting life‐history data is described in ESM 2. In particular, we examined (a) species body mass and size, (b) longevity (age at maturity and life span), (c) reproductive patterns (e.g., using egg or clutch sizes), (d) growth patterns (pre‐ and posthatching growth rates), and (e) postfledging parental care.

We adjusted statistical analyses for sample sizes using the standard procedure for meta‐analyses (Liberati et al., 2009). We could not control for the mean age at which telomeres were measured in adult individuals of each species. However, age is likely to be related to life‐history variables included in our analysis (e.g., body size, longevity) and likely to scale with body mass. As such, risks of multicollinearity of variables were high. Thus, we used principal component analyses as a variable reduction method (see details below). We conducted a separate analysis of Chick TL to check for specific relationships between life‐history traits and telomere length early in the species' lifecycles. In addition, we analyzed associations of TROC and life‐history traits. As in previous studies, TROC was estimated as the slope of the linear regression between TL and age over life (i.e., including chick and adults life stages; Haussmann et al., 2003; Tricola et al., 2018).

### 2.2 Phylogenetic data

**FIGURE 1:**
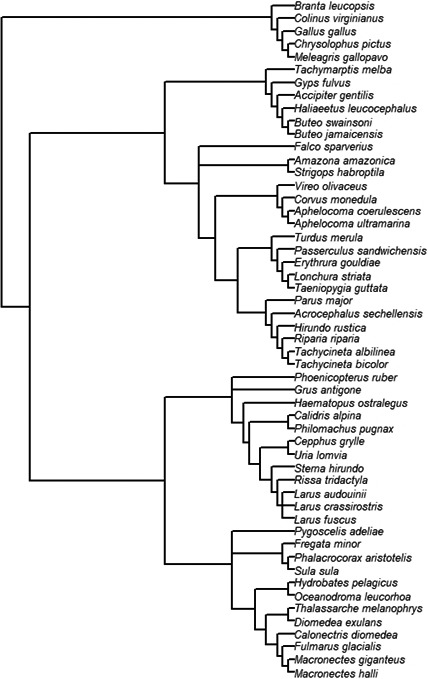
Phylogenetic tree of the 53 bird species for which adult and chick telomere length (TL) and telomere length rate of change (TROC) were collected from published papers on avian telomeres using the TRF methodology. The consensus phylogenetic tree was obtained from BirdTree.org (100 trees) using ape, apTreeshape, and caper R packages (see ESM 4)

The 53 species included in our study comprised 13 orders and 29 families (Figure 1). M*aximal life span* ranged from 6 to 60 years, *female adult body mass* ranged from 11 to 8,060 g, and *telomere length* ranged from 1.7 to 49.3 kb. Using BirdTree, we downloaded 100 trees from http://www.bird.tree.org (De Magalhaes & Costa, 2009; Jetz et al., 2012), focusing on the 53 species of our dataset (producing a .*nex* tree file). We checked the validity of this first phylogenetic representation by building an additional tree based on DNA sequences of cytochrome b (ESM3). Both alternatives produced congruent trees, but we based our subsequent analyses on the bird.tree.org based tree, because we lacked a reliable cytochrome b sequence for *Larus andouinii, Larus crassirostris, Acrocephalus sechellensis,* and *Erythrura gouldiae*. A consensus tree was produced using the Ape package (Paradis et al., 2004) and Phyltools (Revell, 2012). Branch lengths were estimated using Grafen's method (Grafen, 1989). Markov chain Monte Carlo generalized linear mixed model (MCMCglmm in R) (Hadfield, 2010; Hadfield & Nakagawa, 2010) packages were used to estimate the relative amount of variation in adult telomere length and life‐history traits that could be explained by shared ancestry (*viz*., the phylogenetic tree). These last packages were also used to estimate the phylogeny content of associations between telomere and life‐history variables.

### 2.3 Statistical analyses

Full codes for statistical analyses are presented in ESM 4. Life‐history variables exhibited a variety of correlations that suggested substantial collinearity, so variable reduction was necessary. We thus conducted a principal component analysis (PCA) using the R package FactoMineR (Lê et al., 2008) to extract independent orthogonal eigenvectors (PC axes) for later phylogenetic and generalized linear mixed modeling analyses (ESM 4). We investigated the interspecific relationships between TL variables and life‐history axes as represented by the first three PC axes. We evaluated how phylogeny accounted for overall variance of TL variables and the species scores on PC1, PC2, and PC3, using linear mixed models in a Markov chain Monte Carlo environment (MCMCglmm) (Hadfield, 2010). MCMCglmm allows inclusion of phylogenetic tree files in glmm models, in a Bayesian framework. MCMCglmm also statistically “controls” for phylogenetic dependence among variables that is due to the pattern of shared evolutionary ancestry of the species, so that “phylogeny‐adjusted” patterns among variables are revealed (see (Dunn & Møller, 2014). An additional random factor that adjusted for variance in sample sizes of measurements of variables among species was included, by inserting sampling variances into the mev argument of MCMCglmm.

We conducted univariate analyses of associations of telomere variables and each of the principal component axes (body size, pace of life, and parental care) and then multivariate analyses of all 3 life‐history axes. The life‐history axes were applied in MCMCglmm models that separately examined *Adult TL*, *Chick TL,* and *TROC* (see ESM 4). Phylogeny was entered as a random factor: TL variables = [PC1 or PC2 or PC3] + 1|phylogeny + 1|mesd [adjustment for standard errors of sample sizes, $mesd=\sqrt{\frac{1}{N‐3}}$] + error. These models produced estimates of phylogenetic associations of *Adult TL, Chick TL, TROC*, and of each of the life‐history axes in the univariate analyses. Also, we examined the magnitude and significance of correlations between the 3 telomere variables and all 3 life‐history axes, in multivariate analyses. Posterior distributions of covariances were transformed into correlations (using the *posterior.cor()* function in package MCMCglmm). We were then able to evaluate the part of the correlation explained by the phylogenetic tree (i.e., phylogenetic correlation) or not (i.e., residual correlation) for each paired association of life‐history PC axes and telomere variables. Finally, using Pearson's correlation test we examined associations of TL and the PC axes in 3 different bird orders (where the sample size >8, i.e., Passeriformes, Procellariiformes, Charadriiformes). These last analyses allowed a preliminary examination of correlative relationships of TL and life‐history PC axes within specific categories of birds that shared both history and ecological features. Effect sizes of correlations followed Cohen's (1988) suggested criteria: *r* = .1, small; *r* = .3, medium; *r* = .5, large.

## 3 RESULTS

Our literature search produced 1,450 publications. Based on an initial TRF selection (*n* = 155), we extracted 36 references with TRF values not corrected for an associated variable such as body mass or age. We added two unpublished TRF data measurements obtained from colleagues (also from blood, see ESM 1 and 2). We ended with 53 species identified with mean adult TRF values (among 38 studies, some reported more than one species (e.g., Haussmann et al., 2003; Tricola et al., 2018; Whittemore et al., 2019), 29 chick TRF values, and 35 TROC values (including 19 species from Tricola et al., 2018).

### 3.1 Life‐history traits and Principal Component Analysis

Among the 53 avian species, life‐history traits exhibited several medium to strong intercorrelations (ESM 4). In particular, measures of body size and mass were associated with most variables. In order to deal with these strong multiple collinearities, we conducted a principal component analysis. The first principal component (PC1) accounted for nearly 60% of the variance among life‐history traits and as expected was strongly associated with adult female mass and other variables that reflect the body size of the species (Table 1). The second principal component (PC2) accounted for about 11% of the variance, and positively reflected reproductive and prehatching development variables, and a negative association with life span. With reproduction and life span negatively associated and statistically independent of body size, PC2 likely reflected the “slow‐fast continuum” or pace of life syndrome (reviewed by Dobson & Oli, 2007). Scores of the species on PC2 were ordered from relatively fast life histories (greater reproduction and faster prehatching growth and shorter lives) to relatively slow life histories (longer lives, lower reproduction, and lower prehatching growth). The third principal component (PC3) accounted for about 9% of the variance and primarily reflected the number of days postfledging chicks stay with their parents. We interpreted PC3 as postfledging parental investment in offspring.

**TABLE 1:** Principal component (PC) analysis of life‐history traits of 53 bird species

	PC 1	PC 2	PC 3
Eigenvalue	7.070	1.266	1.087
% variance explained	58.913	10.552	9.061
Incubation (days)	**0.886**	−0.197	−0.035
Age at fledging (days)	**0.886**	−0.116	0.012
Mean age at sexual maturity (days)	**0.792**	−0.354	0.001
Adult structural size	**0.766**	0.287	0.215
Female adult body mass (g)	**0.813**	0.326	−0.010
Maximal life span (years)	**0.779**	**−0.365**	0.159
*Posthatching growth rate (KG)*	*−0.688*	*−0.152*	*−0.091*
Embryonic growth rate (g/day)	0.723	**0.427**	−0.086
Clutch size	−0.532	**0.702**	0.189
Egg mass (g)	**0.924**	0.119	−0.150
Hatching mass (g)	**0.920**	0.245	−0.141
Postfledging parental care (days)	0.114	−0.067	**0.966**

Note

Eigenvalues and the percentage of variance explained by each component are given for the three first PC axes, representing those with an eigenvalue larger than 1 (total variance explained is 79.09%). For each variable, correlation coefficients with the principal component axis are given, with the significant values in bold (contributions > average aleatory contribution, 100/*n*, *n* = number of variables). *Posthatching growth rate* had a large effect size on PC1, but did not load significantly on any of the three PC axis (indicated in italics).

### 3.2 Analyses of TL and life‐history traits

*TROC* was significantly but moderately negatively associated with *Adult TL* and *Chick TL* (respectively; *r* = −.349, *p* = .04, *n* = 35; *r* = −.488, *p* = .015, *n* = 24). However, the association of *TROC* and *Adult TL* was strongly influenced by two extreme points, *Larus Andouiin*i and *Parus major*, which presented long telomere lengths and high rates of annual erosion (ESM 1). If we exclude these two extremes, the association changed in sign and became nonsignificant (*r* = .127, *p* = .48, *n* = 33). *Adult TL* and *Chick TL* showed a strong positive association (*r* = .882, *p* < .0001, *n* = 28).

**FIGURE 2:**
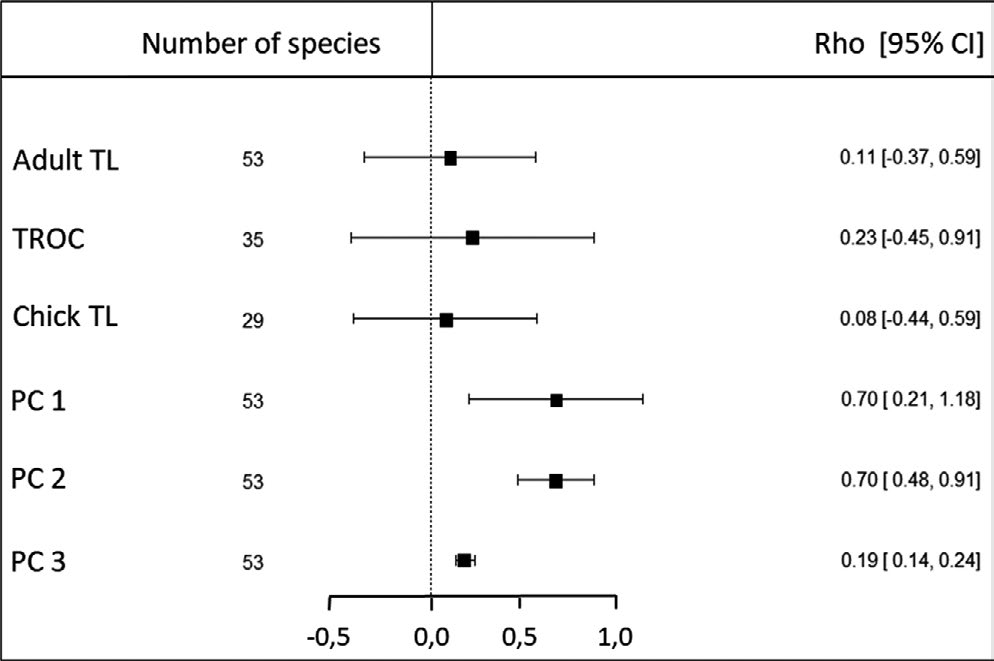
Effect sizes (Rho values) and 95% confidence intervals for phylogenetic effects on bird TL variables (Adult telomere length (Adult TL), telomere length rate of change (TROC), and chick telomere length (Chick TL), and the three principal component axes that reflected bird species' life‐history traits (PCA 1—*body size*, PCA 2—*slow–fast continuum* of the *pace of life*, and PCA 3—*postfledging parental care*). Effects are considered significant when not overlapping zero

Using the scores of the different avian species on the principal components, we investigated the associations of TL with the three statistically independent life‐history axes. The three telomere variables (*Adult TL*, *TROC,* and *Chick TL*) showed small‐to‐medium and nonsignificant associations with phylogeny (Figure 2). Body size and the slow–fast continuum of pace of life (PC1, PC2) were strongly and significantly associated with the phylogenetic pattern. Parental care (PC3) exhibited a small‐to‐medium association with phylogeny, but a significant one.

**FIGURE 3:**
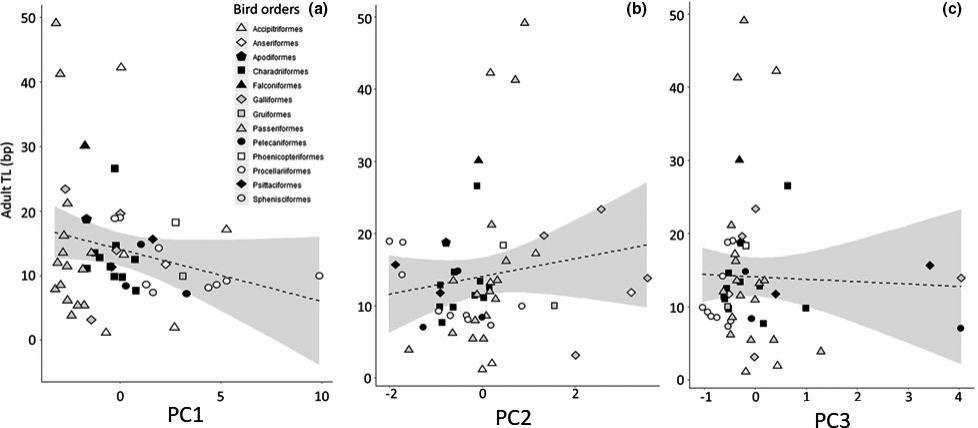
Adult telomere length (TL, in kb) in relation to life‐history axes PC1 (a), PC2 (b), and PC3 (c). Note that none of the relationships turned out to be significant (Table 2). Dashed lines refer to Pearson's linear correlation (PC1, *r* = −.23; PC2, *r* = .15; PC3, *r* = .04, Table 2). Symbols indicate the distribution of the 13 bird orders. Note that two unadjusted correlations reached significance for adult TL/PC2: Procellariiformes, *r* = −.75, *t* = −2.18, *p* = .031, *n* = 8; Passeriformes, *r* = .69, *t* = 3.32, *p* = .006, *n* = 14

**FIGURE 4:**
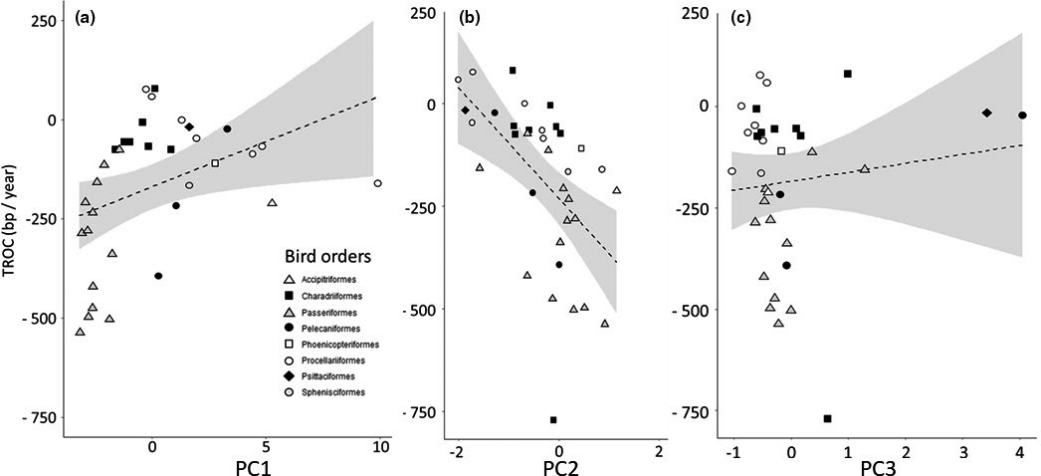
Telomere length rate of change (TROC, in kb/year) in relation to life‐history axes PC1 (a), PC2 (b), and PC3 (c). Only the TROC and PC2 relationship was statistically significant (Table 2). Dashed (nonsignificant) and plain (significant) lines refer to unadjusted for phylogeny Pearson's linear correlation (PC1, *r* = .34; PC2, *r* = −.54; PC3, *r* = .12, Table 2). Symbols indicates the distribution of the 13 bird orders. Note that all unadjusted correlations reached significance for the Procellariiformes, *n* = 7; PC1: *r* = −.95, *t* = −6.97, *p* < .001; PC2: *r* = −.92, *t* = −5.06, *p* = .004; PC3: *r* = −.79, *t* = −2.86, *p* = .035

**FIGURE 5:**
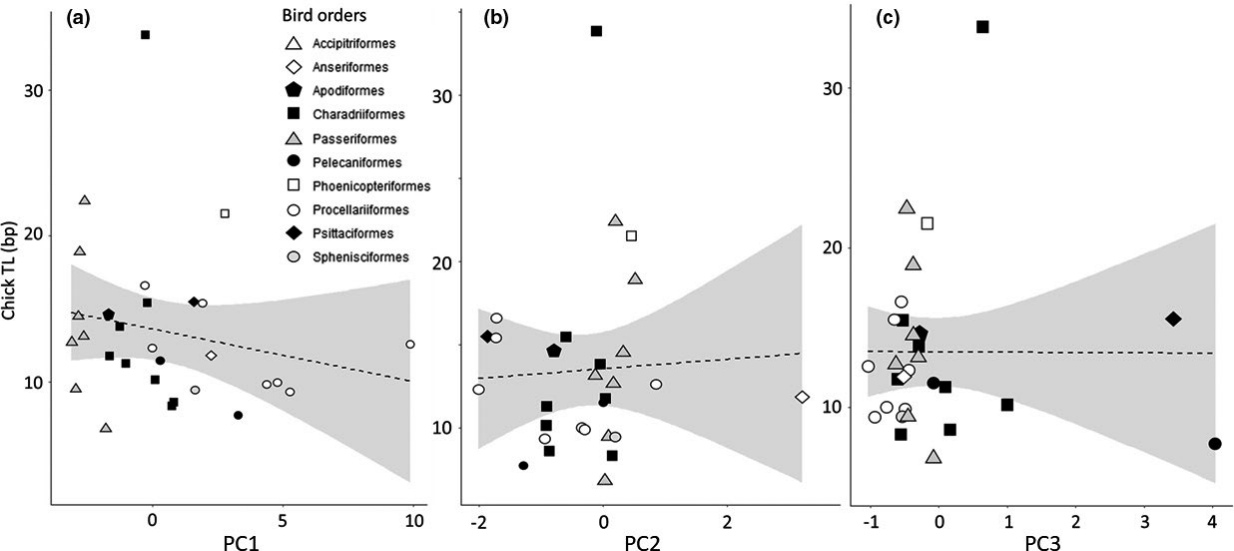
Chick telomere length (TL, in kb) in relation to life‐history axes PC1 (a), PC2 (b), and PC3 (c). Note that none of the relationships turned out to be significant (Table 2). Dashed lines refer to unadjusted for phylogeny Pearson's linear correlation (*n* = 29; PC1, *r* = −.20; PC2, *r* = .05; PC3, *r* = −.01, Table 2). Symbols indicates the distribution of the 13 bird orders

We then investigated the associations of bird TL with the three principal component axes (that displayed phylogenetic patterns) using three different approaches. First, we explored direct Pearson's correlations (without considering any phylogenetic effect) between TL variables and the principal components. This suggested a small‐to‐medium and nonsignificant correlation of *Adult TL* with body size (PC1; Table 2a, Figure 3a). *TROC* exhibited a medium positive association with body size (PC1) and a large negative association with the slow–fast pace of life (PC2; species with greater reproduction and shorter life exhibited more strongly negative *TROC*; Table 2b, Figure 4a,b). Correlations were at best small and nonsignificant between *Chick TL* and the life‐history axes (Table 2c, Figure 5).

**TABLE 2:** For 53 bird species, analyses of associations of telomere traits (adult mean telomere length (a, *Adult TL*), telomere rate of changes (b, *TROC*), and chick mean telomere length (c, *Chick TL*) and life‐history traits (body size, PC1; the slow–fast continuum of pace of life, PC2; and parental care, PC3)

	Unadjusted Pearson's correlation	Univariate MCMC model correcting for phylogeny		Multivariate MCMC model correcting for phylogeny			
		Phylogenetic *r*	Residual *r*	Beta	*p* value	Phylogenetic *r*	Residual *r*
(a) Adult TL (53 species)							
PC1	−0.228 (−0.469 to 0.045)	−0.659	−0.153	−0.857	.14	−0.797 (−0.948 to 0.603)	−0.154 (−0.462 to 0.248)
PC2	0.147 (−0.128 to 0.401)	0.490	0.384	1.851	.19	0.075 (−0.824 to 0.806)	0.196 (−0.147 to 0.596)
PC3	−0.036 (−0.303 to 0.237)	0.226	−0.082	−0.148	.91	−0.225 (−0.805 to 0.866)	−0.035 (−0.418 to 0.218)
(b) TROC (35 species)							
PC1	**0.344 (0.012 to 0.608)**	0.905	−0.108	23.256	.067	0.746 (−0.468 to 0.967)	−0.186 (−0.530 to 0.336)
PC2	**−0.541 (−0.740 to −0.253)**	−0.952	**−0.531**	−154.465	**<.001**	−0.886 (−0.979 to 0.358)	**−0.465 (−0.733 to 0.008)**
PC3	0.117 (−0.225 to 0.434)	−0.648	0.199	154.465	.351	−0.650 (−0.869 to 0.740)	0.250 (−0.203 to 0.538)
(c) Chick TL (29 species)							
PC1	−0.198 (−0.526 to 0.182)	−0.846	−0.057	−0.362	.416	−0.439 (−0.949 to 0.665)	−0.059 (−0.465 to 0.336)
PC2	0.053 (−0.319 to 0.412)	0.771	0.178	0.421	.732	−0.451 (−0.862 to 0.846)	0.179 (−0.365 to 0.438)
PC3	−0.004 (−0.370 to 0.363)	−0.030	0.028	0.125	.924	0.306 (−0.830 to 0.860)	0.004 (−0.353 to 0.365)

Note

Pearson's correlation coefficients and both univariate and multivariate MCMC models that measure effects of phylogeny and statistically “adjust” for shared ancestry are shown. Proportion of variance among species attributed to phylogeny (phylogenetic *r*), and unexplained associations (residual *r*, associations statistically “controlled” for phylogeny) are presented, with significant results in bold. Beta coefficients show changes in TL measures in kb for each standardized unit change in the principal component axis values.

Next, univariate analyses that statistically adjusted both telomere variables and life‐history data for the phylogenetic pattern confirmed only some of the ahistorical correlational analyses. First, it revealed that the relatively weak pattern of association of adult TL and body size was likely due to the phylogenetic pattern (the residual “phylogeny‐adjusted” correlation was −0.153; Table 2a). The same was evident for the moderate positive association of *TROC* with body size (the residual correlation was −0.108; Table 2b). The negative association of *TROC* and the slow–fast pace of life was confirmed as a strong pattern. Association between *TROC* and life‐history axes exhibited especially strong phylogenetic patterns.

**FIGURE 6:**
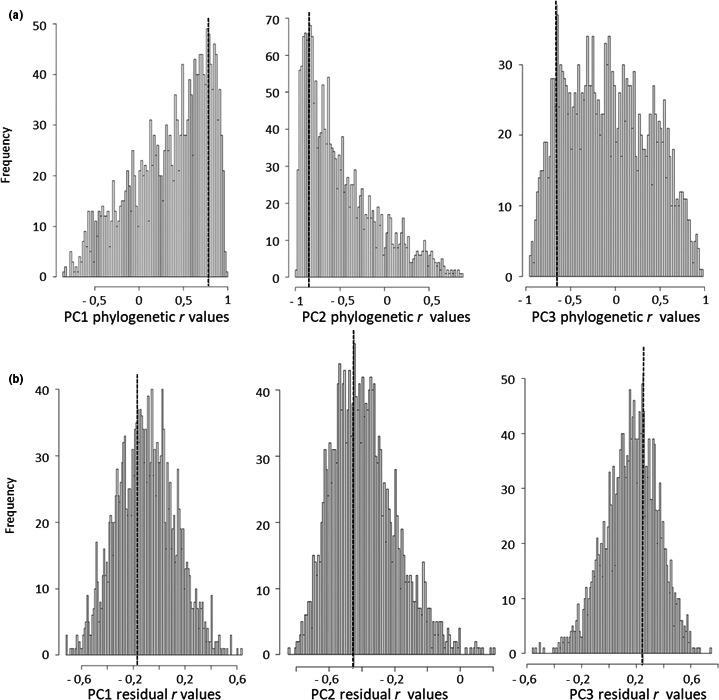
Frequency distributions of phylogenetic (a) and residual (b) correlation values produced by the iterations of the multivariate linear models using Monte Carlo methods and Bayesian Markov chain sampling (MCMC) between TROC and life‐history axes of bird life‐history traits, that is, PC1 (body size, left panels), PC2 (pace of life, middle panels), and PC3 (parental care, right panels). This multivariate model controlled for shared ancestry simultaneously for all variables. Black lines indicate the peak values reported in Table 2 (two last right columns)

We next examined these potential associations while considering the effect of phylogeny on both variance and covariance of *TL* and PC variables, using a multivariate phylogenetic framework. The correlation between PCs and *TL* variables due to phylogeny was marginal, since their posterior distributions mostly were broadly centered on or largely overlapped zero (Table 2, Figure 6). Only the negative association of *TROC* and slow–fast pace of life approached significance, and it again exhibited a strong phylogenetic pattern (Table 2b; Figure 6, center panels).

Finally, we investigated whether relationships among PC axis and TL variables might differ among avian orders. We found that the association of *Adult TL* and the slow–fast pace of life (PC2) was significantly positive in the Passeriiformes (*r* = .690, *t* = 3.321, *p* = .006, *n* = 14). However, in the Procellariiformes the association was negative (*r* = −.754, *t* = −2.812, *p* = .031, *n* = 8), that is, opposite to the general nonsignificant interspecific trend (Figure 3b). *TROC* and PC1 and PC2 axes were significantly and negatively correlated in the Procellariiformes (Figure 4; PC1: *r* = −.951, *t* = −6.872, *p* < .001; PC2: *r* = −.890, *t* = −4.368, *p* = .007; both *n* = 7). No significant correlations were found within orders for PC axes and *Chick TL* (Figure 5, all *p* > .25).

## 4 DISCUSSION

Interest in telomeres has steadily increased in the last decade. Telomere length, as a result of the interaction between telomere loss and DNA maintenance processes like telomerase activity (an enzyme which actively rebuilds the ever‐shortening telomeres; Greider & Blackburn, 1985), has been linked to individual health, cellular aging, and organismal senescence (e.g., Bernardes de Jesus & Blasco, 2012; Verhulst et al., 2016). This has triggered additional interest in understanding how telomere length and its rate of change are related, and the extent to which both may predict longevity or fitness at different periods of life (Bichet et al., 2020; Salomons et al., 2009; Vera et al., 2012). Such links at the species level have been previously studied (Dantzer & Fletcher, 2015; Tricola et al., 2018; Whittemore et al., 2019). Our study aimed to disentangle the phylogenetic and nonphylogenetic correlations among *Adult TL, Chick TL, TROC,* and three general bird life‐history traits.

### 4.1 Phylogenetic patterns in life‐history traits

For our 53 species of birds, life‐history traits were largely represented on three orthogonal axes: *body size* (PC1), reproductive and longevity features along a *slow–fast continuum* of the *pace of life* (PC2), and *postfledging parental care* (PC3). Our interpretations of life‐history axes followed well‐accepted principal based on previous studies of vertebrate species (Gaillard et al., 1989; Harvey & Purvis, 1999; Stearns, 1992). The first axis of the PCA clearly reflected the magnitude of collinear life‐history traits, usually interpreted as reflecting variation among species in body size. This conclusion is supported by positive loadings over PC1 for adult structural size and body mass, as well as egg and hatching mass. Some body size‐related traits that reflect duration of life also had strong positive loadings on PC1, such as incubation duration, age at fledging, age at sexual maturity, and maximal life span (Speakman, 2005a). The second axis of the PCA appeared to reflect the *slow–fast continuum*. Eigenvectors built within a PC analysis are orthogonal to each other; thus, the species‐specific trade‐off between reproduction (clutch size, a positive factor loading) and survival (age at sexual maturity and maximal life span, negative factor loadings) on PC2 were statistically independent of the species' adult body sizes (the latter reflected by loadings on PC1). As investment in reproduction decreases, age at maturity increases, and life span increases, an interspecific pattern that is consistent with a fundamental life‐history trade‐off. Longevity thus has two components, that associated with body size and a residual component associated with the pace of life. Finally, the third axis of the PCA had a strong factor loading only for the duration of postfledging parental care, that is, the number of days parents care for their offspring once they have left the nest. This axis was statistically independent of both body size and the slow–fast continuum.

Unsurprisingly, variance among species for the three PC axes of life‐history traits was strongly associated with the species' phylogenetic histories. Associations of life history with phylogeny are well documented in vertebrate animals (Dobson & Jouventin, 2007; Dobson & Oli, 2007; de Magalhaes et al., 2007). As “body plans” are associated traits that change over evolutionary time, life‐history traits are expected to coevolve (Bennett & Owens, 2002; Brown & Sibly, 2006; Roff, 2002; Stearns, 1992). In mammals, a relatively strong trade‐off is found between growth and longevity, long‐lived species showing slower postnatal growth rates. In birds, there is strong selection for rapid growth after hatching, in order to quickly reach emancipation (thermal and nutritional) (de Magalhaes et al., 2007). While we found that embryonic growth rate showed the expected negatively related pattern of variation with life span along the slow–fast continuum, posthatching growth rate was relatively low for the largest species and not associated with the slow–fast continuum. This suggests that embryonic growth rate may have a larger effect on life span evolution than posthatching growth rate. Short‐lived birds were characterized by faster embryonic development, a relationship previously characterized for vertebrates in general (Ricklefs, 2006). Such observations need to be remembered when further focusing at how life‐history axes relate to *Adult TL* (see below), since the mechanisms of the delayed cost of rapid growth in terms of impaired somatic maintenance and shortened life span are not well understood (Monaghan & Ozanne, 2018).

### 4.2 Phylogenetic patterns in telomere lengths and TROC

In analyses of associations of TL values and life‐history axes, strong phylogenetic values indicate that correlations are not independent over the history of the species. Species that are closely related may show similar patterns. A good example was the correlation between TROC and the body size axis (PC1), for which the “phylogeny‐free” correlation was actually different in sign from the unadjusted correlations (note the high phylogenetic correlation; Table 2b). Alternatively, different clades may show different patterns, such as the *Adult TL* and pace of life (PC2) correlation in the Passeriiformes (positive) and in the Procellariiformes (negative) (note the high phylogenetic correlation; Table 2a). After adjustment for phylogeny, residual correlations reveal ahistorical associations, such as the fairly strong negative association of TROC and PC2 (see below and Table 2b).

*Adult* and *Chick TL* among species were poorly explained by phylogeny, while TROC showed the strongest phylogenetic signal (Figure 2). Life‐history axes, however, were strongly associated with phylogenetic history. Our result from 53 avian species confirms and extends previous work done on 19 bird species (Tricola et al., 2018). These results are important to our understanding of how life histories and telomere length on the one hand, and the proximate factors that modulate telomere erosion on the other hand, are inter‐related. It suggests that similarity in telomere lengths between species is not primarily due to shared ancestry. Moreover, there may be independent evolutionary processes that have operated within different avian orders and families, leading to the present diversity in telomere lengths. If the evolution of life‐history traits is strongly constrained by phylogeny, but *Adult* and *Chick TL* are not, it may not be surprising that PC1, 2, and 3 showed only weak associations with telomere lengths measured at both stages of life. However, even after controlling for phylogeny, we found that the remaining variance in PC axes and *Adult or Chick TL* was poorly related (Table 2). This supports the hypothesis that telomere length evolved short and long forms at both life stages, even in closely phylogenetically related species that share comparable life‐history traits. Our results seem to accord with a low phylogenetic pattern of telomere lengths in mammals (Gomes et al., 2011; Seluanov et al., 2007). For *TROC*, the apparent high variability in telomerase activity in closely related species (Gomes et al., 2011; Tian et al., 2018) suggests that there should be little phylogenetic pattern, as we found.

We found no relationship between PC axes and *Chick TL*. We expected slow‐living species to exhibit more preserved and then longer telomere lengths at the end of growth, given that rapid growth rate may enhance telomere shortening (Monaghan & Ozanne, 2018; Vedder et al., 2018). However, it is too early for conclusions about the coevolution of growth and telomere dynamics at the chick stage. Given that telomere lengths in early life are a complex mix of inherited traits and environmental conditions (Dugdale & Richardson, 2018; Eisenberg, 2019; Vedder et al., 2017), more data are needed on embryonic telomere lengths. Further comparative analyses should include ecological variables that may highlight how specific local conditions can influence *Chick TL*.

*TROC* (calculated over the chick to adult stages) appeared moderately (our data) or unrelated to mean adult telomere length, such that most species with long telomere lengths after growth were not showing high rates of *bp* loss per year (Tricola et al., 2018). This result did not support the hypothesis that long telomeres erode faster because they are more sensitive to damage, nor that species with short telomeres should exhibit lower TROC over adulthood (as suggested by intraspecific research (Bize et al., 2009; Grasman et al., 2011; Salomons et al., 2009)). Whether this could be the case for *TROC* calculated from earlier development remains an open question, and the fact that we found a negative relationship between *TROC* and *Chick TL* suggests that, during growth, the mechanisms regulating telomere dynamics are different than those in play during adulthood. As for *TROC* within adults and chicks, these may reflect distinct and nonadditive processes defining species and trade‐offs of individual growth, short‐term chick survival, and adult life span (Boonekamp et al., 2014), through a tightly regulated balance between energy investment in telomere maintenance and somatic growth. In addition, our results suggest a coevolution of low *TROC* with increasing body size along the bird phylogenetic tree. This conflict indicates that more detailed studies are needed on how body size, embryonic development, and reproduction investments may be related to specific telomere dynamics. However, the strong phylogenetic correlation of *TROC* and PC1 left little evidence of a relationship between these variables under current environmental conditions. In short, the strong phylogenetic pattern rendered the apparent positive phenotypic correlations of TROC and PC1 nonsignificant and slightly negative.

*Adult TROC* was closer to zero in species that produced smaller clutches and exhibited low embryonic growth rates on the slow–fast continuum (more negative values on the PC2 axis). Of all the potential associations of life histories and telomere dynamics, the association of slow growth and low reproductive rate with less TROC was the only consistent and significant pattern that we found, whether phylogenetically adjusted or not, and univariate or multivariate. Little TROC in slow‐growing species with low clutch sizes might be maintained by genetic correlations among these traits. Such negative associations between life‐history traits that divert energy from body maintenance and TROC may reflect two mechanisms of selection. First, the selective disappearance of individuals that were submitted to rapid telomere loss early in life, either due to unfavorable growth trajectories or unbalanced reproductive investments. This has been extensively discussed previously (Dantzer & Fletcher, 2015; Tricola et al., 2018): Within individuals, *TROC* values were significantly higher (i.e., a greater rate of loss) than between‐individuals, suggesting the selective disappearance of individuals with short telomeres. However, comparisons of between‐ and within‐individuals TROC relationships with life span were found to be consistent, suggesting that selective disappearance might have a weak effect at the interspecific level. The second possibility is that less *TROC* actually reflects the coselection of physiological or cellular mechanisms that protect telomere ends from a rapid erosion. In our study, the *TROC*–PC2 relationship was independent of body size effects, suggesting that it is not specifically in the largest birds that a coevolution of slow growth and low reproductive rates with less TROC has taken place. Because telomerase is expected to be inactive in somatic cells soon after birth in large‐bodied species (Seluanov et al., 2007), the underlying mechanisms should be independent of telomerase.

### 4.3 TROC in the Procellariiformes and the pace of life

In mammals, shorter telomeres prevailed in large‐bodied and long‐lived species (Gomes et al., 2011; Risques & Promislov, 2018), and short telomeres are associated with repressed telomerase activity in somatic cells at adulthood (Tian et al., 2018). In contrast, we found that bird *Adult TL* was not related to the body size PC1 axis. The variance in body sizes within birds is less extensive than for mammals, which might partially explain the difference observed between these two major clades of vertebrates; that is, too low a range in body size of birds to reveal strong negative selection on telomere length. Adjustment for the phylogenetic tree reduced the association to a negligible value (Table 2). Still, our within‐orders regression data suggested negative relationships between *Adult TL* and body size, and *TROC* and both body size and the slow–fast continuum in Procellariiformes, an order with the extensive range of body sizes of long‐lived seabirds. The former relationship suggests that, as previously found in rodents, the small‐ and long‐lived species in the *Hydrobatidae* may have evolved specific mechanisms promoting long life span while preserving telomerase activity and long telomeres over life (Tian et al., 2018). The latter link reveals that the relatively long‐living and small‐bodied Procellariiformes exhibit long telomeres in adulthood (*Oceanodroma leucorhoa* and *Hydrobates pelagicus* (both *Hydrobatidae*) and to a lesser extent *Fulmarus glacialis*)) along with particularly slow embryonic growth rates (respectively, 0.104, 0.166, and 1.181 vs. a mean procellariform value of 1.938 g/day; note that variation in clutch size along the slow–fast continuum is negligible for procellariiform species because they lay only a single egg each breeding season; ESM1). Thus, long telomeres in this order were not significantly related to body size (PC1), but were related to the slow end of the slow–fast continuum (PC2) and thus to species longevity. This relationship is reversed in the Passeriformes, where long telomeres were related to the fast (short life span) end of the slow–fast continuum of life history.

One of the pathways that could decelerate the rate of cell division, and hence the rate of telomere erosion (Monaghan & Ozanne, 2018), is a prolonged incubation duration (Vedder et al., 2018). Prolonged incubation might also be associated with the maintenance of high telomerase activity throughout life in procellariiform species (Haussmann et al., 2004). An alternative explanation could be that the *Hydrobatidae*, which are cavity nesters where chicks grow in a very contained and stable environment, even after hatching, may exhibit metabolism that favors the maintenance of the lengths of the telomeres (Stier et al., 2019). This pattern supports the antagonistic pleiotropy of telomere length processes, the main hypothesis proposed to explain why species in different phylogenetic clades have evolved large differences in telomere lengths (Hemann & Greider, 2000). This perspective deserves future exploration, notably on telomerase activity in the soma among the species of Procellariformes.

## CONFLICT OF INTEREST

None declared.

## AUTHOR CONTRIBUTIONS

**François Criscuolo:** Conceptualization (equal); data curation (lead); formal analysis (equal); writing‐original draft (equal); writing‐review & editing (equal). **F. Stephen Dobson:** Conceptualization (equal); formal analysis (equal); writing‐original draft (equal); writing‐review & editing (equal). **Quentin Schull:** Conceptualization (equal); formal analysis (equal); writing‐original draft (equal); writing‐review & editing (equal).

## Supporting information

Supplementary Material

Supplementary Material

Supplementary Material

Supplementary Material

## Data Availability

Data file is provided in the ESM1.
