#  

**DOI:** 10.1002/ece3.9214

**Published:** 2022-09-14

In the paper published by Criscuolo et al. (2021), the abstract erroneously reported “Phylogeny had at best a small‐to‐medium influence on Adult and Chick TL (*r* ^2^ = .190 and .138, respectively), but a substantial influence on TROC (*r* ^2^ = .688). Phylogeny strongly influenced life histories: PC1 (*r* ^2^ = .828), PC2 (.838), and PC3 (.613). Adult TL and Chick TL were poorly associated with the life‐history variables. TROC, however, was negatively and moderate‐to‐strongly associated with PC2 (unadjusted *r* = −.340; with phylogenetic correction, *r* = −.490)”; which must be replaced by: “Phylogeny had at best a small‐to‐medium influence on Adult TL, TROC, and Chick TL (*r* ^2^ = .11, .23, and .08, respectively). Phylogeny had strong‐to‐mild influences on life histories: PC1, PC2, and PC3 (*r* ^2^ = .70, .70, and .19, respectively). Adult TL and Chick TL were poorly associated with the life‐history variables. TROC, however, was negatively and moderate‐to‐strongly associated with PC2 (unadjusted *r* = −.541; with phylogenetic correction, *r* = −.531).”

A second error concerned the use of *Hydrobates pelagicus* as one of the 53 species for which telomere data originated from Figure 1 of *Monaghan, P., & Haussmann, M. (2006). Do telomere dynamics link lifestyle and lifespan? Trends in Ecology & Evolution, 21(1), 47–53*. 10.1016/j.tree.2005.11.007. It appears that those data originated from *Oceanodroma leucorhoa* from *Haussmann, M. F., Winkler, D. W., O'Reilly, K. M., Huntington, C. E., Nisbet, I. C. T., & Vleck, C. M. (2003). Telomeres shorten more slowly in long‐lived birds and mammals than in short‐lived ones. Proceedings of the Royal Society B: Biological Sciences, 270(1522), 1387–1392*. 10.1098/rspb.2003.2385. As a consequence, our study is based on 52 species and not 53. We reran the MCMCglmm analyses using the corrected file, and found only trivial changes to results and no changes in the conclusions of the study. This is indicated in the new 2022‐06‐27_MS_script_V3_correction file.

The authors apologize for this oversight.
