# Supplementary material for: The influence of phylogeny and life history on telomere lengths and telomere rate of change among bird species: A meta‐analysis
Source: Ecol Evol. 2021 Sep 10;11(19):12908–22. doi: 10.1002/ece3.7931 (PMC8495793; doi:10.1002/ece3.7931)
Supplement: Supplementary file 2 — Supplementary Material [file ECE3-11-12908-s001.docx]

**The influence of phylogeny and life history on telomere lengths and telomere rate of change among bird species: a meta-analysis**

Criscuolo F.^1‡^, Dobson F.S^1,2‡^ and Q. Schull ^3^

^1^ University of Strasbourg, CNRS, IPHC UMR 7178, 67000 Strasbourg, France.

^2^Auburn university, Department of Biological sciences, Auburn, AL, USA

^3^ MARBEC, University of Montpellier, IFREMER, IRD, CNRS, Sète, France

^‡^ Authors for correspondence ([francois.criscuolo@iphc.cnrs.fr](mailto:francois.criscuolo@iphc.cnrs.fr), [fsdobson@msn.com](mailto:fsdobson@msn.com))

**Electronic Supplementary Material 2: Life history and telomere length data collection**

**Life history traits**

Life history variables were selected as follows: the observed *maximal lifespan* (AnAge database, [[1](#_ENREF_1)], <http://genomics.senescence.info/species/>), the *post-hatching and embryonic growth rates*, *female adult body mass and mean adult body size* [[2](#_ENREF_2), [3](#_ENREF_3)], *egg mass* and *hatching mass* [[4-9](#_ENREF_4)], *mean age at sexual maturity* (age at which an individual is physiologically able to reproduce), *clutch size, incubation duration* and *age at fledging* (when chicks leave the nest) [[10](#_ENREF_10), [11](#_ENREF_11)], and *post-fledging parental care* (as the number of days fledged chicks stay with their parents) [[12](#_ENREF_12)]. All those data were crossed-checked with sources of general information on the species’ biology [[1](#_ENREF_1), [12](#_ENREF_12), [13](#_ENREF_13)]. We also examined previous reviews using a meta-analysis approach on bird growth rate, longevity or telomere length to build our dataset [[14-16](#_ENREF_14)]. When records differed between sources, we choose to take the mean value for all variables, except the *observed maximal lifespan* for which we took the highest recorded value. *Observed maximal lifespan* was previously shown to be strongly related to telomere biology in birds [[15](#_ENREF_15)] and is likely to be more representative of endogenous ageing processes than mean lifespan, being independent of exogenous causes of mortality (*e.g.* predation). Available data for adult female body mass was missing for 6 species, for which we relied on mean adult body mass (*Chrysolophus pictus*, *Amazona amazonica*, *Aphelocoma ultramarina*, *Cepphus grylle*, *Tachymarptis melba*, *Uria lomvia*).

**Bird telomere length**

When data were only available in the form of figures, we extracted the values using GetData Graph Digitizer 2.26 (<http://www.getdata-graph-digitizer.com>). In addition, unpublished data were requested from researchers for two species, the Alpine swift (*Apus melba*, P. Bize pers.com.) and the Barnacle goose (*Branta leucopsis*, A. Pauliny pers.com.). Sample size for *Adult TL* varied from 1 to 104 (mean value ± SE, 20.9 ± 2.9, ESM 1). Telomere length data collected consisted of average value *per* species within adult life stage, but we did not include age-class because this would have reduced the sample size drastically. Therefore, we did not include age in our analysis of telomere length – life history / growth patterns, which may reduce (but not increase) our chances of detecting biological patterns. However, we conducted a separate analysis on *Chick TL* for those species were the data were available, and an additional one on telomere length rate of change (TROC). Avian telomeres have the peculiarity of being distributed over three classes of sizes, from kb to Mb [[17](#_ENREF_17)], but TRF measurements on which we relied do not use Mb sizes. However, ultra-long telomeres may generally shorten more during the nestling stage than the adult stage [[18](#_ENREF_18)], thereby being of less interest in our study. This methodological limit must be kept in mind, with the global idea that the rate of telomere shortening, which leads to the shortest telomeres, has been previously shown to be related both to embryonic growth rate in common terns (*Sterna hirundo*) [[19](#_ENREF_19)] and to maximum lifespan in birds [[15](#_ENREF_15)]. Dunlin and Sand martin TRF are from [[20](#_ENREF_20)], for which the method of TRF measurement was since then criticized [[21](#_ENREF_21)]. However, running our analysis without those two species did not change the significant results, and we chose to keep them in our study. We also check that data from old studies using traditional Southern blot technique (*e.g.* [[17](#_ENREF_17)]) instead of the more recent in-gel hybridization did not alter the significance of the presented results.

**Figure ESM 2**

**
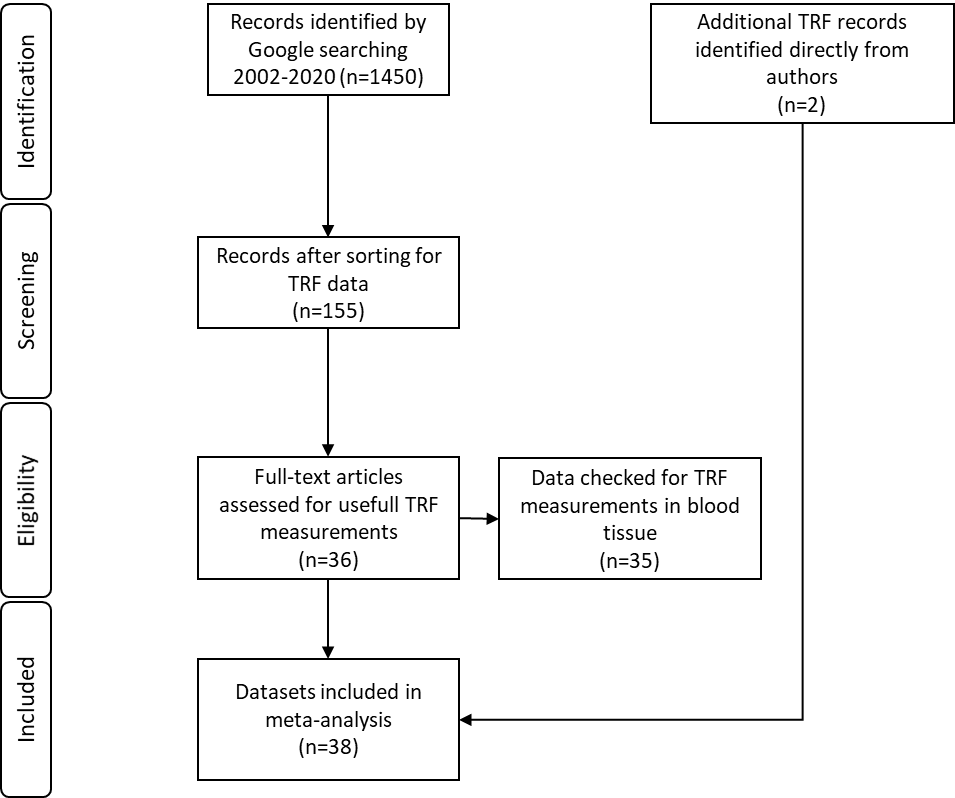
**

**Flow diagram describing the different phases of selection of studies and dataset of telomere length values determined by telomere restriction fragment (TRF) method (n = number of studies).**

**References**

[1] Tacutu, R., Craig, T., Budovsky, A., Wuttke, D., Lehmann, G., Taranukha, D. & de Magalhaes, J. 2013 Affinage: The Animal Ageing and Longevity Database. On-line. . *Human Genomic Resources: Integrated databases and tools for the biology and genetics of ageing.* **Accessed May, 4, 2013.**

[2] Ricklefs, R.E. 2010 Embryo growth rates in birds and mammals. *Functional Ecology* **24**, 588-596. (doi:10.1111/j.1365-2435.2009.01684.x).

[3] Royle, N.J., Hartley, I.R., Owens, I.P.F. & Parker, G.A. 1999 Sibling competition and the evolution of growth rates in birds. *Proceedings of the Royal Society B: Biological Sciences* **266**, 923-932.

[4] Carey, C., Garber, S.D., Thompson, E.L. & James, F.C. 1983 Avian reproduction over an altitudinal gradient. II. Physical characteristics and water loss of eggs. *Physiological Zoology* **56**.

[5] Dyke, G.J. & Kaiser, G.W. 2010 Cracking a developmental constraint: egg size and bird evolution. In Proceedings of the VII International Meeting of the Society of Avian Paleontology and Evolution, ed. W.E. Boles and T.H. Worthy. *Records of the Australian Museum* **62**, 207-216. (doi:10.3853/j.0067-1975.62.2010.1547).

[6] Ar, A., Rahn, H. & Paganelli, C. 1979 The avian egg: mass and strength. *Condor* **81**, 331-337.

[7] Rahn, H., Paganelli, C. & Sotherland, P.R. 1985 Initial mass of avian eggs / comparison between measured and calculated values. *Journal of Ornithology* **126**, 210-212.

[8] Rahn, H., Sotherland, P.R. & Paganelli, C. 1985 Interrelationships between egg mass and adult body mass and metabolism among passerine birds. *Journal of Ornithology* **126**, S263-S271.

[9] Sotherland, P.R. & Rahn, H. 1987 On the composition of bird eggs. *The Condor* **89**, 48-65.

[10] Storchová, L. & Hořák, D. 2018 Life-history characteristics of European birds. *Global Ecology and Biogeography* **27**, 400-406. (doi:10.1111/geb.12709).

[11] Sibly, R.M., Witt, C.C., Wright, N.A., Venditti, C., Jetz, W. & Brown, J.H. 2012 Energetics, lifestyle, and reproduction in birds. *Proceedings of the National Academy of Sciences* **109**, 10937-10941. (doi:10.1073/pnas.1206512109).

[12] Del Hoyo, J., Elliott, A. & Sargatal, J. 1992 Handbook of the birds of the world (Vol. 1, No. 8). Barcelona: Lynx edicions.

[13] Tacutu, R., Budovsky, A. & Fraifeld, V.E. 2010 The NetAge database: a compendium of networks for longevity, age-related diseases and associated processes. *Biogerontology* **11**, 513-522. (doi:10.1007/s10522-010-9265-8).

[14] Wilbourn, R.V., Moatt, J.P., Froy, H., Walling, C.A., Nussey, D.H. & Boonekamp, J.J. 2018 The relationship between telomere length and mortality risk in non-model vertebrate systems: a meta-analysis. *Philosophical Transactions of the Royal Society B: Biological Sciences* **373**, 20160447. (doi:10.1098/rstb.2016.0447 10.6084/m9).

[15] Tricola, G.M., Simons, M.J.P., Atema, E., Boughton, R.K., Brown, J.L., Dearborn, D.C., Divoky, G., Eimes, J.A., Huntington, C.E., Kitaysky, A.S., et al. 2018 The rate of telomere loss is related to maximum lifespan in birds. *Philosophical Transactions of the Royal Society B: Biological Sciences* **373**, 20160445. (doi:10.1098/rstb.2016.0445).

[16] Monaghan, P. & Ozanne, S.E. 2018 Somatic growth and telomere dynamics in vertebrates: relationships, mechanisms and consequences. *Philosophical Transactions of the Royal Society B: Biological Sciences* **373**, 20160446. (doi:10.1098/rstb.2016.0446).

[17] Delany, M.E., Krupkin, A.B. & Miller, M.M. 2000 Organization of telomere sequences in birds: evidence for arrays of extreme length and for in vivo shortening. *Cytogenetics and Cell Genetics* **90**, 139-145.

[18] Atema, E., Mulder, E., Van Noordwijk, A.J. & Verhulst, S. 2018 Ultra-long telomeres shorten with age in nestling great tits but are static in adults and mask attrition of short telomeres. *Molecular Ecology Resources* **19**, 648-658.

[19] Vedder, O., Verhulst, S., Zuidersma, E. & Bouwhuis, S. 2018 Embryonic growth rate affects telomere attrition: an experiment in a wild bird. *The Journal of Experimental Biology* **221**, jeb181586. (doi:10.1242/jeb.181586).

[20] Pauliny, A., Wagner, R.H., Augustin, J., SzÉP, T. & Blomqvist, D. 2006 Age-independent telomere length predicts fitness in two bird Species. *Molecular Ecology* **15**, 1681-1687. (doi:10.1111/j.1365-294X.2006.02862.x).

[21] Haussmann, M.F., Salomons, H.M. & Verhulst, S. 2011 Telomere measurement tools: Telometric produces biased estimates of telomere length. *Heredity* **107**, 371-371. (doi:10.1038/hdy.2011.10).
