# Supplementary material for: The influence of phylogeny and life history on telomere lengths and telomere rate of change among bird species: A meta‐analysis
Source: Ecol Evol. 2021 Sep 10;11(19):12908–22. doi: 10.1002/ece3.7931 (PMC8495793; doi:10.1002/ece3.7931)

**The influence of phylogeny and life history on telomere lengths and telomere rate of change among bird species: a meta-analysis.**

Criscuolo F.^1‡^, Dobson F.S^1,2‡^ and Q. Schull ^3^

^1^ University of Strasbourg, CNRS, IPHC UMR 7178, 67000 Strasbourg, France.

^2^Auburn university, Department of Biological sciences, Auburn, AL, USA

^3^ MARBEC, University of Montpellier, IFREMER, IRD, CNRS, Sète, France

^‡^ Authors for correspondence ([francois.criscuolo@iphc.cnrs.fr](mailto:francois.criscuolo@iphc.cnrs.fr), [fsdobson@msn.com](mailto:fsdobson@msn.com))

**Electronic Supplementary Material 3: Cytochrome b sequences**

> Turdus merula cytochrome b gene, partial cds; mitochondrial

GTCACAGGCTTACTACTAGCTATACATTACACAGCAGACACCTCTCTAGCCTTTAACTCAGTCGCCCATA

TATGCCGAAACGTCCAATTCGGCTGACTAATCCGTAACCTCCATGCAAATGGAGCCTCTCTATTCTTTAT

CTGCATCTACCTCCACATCGGCCGAGGGTTTTACTACGGCTCATACCTCAACAAAGAGACTTGAAATATC

GGAGTTATTCTACTTCTAACCCTAATAGCAACAGCCTTCGTAGGCTACGTACTTCCTTGAGGACAAATAT

CATTCTGAGGGGCTACAGTAATCACCAACCTATTCTCAGCAATCCCCTACATTGGCCAAACACTAGTAGA

GTGAGCTTGAGGGGGATTCTCAGTAGACAATCCTACACTAACACGATTCTTCGCCCTCCATTTCCTCCTC

CCATTCGTTATCGCAGGACTCACGCTAGTGCACCTCACCTTCCTACACGAAACAGGATCAAACAACCCCC

TAGGAATCCCCGCAGACTGCGATAAAATCCCCTTTCATCCTTACTACTCTACAAAAGACATCCTAGGGTT

CGCACTAATGCTCATCCTCCTCGCCTCCCTAGCCCTATTTTCCCCCAATGCACTCGGAGACCCAGAAAAC

TTCACACCAGCCAACCCACTAACCACACCCCCGCACATCAAACCCGAATGATACTTCCTATTCGCATATG

CCATCCTCCGATCCATCCCAAACAAACTAGGAGGGGTGCTAGCACTAGCAGCCTCCGTCCTAGTCCTATT

CCTTACCCCACTCCTACACAAATCAAAACTACGCTCAATAACCTTCCGACCCCTATCGCAAATCCTATTT

TGAGCCCTAGTAGCCAACCTCCTAATCCTCACTTGAGTAGGGAGCCAACCAGTTGAACACCCATTCATTA

TCATCGGCCAACTAGCCTCACTCTCCTACTTCACAATCATTCTAGTCCTATTCCCCCTCGCAGCCGTACT

AGAAAATAAAA

> Gallus gallus jabouillei mitochondrial gene for cytochrome b, partial cds

ACACAGCAGACACTTCCCTAGCCTTCTCCTCCGTAGCCCACACTTGCCGGAACGTACAATACGGCTGACT

CATCCGGAATCTCCACGCAAACGGCGCCTCATTCTTCTTCATCTGTATCTTCCTTCACATCGGACGAGGC

CTATACTACGGCTCCTACCTCTACAAGGAAACCTGAAACACAGGAGTAATCCTCCTCCTCACACTCATAG

CCACCGCCTTTGTGGGCTATGTTCTCCCATGGGGCCAAATATCATTCTGAGGGGCCACCGTTATCACAAA

CCTATTCTCAGCAATTCCCTACATTGGACACACCCTAGTAGAGTGAGGCTGAGGGGGATTTTCAGTCGAC

AACCCAACCCTTACCGGATTTTTCGCCTTACACTTCCTCCTCCCCTTTGCAATCGCAGGTATTACTATCA

TCCACCTCACCTTCCTACACGAATCAGGCTCAAACAACCCCCTAGGCATCTCATCCGACTCTGACAAAAT

TCCATTTCACCCATACTACTCCTTCAAAGACATTCTGGGCTTAACTCTCATACTCACCCCATTCCTAACA

CTAGCCCTATTCTCCCCCAACCTCCTAGGAGACCCAGAAAACTTCACCCCAGCAAACCCACTAGTAACCC

CCCC

> jamaicensis Buteo cytochrome b (cytb) gene, partial cds; mitochondrial

TACTAACACAAATCATGACCGGCCTCCTACTAGCAACACATTACACCGCCGACACTACCCTAGCCTTCTC

GTCCGTAGCCCACACATGCCGAAACGTACAGTACGGCTGACTAATCCGTAACCTACACGCCAATGGAGCA

TCCTTCTTCTTCATTTGCATCTACCTACACATCGGTCGAGGACTATACTATGGCTCCTACCTATACAAAG

AAACTTGAAACACAGGAATTATCCTACTACTCACCCTCATAGCAACTGCCTTCGTAGGCTACGTACTCCC

ATGAGGCCAAATATCCTTCTGAGGAGCTACAGTCATCACCAATCTATTCTCAGCCATCCCTTACATTGGT

CAAACCATTGTAGAATGAGCCTGGGGAGGATTCTCCGTAGATAACCCTACCCTCACTCGATTCTTCGCCC

TACATTTCCTACTCCCATTCCTAATCGCAGGCCTCACCTTAATCCACCTTACCTTCCTCCACGAATCCGG

CTCAAACAACCCCCTAGGCATCATCTCAAACTGTGACAAAATCCCATTCCACCCCTACTTTTCCCTAAAA

GATATCCTAGGCTTCATACTAATGTTACTCCCACTAACAACTTTAGCCCTATTCTCACCTAACCTGCTAG

GAGACCCAGAAAACTTCACCCCAGCAAACCCCCTAACCACTCCTCCACATATCAAGCCAGAATGATATTT

TCTATTCGCATACGCCATTCTACGCTCAATCCCTAACAAATTAGGAGGAGTATTAGCCTTAGCCGCCTCC

GTCCTAATCCTATTCCTGATCCCCTTTCTCCACAAGTCCAAACAGCGCACAATAACCTTCCGACCCATCT

CCCAACTTCTATTCTGAACCCTAGTTGCCAACCTCCTCATTCTCACATGAATCGGTAGCCAACCAGTAGA

ACACCCATTCATCATCATTGGCCAACTAGCCTCCCTTACCTACTTTCTCATCCTTCTAGCCCTTTTCCCC

CTAACCGGAGCTCTAGAAAACAAGCTCCTAAACCACTAA

> Buteo swainsoni voucher UMMZ DM-11 cytochrome b (cytb) gene, partial cds; mitochondrial

TAACACAAATCATGACCGGCCTCCTACTAGCAACACACTACACCGCCGACACTACTCTAGCCTTCTCGTC

CGTAGCCCACACATGCCGAAACGTACAGTACGGCTGACTGATCCGCAACCTACATGCCAACGGAGCATCC

TTCTTCTTCATCTGCATCTACCTACACATCGGTCGAGGACTATACTATGGCTCCTACCTATACAAAGAAA

CTTGAAACACAGGGATTATCCTACTACTTACCCTCATAGCAACTGCCTTCGTAGGCTACGTACTCCCATG

AGGCCAAATATCCTTCTGAGGAGCTACAGTCATCACCAATCTATTCTCAGCCATCCCTTACATCGGACAA

ACCATTGTAGAATGGGCCTGAGGAGGGTTCTCCGTAGACAACCCTACCCTCACCCGATTCTTCGCCCTAC

ACTTCCTACTCCCATTCCTAATCGCAGGCCTCACCTTAATCCACCTTACCTTCCTCCACGAATCCGGCTC

AAACAACCCCCTAGGCATCATCTCAAACTGTGACAAAATCCCATTCCACCCCTACTTTTCTCTAAAAGAC

ATCCTAGGCTTCATACTAATACTACTCCCACTAACAACTTTAGCCCTATTCTCACCTAACCTGCTAGGAG

ACCCAGAAAACTTCACCCCAGCAAACCCCCTAACCACTCCACCACATATCAAACCAGAGTGATACTTCCT

ATTCGCATACGCTATTCTACGCTCAATCCCTAACAAATTAGGAGGAGTATTAGCCCTAGCCGCCTCCGTC

CTAATTCTATTCCTAATCCCCTTTCTCCACAAATCCAAACAACGCACAATAACCTTCCGACCCATCTCCC

AACTTCTGTTCTGAACCCTAGTTGCCAACCTCCTCATTCTCACATGGATCGGCAGCCAACCAGTAGAACA

CCCATTCATCATCATTGGCCAACTAGCCTCCCTCACCTACTTCCTCATCCTTCTAGCCCTTTTCCCCCTA

ACTGGAGCTCTAGAAAACAAACTCCTCAACCACTAA

> Meleagris gallopavo mitochondrion cytochrome b gene, complete cds

ATGGCACCCAATATCCGAAAATGACACCCCCTATTAAAAACAATCAACAACTCATTAATCGACCTCCCAA

CCCCATCCAACATCTCCGCTTGATGAAACTTCGGCTCCCTACTAGCAGTATGCCTCATCACTCAAATCTT

AACCGGCCTCCTACTAGCCATACATTACACTGCAGACACCACTCTTGCATTCTCTTCTGTGGCCTACACA

TGCCGAAACGTACAATACGGTTGACTCCTCCATAACCTCCATGCGAATGGGGCCTCATTCTTCTTCATCT

GCATCTTCCTACACATTGGACGCGGCCTATATTATGGTTCGTACCTATATAAAGAAACCTGAAATACAGG

AGTAGTCTTACTTCTCACCCTCATAGCAACAGCCTTTGTAGGCTATGTCCTTCCATGGGGACAAATATCA

TTCTGAGGGGCTACCGTCATCACAAACCTATTCTCAGCAATCCCCTACATCGGACAAACCCTAGTAGAGT

GAGCCTGAGGGGGATTCTCAGTAGACAACCCAACCCTTACCCGATTCTTCGCCCTCCACTTCCTCCTGCC

CTTCGTAATCGCAGGAATTACAATCATTCACCTCATATTTCTGCACGAATCAGGCTCAAACAATCCTCTT

GGCATCTCATCAAACGCTGACAAAATCCCATTCCACCCCTACTACTCTATCAAAGACATCCTAGGTCTAA

CAATCATACTAACCCCCTTACTCACATTAACCCTATTCTCACCTAACCTCTTAGGAGACCCAGAAAACTT

TACCCCAGCAAATCCACTAGTAACCCCCCCACACATTAAACCAGAGTGATACTTTCTATTTGCCTACGCA

ATCCTACGCTCAATCCCAAACAAACTTGGAGGTGTCCTAGCCTTAGCAGCATCAGTACTCATTCTTCTCC

TTATCCCCTTCCTTCATAAATCTAAACAACGGGCAATAACATTCCGGCCACTCTCACAAACCTTATTCTG

ACTCTTAGTAGCAAACCTCCTCATCCTAACCTGAGTAGGAAGCCAACCAGTAGAACACCCATTCATCATC

ATTGGCCAAATAGCATCCCTTTCCTACTTCACTATCTTACTAATCCTCTTCCCCTTAATCGGAGCCCTAG

AAAACAAAATACTCAACCTCTAA

> Chrysolophus pictus cytochrome b (cytb) gene, partial cds; mitochondrial

GGAGTCGTCCTACTCCTCACACTCATAGCAACCGCCTTTGTAGGGTACGTCCTCCCATGAGGACAAATAT

CATTTTGAGGAGCTACCGTCATCACAAACCTATTCTCAGCAATCCCTTACATCGGACAGACCCTAGTAGA

ATGAGCCTGGGGAGGATTCTCAGTTGACAACCCAACCCTCACCCGATTCTTCGCCCTACACTTCCTCCTT

CCCTTCATAATTGCAGGAATTACCATCACCCATCTCATATTTCTACACGAATCAGGCTCAAACAACCCCC

TAGGCATCTCATCTAACTCCGACAAAATCCCATTCCACCCATACTACTCCCTCAAAGATATCCTAGGCCT

AGCACTTATACTTACCCCATTTCTCACACTCGCCCTATTCTCCCCAAACCTTCTAGGCGACCCAGAAAAC

TTTACCCCAGCAAACCCATTA

> Colinus virginianus cytochrome b (cytb) gene, complete cds; mitochondrial

ATGGCCCCCAACATCCGAAAATCCCACCCATTACTAAAAATCATTAATAACTCCCTAATTGACCTCCCCA

CCCCATCCAATATCTCTGCCTGATGAAACTTCGGCTCCCTGCTAGCAATATGCCTAATAACCCAAATCAT

CACCGGACTACTCCTAGCCACACATTACACTGCAGACACAACCCTAGCTTTCTCTTCAGTCGCCCACACA

TGTCGAAACGTCCAATACGGATGACTCATCCGCAATCTACATGCAAACGGCGCCTCAATATTCTTCATCT

GCATCTACCTACACATTGGACGAGGAATCTACTATGGCTCCTACCTCTACAAAGAAACATGAAACACAGG

AGTAATCCTTCTTCTGACACTTATAGCAACCGCCTTCGTGGGCTACGTACTCCCATGAGGGCAAATATCC

TTCTGAGGAGCTACCGTCATCACCAACCTATTCTCAGCTATTCCGTACATTGGACAAACCCTGGTAGAAT

GGGCTTGAGGGGGATTCTCAGTCGACAACCCAACCCTAACCCGATTCTTCGCCCTAAACTTCCTCCTCCC

TTTTGCATTGGCAGGAATNACCATCATTCACCTAACATTTCTTTACGAATTAAGCTCAAACAAACCCCTA

GGAATTTCTTCAAACTCTGACAAAATCCCCTTCCACCCATACTACTCCCTTAAAGACATCCTAGGACTAA

CCCTCATAATCACCCCCCTACTCACACTAGCCCTATTCTCACCCAACCTATTAGGCGACCCAGAGAACTT

CACCCCAGCTAACCCCCTATCAACACCCCCCCACATCAAACCAGAATGATACTTCCTATTCGCCTATGCC

ATCCTCCGATCTATCCCAAACAAACTCGGAGGTGTACTAGCCTTAGCAGCTTCAGTACTTATCCTTCTTC

TAATTCCATTCCTACACAAATCCAAACAACGAACCATAACATTCCGCCCCCTATCCCAAATCCTATTCTG

ACTCCTAGCCGCCAACCTACTAATCCTCACCTGAGTCGGCAGCCAACCAGTAGAACACCCATTCATCATC

ATTGGTCAATTAGCATCATTCTCATACTTCACCACCCTCCTAATCCTCTTCCCCATTATCGGAACCCTAG

AAAACAAAATACTCAACTACTAA

> Haliaeetus leucocephalus cytochrome b (cytb) gene, partial cds; mitochondrial

TCTGCCTACTAACACAAATCCTGACCGGCCTCCTACTAGCAACACACTACACCGCAGATACCTCCCTAGC

CTTTTCATCCGTAGCCCATACATGTCGAAACGTACAGTATGGCTGACTAATCCGCAACCTACATGCCAAC

GGAGCATCTTTCTTCTTCATCTGCATCTACCTACACATTGGCCGAGGGCTATATTATGGCTCATACCTAT

ATAAAGAAACTTGAAACACAGGAATCATTCTATTACTCACCCTCATGGCAACTGCCTTCGTAGGCTACGT

ACTTCCATGAGGCCAGATATCCTTCTGAGGAGCCACAGTCATCACCAATTTATTCTCAGCCATCCCATAC

ATCGGACAAACCATCGTAGAATGAGCCTGAGGGGGATTTTCCGTAGACAACCCCACCCTCACTCGATTCT

TTGCCCTACACTTCCTACTCCCATTTCTAATCGCAGGCTTCACCCTAATCCACCTTACCTTCCTCCACGA

ATCCGGTTCAAACAACCCCCTAGGTATCATCTCAAACTGTGACAAAATTCCATTCCACCCATACTTCTCC

CTAAAAGATATCCTAGGATTTACACTAATGCTACTCCCACTAATAACCTTAGCCCTATTCTCACCTAACC

TGTTAGGAGACCCGGAAAACTTCACCCCAGCAAACCCCCTAGCCACACCACCCCATATCAAACCGGAATG

ATACTTCCTATTCGCATACGCCATCCTACGCTCAATCCCAAACAAACTAGGAGGAGTACTAGCCCTAGCT

GCCTCCGTACTAATTCTATTCCTAGTCCCCTTTCTTCACAAGTCCAAACAACGAACCATAACCTTTCGAC

CCTTCTCCCAACTACTATTCTGAACCCTAATCGCTAATCTCCTCATTCTCACGTGAATCGGAAGCCAACC

AGTAGAACACCCATTCATTATCATTGGCCAAGTAGCCTCCCTTACCTACTTCCTCATCCTTCTAGCCCTC

TTCCCCCTAACTGGAGCCCTAGAAAACAAACTTCTCAACCACTAA

> Falco sparverius cytochrome b (cytb) gene, mitochondrial gene encoding mitochondrial protein, complete cds

ATGGCACCCAACATTCGAAAATCACACCCCCTAATAAAAATAATCAACAACTCCCTAATTGACCTCCCCA

CCCCACCCAACATCTCCATATGATGAAACTTCGGATCCTTACTAGGAGTCTGTCTAGCCACCCAAATCCT

AACTGGCCTACTACTAGCCATACACTACACAGCAGACACAACACTGGCCTTCTCATCTGTTGCCCACACA

TGCCGAAACGTGCAGTACGGATGGTTGATCCGTAACCTACATGCCAACGGAGCATCACTATTCTTCATCT

GCATTTACATACACATCGGACGAGGAATCTACTACGGCTCTTATTTATACAAAGAAACCTGAAACACAGG

AATCATCCTCCTACTCACCCTAATAGCCACAGCCTTCGTCGGCTATGTGTTACCCTGAGGACAAATGTCA

TTCTGAGGAGCCACAGTCATTACCAACCTATTCTCAGCAATCCCATATATCGGCCAAACCCTAGTCGAAT

GGGCCTGAGGAGGATTCTCAGTAGACAACCCAACACTAACCCGCTTCTTCGCCTTACACTTCCTCCTACC

ATTCCTAATCGCAGGGCTTACCTTAATCCACCTCACCTTCCTACATGAATCAGGTTCCAACAACCCCCTA

GGAGTCACATCAAACTGTGACAAAATCCCATTCCACCCCTACTACTCTCTCAAAGACCTCCTAGGTTTTA

TGCTCATACTCCTGCCCCTAATAGCCCTAGCCCTATTCACCCCAAACCTGCTAGGAGACCCAGAAAACTT

CACACCAGCGAACCCCCTAGTCACCCCACCACACATCAAACCAGAATGATACTTCCTATTTGCCTACGCT

ATTCTACGCTCAATTCCCAACAAATTAGGCGGAGTCTTAGCACTAGCCGCCTCCGTATTAATCCTGTTCC

TTAGCCCGCTACTACATAAATCCAAACAACGTACAATAACCTTCCGCCCTCTATCCCAATCACTATTCTG

ACTCCTAGTCACCAACCTACTCATCCTAACCTGAGTAGGAAGCCAACCCGTTGAACACCCATTCATTATC

ATCGGCCAACTTGCCTCCCTCTCCTACTTTACAATTCTCCTAATTCTCTTTCCCCTCGCTGGAGCCTTAG

AAAACAAAATCCTCAACTACTAA

> Amazona amazonica voucher USNM B12700 cytochrome b (CYTB) gene, partial cds; mitochondrial

ACACAAATTCTAACAGGCTTACTCCTAGCCGCCCACTACACCGCAGACACCTCTCTAGCTTTCTCATCCG

TAGCCAACACATGCCGAAACGTACAGTACGGCTGACTAATCCGCAACCTTCACGCAAATGGAGCCTCACT

CTTCTTCATCTGCATCTACCTGCATATCGCCCGAGGCTTCTACTACGGCTCATACCTGTATAAAGAAACC

TGAAACACAGGAGTCGTCCTTCTCCTCACCCTCATAGCAACAGCCTTCGTGGGATACGTCTTACCATGAG

GCCAAATATCATTCTGGGGGGCTACAGTCATCACAAACCTATTCTCTGCCATCCCCTACATCGGACAAAC

CCTAGTAGAATGAGCCTGAGGGGGATTCTCCGTAGACAACCCCACCTTAACCCGATTTTTCGCTCTACAC

TTCCTCCTCCCATTCATGATCACCAGCCTAGTCCTTATCCACCTAACCTTCCTACACGAGTCGGGGTCAA

ATAACCCCCTAGGCATTCCATCAAACTGTGACAAAATCCCATTCCATCCCTACTTCTCCCTAAAAGACCT

TCTAGGATTCATAATCATACTTCTCCTACTCTCCACCCTCGCTCTATTCTCCCCTAACCTACTAGGGGAT

CCCGAAAACTTCACCCCAGCAAATCCCCTAGTAACTCCCCCACACATCAAACCAGAATGATACTTCCTAT

TCGCATATGCAATCCTACGCTCAATTCCCAACAAACTGGGAGGAGTCCTAGCCCTAACTGCCTCCGTACT

AATCCTGCTTCTAAGCCCCCTTCTCCACAAATCCAAACAACGAACCATGGCCTTCCGCCCCATCTCCCAA

CTCCTATTCTGAGCCCTAGCCGCCAACC

> Grus antigone cytochrome b (cytb) gene, partial cds; mitochondrial

CTCTTCTCAGCCGTCCCCTACATCGGCCAAACCCTTGTAGAATGAGCTTGAGGGGGCTTCTCAGTAGACA

ATCCCACATTAACTCGATTCTTCACTTTACACTTCCTCCTTCCATTCATAATCATAGGCCTCACCCTAAT

CCACCTCACCTTCCTTCACGAATCCGGCTCAAACAACCCCCTAGGCATCGTATCAAACTGCGATAAAATC

CCATTCCACCCCTACTTTTCCTTAAAAGATATCCTAGGATTCACACTCATACTACTTCCACTCATAACCC

TAGCCCTATTCTCACCAAACCTACTAGGAGACCCAGAAAACTTCACCCCAGCAAACCCCCTAGTCACACC

TCCTCATATCAAGCCAGAATGATACTTTTTATTTGCATACGCCATCCTACGATCAATCCCTAACAAACTA

GGAGG

> Accipiter gentilis mitochondrial cytb gene

GGCTCCCTCCTAGGAATCTGTCTACTAACACAAATCCTAACCGGCCTACTACTAGCAACACACTACACCG

CGGACACCACCCTAGCCTTCTCATCCGTAGCCCATACATGCCGAAACGTACAATACGGCTGACTAATCCG

CAATCTGCATGCTAACGGAGCATCCTTCTTCTTCATTTGCATCTACTTCCACATCGGCCGAGGACTTTAC

TATGGCTCATACCTCTACAAAGAAACCTGAAACACAGGCATCATTCTCCTTCTAACCCTCATAGCAACCG

CCTTCGTAGGCTACGTNCTCCCATGAGGCCAAATATCCTTCTGAGGAGCTACAGTNATCACCAATCTATT

CTCAGCCATCCCCTACATTGGACAAACCATCGTAGAGTGAGCCTGAGGAGGATTCTCCGTAGACAACCCC

ACCCTCACCCGATTCTTCGCCCTACACTTCCTACTCCCATTCCTAATCGCAAGCCTCACCCTAATTCACC

TTACCTTCCTCCACGAATCAGGCTCAAACAACCCCCTAGGTATCATTTCAAACTGCGACAAAATCCCCTT

CCACCCGTACTTTTCCCTAAAAGACNNCTTAGGCTTCCTACTAATATTACTCCCACTAACAACCCTAGCA

CTATTCTCCCCCAACCTTCTAGGAGACCCAGAAAATTTCACCCCTGCAAACCCCCTAACTACACCTCCAC

ATATTAAGCCAGAATGATATTTCCTATTCGCATACGCTATCCTACGCTCAATCCCAAACAAACTAGGAGG

AGTACTAGCCCTAGCCGCCTCTGTATTAATTCTGTTCCTAATCCCCTTTCTACATAAATCCAAACAACGC

ACAATAACCTTTCGGCCCTTCTCCCAAATTCTATTCTGAACCCTAATTGCCAACCTCCTAATCCTCACAT

GAATCGGTAGTCAACCAGTAGAACACCCCTTTATTATTATTGGCCAACTAGCTTCCCTTACATACTTCTC

CATCCTCTTAGCCCTCTTCCCACTAACCGGAGCCCTAGAAAACAAA

> coerulescens Aphelocoma cytochrome-b gene, mitochondrial gene encoding mitochondrial protein, complete cds

ATGGCCCTAAATCTACGCAAAACCCACCCCCTACTAAAAATCGTCAATGACTCCTTAATTGACCTTCCCA

CTCCATCAAACATTTCAGCTTGATGAAACTTCGGATCTCTACTAGGCATCTGCCTAATCACACAAATCAT

CACAGGTCTACTGCTAGCCATACACTACACAGCAGATACCTCCCTTGCTTTTACATCCGTAGCCCACATG

TGCCGAAACGTACAATTCGGATGACTAATCCGCCACCTACATGCAAACGGAGCTTCCTTCTTCTTCATCT

GTATTTACTTACATATCGGCCGAGGATTCTATTATGGTTCCTACCTAAACAAAGAAACCTGAAACATCGG

AGTAATCCTCCTACTAACCCTGATAGCAACTGCTTTCGTAGGATACGTCCTGCCCTGAGGACAAATATCT

TTCTGAGGTGCCACAGTCATCACCAACCTCTTTTCAGCAATTCCATACGTCGGACAAACACTAGTAGAAT

GACTCTGAGGAGGATTTTCAGTAGACAACCCTACACTAACTCCCCTCTTTGCCCTCCACTTTCTACTTCC

CTTTGTAATCGCAGGCCTAACACTAGTCCATCTAACCTTCCTGCATGAAACCGGATCAAACAATCCACTG

GGAATCCCCTCAGACTGCGATAAAATCCCATTCCAGCCCTACTACTCCATTAAAGACCTACTAGGATTTG

TACTAATACTAACACCACTCATTGCTATAGCACTATTCGCCCCAAACTTCCTAGGAGACCCTGAAAATTT

CACGCCCGCCAACCCCCTAGTTACACCCCCACATATCAAACCAGAATGATATTTCCTATTTGCATATGCC

ATTCTACGATCCATTCCAAACAAATTAGGAGGAGTCCTAGCCCTAGCTGCCTCAGTCCTAGTCCTATTCC

TTATCCCCCTACTACACATCACCAAACAACGCTCAATAACTTTCCGCCCTCTATCCCAAATGTTATTTTG

AACCCTAGTCGCAGATCTACTCATCCTAACATGAGTCGGAAGCCAACCAGTCGAACATCCATTCATCATT

ATTGGTCAACTAGCCTCCCTTGCCTACTTCACAATCATTCTAGTCTTATTCCCCATCGTAAGCGCACTAG

AAAACAAAATACTCAACCTCTAA

> Aphelocoma ultramarina ultramarina isolate MEJA3 cytochrome b (CYTB) gene, partial cds; mitochondrial

TTATTACAGGCCTACTGCTAGCTATACACTATACAGCAGATACCTCCCTCGCTTTTACATCTGTAGCCCA

CATATGCCGAAACGTCCAATTCGGATGACTGATCCGCAACCTACATGCAAACGGAGCTTCATTCTTCTTC

ATCTGCATTTACCTACACATCGGCCGAGGATTCTACTATGGTTCCTACCTAAACAAAGAAACCTGAAACA

TTGGAGTAATCCTTCTACTAACCTTAATAGCAACCGCTTTCGTAGGATATGTCCTACCCTGAGGACAAAT

ATCCTTCTGAGGCGCTACAGTCATCACCAACCTCTTCTCAGCAATCCCATACGTCGGACAGACATTAGTA

GAATGACTCTGAGGAGGATTTTCAGTAGACAACCCTACATTAACCCGATTTTTTGCCTTCCACTTCCTTC

TTCCCTTCGTAATCGCAGGCTTAACACTAGTCCATCTAACCTTCCTGCACGAAACCGGATCAAATAACCC

ACTAGGAATCCCCTCAGACTGCGATAAAATTCCATTCCACCCCTACTACTCCATCAAAGACCTATTAGGA

TTCGCACTAATACTAATCCCACTTATCACCCTGGCACTATTTGCCCCAAACTTCCTAGGAGACCCCGAAA

ATTTCACGCCCGCCAACCCTCTGGCTACACCCCCTCATATCAAACCAGAATGATACTTCCTATTTGCATA

TGCCATCCTCCGATCTATCCCAAATAAATTAGGAGGTGTCCTGGCCCTGGCTGCCTCAGTCCTAGTTCTA

TTCCTCATTCCCCTATTACACGTCTCCAAACAACGATCAATAACTTTCCG

> Cepphus grylle cytochrome b gene, mitochondrial gene encoding mitochondrial protein, partial cds

CTTTGGATCCCTACTAGGCATTTGCCTACTAACACAAATCCTCACAGGACTTCTACTAGCCACACATTAC

ACCGCAGACACAACCCTAGCCTTCTCATCCGTTGCCCACACCTGCCGAAATGTTCAATATGGCTGACTAA

TCCGAAACCTCCATGCAAACGGAGCATCATTCTTCTTCATCTGCATCTACCTCCACATTGGACGAGGATT

CTACTACGGCTCATACTTAAACAAAGAAACCTGAAACACAGGCGTCATCCTCCTTTTAACCCTAATAGCA

ACAGCCTTCGTAGGATATGTCCTACCATGAGGCCAAATATCATTCTGAGGAGCTACAGTCATCACTAACC

TATTCTCAGCAATCCCATACATCGGCCAAACTCTTGTAGAATGAGCATGAGGTGGTTTCTCAGTAGACAA

CCCAACACTAACCCGATTCTTTGCACTTCACTTCCTTCTCCCATTCATAATTGCAGGTCTCGCCCTAATC

CACCTTACCTTCCTCCACGAATCTGGATCAAACAACCCACTAGGAATCCTATCAAACTGTGACAAAATCC

CATTCCATCCCTACTTCTCGCTAAAAGACATCCTAGGATTCATCATCATATTCCTTCCACTAACAACCCT

AGCCCTATTCTCACCCAATCTACTAGGCGACCCAGAAAACTTCACCCCCGCAAACCCCCTAATTACACCT

CCACATATCAAACCAGAATGATATTTCCTATTTGCATACGCCATCCTACGTTCAATCCCCAATAAACTAG

GAGGAGTATTAGCCCTAGCAGCCTCCGTACTAGTACTATTCTTAACACCTCTGCTCCACAAATCCAAACA

ACGCGCAATAACCTTCCGTCCCCTCTCACAACTCTTATTCTGAACTCTAGTCACCAACCTCTGTATTCTC

ACATGAGTAGGCAGCCAACCCGTAGAACACCCATTCATCATCATCGGACAACTAGCCTCCCTCACTTACT

TCACTATCCTACTGCTCCTATTCCCTATCATCGGAGCCCTAGAAAACAAAATACTCAACTACTAA

> Fulmarus glacialis partial mitochondrial cytb gene, isolate 1

TTCGGATCCCTCTTAGGTATCTGCCTATTAACCCAAATCCTAACCGGTCTATTACTAGCCATACACTACA

CTGCTGACACAACCTTAGCCTTCTCATCCGTTGCCCACACATGCCGAAACGTACAGTACGGCTGACTCAT

TCGAAATCTACATGCAAACGGAGCCTCATTCTTTTTCATCTGCATTTACCTACATATTGGACGAGGATTC

TACTATGGCTCCTACCTTTACAAAGAAACCTGAAACACAGGAATTATCCTTCTACTCACCCTCATAGCAA

CCGCCTTCGTAGGATATGTCCTACCTTGAGGCCAAATATCATTCTGAGGGGCCACAGTAATCACTAATCT

ATTCTCAGCCATTCCCTATATTGGCCAAACCCTCGTAGAATGAGCCTGAGGAGGATTTTCGGTAGATAAT

CCCACACTAACCCGATTCTTTGCCCTACACTTCCTCCTTCCCTTTGCAATTGCAGGGCTTACCCTAATCC

ACCTTACCTTCCTTCACGAATCAGGCTCAAACAACCCCCTAGGCATCGTATCAAACTGTGACAAAATCCC

ATTCCACCCCTACTTCACCCTAAAAGACATCCTAGGTTTCACACTCATATTCCTTCCACTAACATCCCTA

GCCCTATTCTCCCCCAATCTACTGGGAGACCCAGAAAACTTTACTCCCGCAAACCCACTAGTCACACCTC

CCCATATCAAACCAGAGTGGTACTTCTTATTCGCATATGCCATCCTACGCTCAATCCCCAACAAATTAGG

CGGAGTATTAGCTTTAGCTGCCTCCGTACTAGTCATATTCCTATCCCCATTTCTCCATAAAGCCAAACAA

CGAACAATAACCTTCCGCCCCCTCTCCCAACTACTATTTTGAATCCTAGTCACTAACCTATTCATCCTAA

CATGAGTAGGAAGCCAACCAGTAGAACATCCATTCATCATCATCGGCCAACTAGCCTCCATCACCTACTT

TACTATCCTCCTAATCCTCTTCCCTATCATCGGAGCCCTAGAAAACAAAATACTTAACTTC

> Haematopus ostralegus cytochrome b (cytb) gene, complete cds; mitochondrial

ATGGCCCCAAACCTACGTAAATCCCACCCCCTACTCAAAATAATCAACAGCTCCCTAATCGACCTACCTA

CACCCCCAAACATCTCTGCTTGATGAAACTTTGGATCACTACTAGGCATCTGCTTAATGACACAAATCCT

AACAGGCCTATTACTAGCCATACACTACACCGCAGATACAACCCTAGCCTTCTCCTCCGTTGCTCACACA

TGCCGAAACGTCCAATACGGGTGACTAATCCGCAACCTACATGCAAACGGAGCCTCATTCTTCTTCATCT

GCATCTACCTACACATCGGACGAGGCTTCTACTATGGCTCGTACCTATACAAAGAAACCTGAAATACAGG

TGTAATCCTCCTCCTGACCCTAATAGCCACCGCCTTCGTAGGCTATGTCCTACCATGAGGGCAAATATCA

TTCTGAGGGGCTACAGTCATCACCAACCTATTCTCAGCTATTCCATACATCGGGCAAACCCTTGTAGAAT

GGGCATGAGGAGGATTCTTCGTAGACAACCCAACACTGACCCGATTCTTTGCCCTACACTTCCTCCTCCC

ATTCTTAATCGCAGGCCTTACCCTAATCCACCTCACCTTCCTCCATGAATCGGGCTCAAATAACCCCTTG

GGCATCGTATCAAACTGTGACAAAATTCCATTCCACCCATACTTCTCCATAAAAGATATCCTAGGGTTCA

TCCTAATACTCCTCCCACTAATAACCCTAGCTATATTCTCACCCAACCTGCTAGGAGACCCAGAAAACTT

CACGCCAGCAAACCCACTAGTTACACCCCCCCACATCAAACCAGAATGATACTTCCTATTTGCATATGCA

ATCTTACGCTCAATCCCTAACAAACTAGGAGGTGTACTAGCACTAGCCGCCTCAGTACTAGTTCTCTTCC

TCACCCCTCTCCTCCATAAGTCCAAACAACGCACAATAGCATTCCGTCCCATCTCACAGCTACTATTCTG

AATCCTGGTCGCCAACCTCATCATCCTAACATGAGTAGGCAGCCAACCAGTAGAACACCCATTCATCATC

ATTGGCCAACTAGCCTCCATCACCTACTTCACCATCCTCCTACTCCTCTTTCCCATCACTGGAGCCCTAG

AGAACAAAATACTCAACTACTAA

> Lonchura striata cytochrome b (cytb) gene, partial cds; mitochondrial

ATTTGTCTAATTACCCAAATTGTAACAGGCCTGCTGCTAGCAACACACTACACAGCAGACACCTCCCTAG

CTTTCGCCTCAGTAGCCCACATATGCCGAGATGTACAATTTGGCTGACTAATCCGAAACCTCCACGCAAA

CGGCGCCTCCTTCTTCTTCATCTGCATCTACCTTCACATCGGCCGAGGAATCTACTACGGCTCATATCTA

AACAAAGAGACCTGAAACATCGGAGTCATTCTCCTCCTAACCCTCATAGCAACTGCCTTCGTAGGATATG

TACTACCCTGAGGACAAATATCCTTCTGAGGAGCCACAGTAATCACAAACCTACTCTCAGCCATCCCCTA

CATCGGCCAAACATTAGTAGAATGAGCCTGAGGAGGATTCTCAGTAGACAACCCTACACTAACACGATTC

TTCGCCCTCCACTTCCTACTTCCATTCGTCATCGCAGGACTCACACTAGTACACCTCACCTTCCTGCACG

AAACAGGCTCAAACAACCCATTAGGAATCCCATCAGACTGTGACAAAATCCCCTTCCACCCATACTACAC

CACAAAAGACATCCTAGGCTTCGCACTAATACTCTCCCTGCTAGCCTCACTAGCTTTATTCTCCCCCAAC

CTACTAGGAGACCCAGAAAACTTCACCCCCGCCAACCCCCTAGTAACACCTCCACACATCAAACCTGAAT

GATACTTCCTATTCGCCTACGCCATTCTACGATCCATCCCCAACAAACTAGGAGGAGTCCTAGCCCTAAT

AGCTTCAATCCTAGTTCTATTCCTACTTCCCCTACTCCACACATCCAAACTACGATCAATAACATTCCGA

CCACTCTCCCAAATCCTATTCTGAACCCTAGTCGCCAACGTCCTAGTTCTCACATGAGTAGGCAGCCAAC

CAGTAGAACACCCATTCATTATCATTGGCCAACTAGCCTCACTCTCATACTTCACAATTATCCTAATTTT

ATTCCCCCTCACAGCCATCCTAGAGAATAAAATACTGAAACTATAA

> Parus major cytochrome b (cytb) gene, complete cds; mitochondrial

ATGGCTCCCAACCTTCGTAAAAACCACCCCCTACTAAAAATCATCAACGACTCCCTAATCGACCTCCCCA

CTCCTTCCAACATTTCCGCCTGATGGAACTTCGGATCACTCCTAGGCATCTGCCTAGTAACTCAAATCAT

CACCGGCCTACTACTAGCCATACACTACACAGCAGACACCACACTAGCCTTCTCCTCCGTAGCCCACACC

TGCCGAAACGTCCAATTCGGCTGACTCATCCGAAACCTCCACGCAAACGGAGCCTCCTTCTTCTTCATCT

GCATCTACTTCCACATCGGTCGAGGAATCTACTACGGGTCCTACCTAAACAAAGAAACCTGAAACATCGG

AGTTATCCTACTCCTAGCCCTCATAGCAACCGCCTTCGTAGGATATGTACTACCTTGAGGACAAATATCC

TTCTGAGGCGCCACAGTAATCACAAACCTATTCTCAGCAATCCCGTACATCGGCCAAACACTAGTTGAAT

GAGCCTGAGGCGGCTTCTCAGTAGACAACCCAACCCTAACCCGATTCTTCGCCCTACACTTCCTGCTACC

CTTTGTTATCGCAGGCCTAACACTAGTCCACCTCACCTTCCTCCACGAAACAGGATCCAACAACCCCCTA

GGAATCCCCTCAGACTGTGACAAAATCCCATTCCACCCATACTACTCCACAAAAGACATCCTAGGCTTTG

CACTAATACTCATCCTCCTTGTAACCCTAGCCCTATTCTCACCCAACCTCCTAGGAGATCCAGAAAACTT

CACCCCAGCAAACCCCCTATCCACCCCCCCTCACATTAAACCCGAATGATACTTGCTATTTGCCTATGCC

ATCCTCCGCTCCATTCCCAACAAACTAGGAGGAGTCCTCGCCCTAGCTGCCTCCGTCCTAGTTCTATTCC

TAATACCCCTACTTCACACATCTAAACAACGTTCAATAACCTTCCGCCCCCTGTCCCAAATCCTATTCTG

AACCCTAGTCGCCAACCTCCTCGTTCTAACCTGAGTAGGGAGCCAACCAGTCGAACATTCCTTCATCATC

ATCGGGCAACTAGCCTCCCTATCATACTTCACAATTATCCTAGTCCTATTCCCCCTTGCTGCTATACTAA

AAAACAAGATCCTCAAACTTTAA

> Passerculus sandwichensis cytochrome b (cytb) gene, complete cds; mitochondrial

ATGGCCCCCAACCTTCGTAAAAACCACCAAATCCTCAAAATCATCAATAACGCCCTCATCGATCTCCCGA

CACCATCAAACATCTCAGTATGATGAAACTTCGGGTCACTACTGGGCGTCTGCCTAATTACTCAAATCAT

TACAGGTCTCCTGCTAGCCATGCATTACACAGCAGACACTAGCCTAGCCTTCTCCTCCGTCGCCCACATA

TGCCGAGACGTACAATTCGGCTGACTCATCCGCAACCTCCACGCAAACGGAGCCTCCTTCTTCTTCATCT

GCATCTACCTACATATCGGCCGAGGCATCTACTACGGTTCATACTTAAACAAAGAAACCTGAAACATCGG

AATTATCCTTCTCCTGGCCCTCATAGCAACCGCTTTCGTAGGATACGTCCTCCCATGAGGCCAAATATCC

TTCTGAGGAGCCACCGTCATCACAAACCTATTCTCAGCCATCCCCTACATCGGACAAACACTAGTCGAAT

GAGCCTGAGGCGGATTCTCCGTTGACAACCCCACACTCACCCGATTCTTCGCCCTCCACTTCCTCCTCCC

CTTCCTGATCGTAGGCATCACCCTCGTCCATCTCACATTCCTCCACGAAACAGGCTCAAACAACCCACTA

GGCATCCCCCCAGACTGCGACAAAATCCCTTTCCACCCATACTACACTATCAAAGACATCCTAGGGTTCG

TACTAATACTCTCCCTACTAGTCGCACTAGCCCTATTCTCTCCCAACCTCCTAGGCGACCCAGAAAACTT

CACCCCCGCCAACCCACTAGTCACCCCTCCCCACATCAAACCCGAATGATACTTCCTATTTGCCTACGCC

ATCCTCCGATCCATCCCGAACAAACTAGGAGGCGTACTAGCCCTAGCCGCCTCAATCCTTGTCCTATTCC

TCCTTCCACTGCTACATACATCAAAACTACGATCAATAACTTTCCGTCCCCTTTCACAAATCCTATTCTG

AACCCTAGTCGCCAACGTCCTCATCCTCACTTGAGTAGGCAGCCAACCAGTAGAACACCCCTTCATCATC

ATCGGCCAGCTAGCCTCATTCACGTACTTCACAATCATTCTAGTCCTATTCCCCCTTGCGGCCCTCCTAG

AAAACAAGATACTTAAACTCTAA

> Philomachus pugnax cytochrome b (cytb) gene, partial cds; mitochondrial

TTCGGGTCACTTTTAGGCATCTGCCTCGCAACGCAAATCCTAACCGGTCTCCTACTCGCCATGCACTATA

CCGCAGACACAACTCTAGCCTTCTCATCCGTCGCCCACACATGTCGAAACGTACAGTATGGCTGACTTAT

TCGTAACTTACATGCAAACGGAGCCTCATTCTTTTTTATTTGCATCTACCTCCACATCGGACGAGGCTTT

TACTACGGCTCATACCTGTATAAAGAAACATGAAACACAGGAGTCATCTTACTCCTAACCCTTATAGCAA

CTGCTTTCGTAGGATATGTTCTCCCATGAGGACAAATATCCTTCTGAGGGGCTACAGTCATCACCAATTT

ATTCTCAGCAATCCCCTACATCGGCCAAACCCTAGTAGAATGGGCATGAGGTGGCTTCTCAGTAGATAAC

CCAACACTAACTCGATTCTTCGCCCTCCACTTCCTACTTCCATTCATAATCGCAGGCCTCACCCTAATCC

ACCTCACCTTCCTACACGAGACCGGCTCAAATAATCCACTAGGTATTGTATCAAACTGCGACAAAATCCC

ATTCCACCCTTACTTCTCACTCAAAGACATCCTAGGGTTCATTATTATATTCCTGTTCCTACTAACTCTC

GCCCTATTCTCCCCCAACCTACTAGGAGACCCAGAAAACTTCACTCCAGCGAACCCCCTAGTCACACCCC

CACACATTAAACCCGAATGATACTTCCTGTTCGCATATGCTATCCTACGCTCAATCCCAAACAAACTGGG

AGGAGTACTAGCTCTGGCAGCCTCCGTATTAGTTCTATTCCTAACCCCACTCCTCCACAAATCCAAACAA

CGTTCAATAACCTTCCGCCCTTTATCCCAACTCCTATTCTGAACCCTAGTCGCCAATCTCTTCATCCTAA

CCTGAGTAGGTAGCCAACCTGTAGAACACCCATTCATCATCATTGGACAACTAGCATCCCTTACTTACTT

TACAATCCTCCTAATCCTATTCCCC

> Rissa tridactyla voucher AJB5531 cytochrome b (cytb) gene, complete cds; mitochondrial

ATGGCCCCAAATCTACGAAAATCCCACCCTCTCCTCAAAATAGTTAACAACTCACTAATCGACCTCCCCA

CCCCACCAAACATCTCTACTTGATGAAACTTCGGATCCCTACTAGGCATCTGTCTACTGACACAAATCCT

AACAGGACTTCTGCTAGCCATACACTATACCGCAGATACAACCCTAGCTTTCTCGTCCGTCGCCCACACA

TGTCGAAATGTACAATACGGCTGACTAATCCGAAACCTCCACGCAAACGGAGCATCATTCTTCTTTATCT

GTATCTACTTACACATTGGACGAGGATTCTACTACGGCTCCTACCTCTACAAAGAAACCTGAAACACAGG

AGTTATTCTCCTCCTAACCCTAATAGCAACTGCCTTCGTAGGATATGTCCTACCATGAGGACAAATATCT

TTCTGAGGTGCTACAGTCATTACTAACCTATTCTCAGCAATTCCATATATCGGCCAAACTCTCGTAGAGT

GAGCATGAGGTGGCTTCTCAGTGGACAACCCAACACTAACCCGATTCTTTGCCCTCCACTTCCTACTTCC

ATTCATAATCGCAGGTCTTACCTTTATCCACCTAACCTTTCTTCACGAATCGGGATCAAACAATCCACTG

GGTATTCAATCAAACTGTGATAAAATCCCATTTCACCCTTACTTCTCACTCAAAGACATCCTAGGATTCA

TCATCATATTCCTGCCACTAACAACATTAGCCCTATTCTCCCCAAACCTACTAGGCGACCCAGAAAACTT

TACTCCAGCAAACCCACTCGTTACACCCCCGCATATCAAACCCGAATGATACTTCCTGTTCGCATACGCT

ATCCTACGCTCAATCCCCAACAAACTAGGAGGCGTATTAGCCCTTGCAGCATCCGTACTAGTACTATTCC

TATGCCCCCTACTCCACAAATCCAAGCAACGTACAATAACCTTCCGCCCCCTCTCACAACTCTTATTCTG

AATCCTAGTTGCTAACCTCTTCATCCTGACATGAGTAGGCAGTCAACCCGTAGAACACCCCTTCATCATT

ATTGGACAACTAGCCTCCCTAACCTACTTCACCATCCTACTACTCCTATTCCCCGCTATCGGAGCCTTAG

AAAACAAAATACTTAACTACTAA

> Phalacrocorax aristotelis partial mitochondrial cytb gene, isolate 1

TTCGGATTCCTCCTAGGCATCTGCCTAATAACACAAATCTTAACCGGCCTATTACTAGCCATGCATTACA

CTGCAGACACACCCTTAGCATTCTCATCCGTCTCCCACACCTGCCGAAACGTCCAATATGGCTGACTAAT

CCGTAACCTACACGCAAACGGCGCNTCATTCTTCTTCATCTGCATCTACTTCCACATCGGCCGAGGACTC

TACTTTGGCTCATACCTATACAAAGAAACATGAAACACAGGAATTATTCTACTACTAACCCTCATAGCAA

CCGCTTTCGTAGGATATGTCCTACCTTGAGGACAAATATCCTTCTGAGGCGCTACAGTAATCACCAACCT

ATTCTCAGCCATCCCATACATCGGCCAAACCCTCGTTGAATGAGCTTGAGGAGGATTCTCAGTAGACAAC

CCAACACTAACACGCTTCTTCGCCCTACACTTCCTACTTCCCTTTATAATCGCAGGCCTAACCACAATCC

ACCTCACCTTCCTGCACGAATCTGGTTCCAACAACCCACTAGGCATTTCATCAAACTGCGATAAAATCCC

ATTCCACCCATATTTCTCCATAAAAGATATTCTAGGCTTCACACTCCTACTCCTCCTCCTAACAACAATA

GCCTTATTTTCCCCAAACCTCTTAGGAGACCCAGAAAACTTCACCCCAGCAAACCCACTAGCCACCCCAC

CTCACATTAAACCAGAATGATACTTCCTATTTGCATACGCTATTCTACGCTCAATCCCCAACAAACTAGG

AGGAGTACTAGCCCTGGCTGCCTCCGTCCTAATCCTCTTCCTAACCCCCCTCCTCCATAAATCCAAACAA

CGCTCAATAACATTTCGTCCACTCTCTCAAATCCTGTTCTGAGCACTAGTGGCCAACTTATTTATCCTAA

CATGAATTGGCAGCCAACCAGTAGAACACCCATTCATCATCATCGGCCAACTGGCNTCCATAACCTATTT

CACCATCCTACTAATCCTCTTTCCCCTTATCGGAGCCCTAGAAAACAAAATACTCAACTAC

> Larus fuscus mitochondrial cytb gene for cytochrome b, isolate DMM_0144

ATGGCCCCAAATCTACGAAAATCCCACCCCCTCCTCAAAATAGTTAACAACTCACTAATCGACCTCCCCA

CCCCACCAAACATCTCTACTTGATGAAACTTCGGATCCCTACTAGGCATTTGCCTACTAACACAAATCCT

AACAGGACTCCTGCTAGCTATACATTACACCGCAGACACAACCCTAGCCTTCTCATCCGTCGCCCACACA

TGTCGAAACGTACAATATGGCTGACTAATCCGAAACCTCCACGCAAACGGAGCATCATTCTTCTTTATTT

GTATTTACCTACACATCGGACGAGGATTCTACTATGGCTCATACCTCTATAAAGAAACCTGAAATACAGG

AGTCATTCTCCTCTTAACCCTAATAGCAACTGCCTTCGTAGGGTATGTCTTGCCATGAGGACAAATATCC

TTCTGAGGTGCTACAGTCATCACCAACCTATTCTCAGCAATCCCATACATCGGCCAAACCCTCGTAGAAT

GAGCCTGAGGTGGCTTCTCAGTGGACAACCCAACACTAACCCGATTCTTCGCCCTCCATTTCCTACTTCC

ATTCATAATCGCAGGTCTTACCTTCATCCACCTAACCTTCCTTCACGAATCAGGATCAAACAATCCACTG

GGTATTCAATCAAACTGTGACAAAATCCCATTCCACCCCTACTTCTCGCTCAAAGACATCCTAGAATTCA

TTATCATATTCTTACCACTAACAACATTAGCTCTATTCTCACCCAACCTGTTAGGTGATCCAGAAAACTT

TACTCCAGCAAACCCACTCGTTACACCCCCACACATCAAACCCGAATGATACTTCCTATTCGCATACGCT

ATCTTACGCTCAATCCCCAACAAACTAGGAGGCGTATTAGCCCTTGCAGCATCCGTACTAGTACTATTCT

TAAGCCCCCTACTCCACAAATCTAAGCAGCGTACAATAACCTTCCGTCCTCTCTCACAACTCCTATTCTG

AATCCTAGTTACTAACCTCTTCATCCTAACATGAGTAGGCAGTCAACCCGTAGAACATCCCTTTATCATC

ATTGGACAACTGGCTTCCTTAACCTACTTCACCATCCTACTACTCCTATTCCCTGCCATTGGAGCCCTAG

AAAACAAAATACTTAACTACTAA

> Taeniopygia guttata cytochrome b (cytb) gene, complete cds; mitochondrial

ATGGCCCCAAACCTTCGAAAAAACCACGAACTACTAAAAATCATCAATGACGCCTTAATTGACCTCCCCA

CACCATCAAACATCTCAACATGATGAAACTTCGGGTCACTCCTAGGCATTTGTCTTATTACCCAAATTGT

TACAGGTCTGCTACTAGCTATACACTACACAGCAGACACCTCCCTAGCCTTCTCCTCAGTCGCCCACATA

TGCCGAGACGTACAATTTGGCTGACTAATCCGTAACCTCCACGCAAACGGCGCCTCCTTCTTCTTCATCT

GCATCTACTTCCACATCGGCCGAGGAATTTACTACGGCTCATACCTAAACAAAGAAACCTGAAACGTCGG

AGTTATCCTACTCCTAACCCTCATAGCAACTGCCTTCGTAGGATATGTCCTACCATGAGGACAAATATCA

TTCTGAGGAGCCACAGTAATCACAAACCTACTCTCAGCAATCCCCTACATTGGCCAAACACTAGTAGAAT

GAGCCTGAGGTGGATTCTCAGTAGACAACCCCACACTAACACGATTCTTTGCCCTCCACTTCTTACTCCC

ATTCGTTATCGCAGGACTCACGCTAGTCCACCTCACCTTCCTGCACGAAACAGGCTCAAACAACCCACTA

GGAATCCCATCAGACTGCGACAAAATCCCCTTCCACCCCTACTACACCACAAAAGACATCCTAGGATTTG

CATTAATACTTTCCATCCTAGCCTCACTAGCCCTATTTTCCCCCAACCTGCTAGGAGACCCAGAAAACTT

CACACCCGCTAACCCCCTAGTAACTCCTCCCCATATTAAGCCCGAATGATACTTCCTATTTGCTTATGCC

ATCCTACGATCCATCCCAAACAAACTAGGAGGAGTCCTAGCTCTAGCCGCCTCAATCCTAGTCCTATTCC

TACTCCCTCTACTCCATACATCCAAACTACGATCGATAACTTTCCGACCCCTATCCCAAATCCTATTCTG

ATCCCTAGTAGCCAACGTTCTAATCCTAACCTGAGTCGGCAGCCAACCGGTAGAACACCCATTCATCATC

ATCGGCCAACTAGCCTCACTCTCCTACTTCACGATCATTCTAATTTTATTCCCCCTCGCAGCCCTCCTAG

AGAATAAAATACTGAAACTATAA

> bicolor Tachycineta isolate 50502 cytochrome b (cytb) gene, partial cds; mitochondrial

TCTGTTTCCCACATCTGCCGAGATGTCCAATTTGGCTGACTCATCCGAAACCTCCACGCAAACGGAGCCT

CCTTCTTCTTCATCTGCATCTACTTCCACATCGGACGAGGAATTTACTACGGATCCTACTTAAACAAAGA

AACCTGAAACATCGGAGTAATCCTCCTATTAACCCTCATAGCAACAGCCTTTGTAGGTTACGTCCTACCC

TGAGGACAAATATCATTCTGAGGCGCTACAGTAATTACAAACCTATTCTCAGCAATCCCATATATCGGCC

AAACACTTGTAGAATGAGCATGAGGCGGATTCTCAGTAGACAATCCGACTCTTACCCGATTCTTCGCTCT

ACACTTCCTACTGCCCTTCGTTATCGCAGGCCTCACCATCGTCCACCTAACTCTCCTACATGAAACAGGA

TCAAACAACCCCCTAGGCATCCCCTCAGACTGCGACAAAATCCCGTTCCACCCATATTACTCACAAAAAG

ACATCCTAGGATTCGCACTACTACTCATTCTATTAGCCACCTTAGCCCTATTCTCCCCAAATCTGCTAGG

AGACCCAGAAAACTTCACTCCAGCCAATCCCCTAGCCACCCCTCCACACATCAAACCCGAATGATACTTC

CTATTTGCTTACGCAATTCTACGTTCCATCCCAAACAAACTAGGAGGAGTACTCGCCCTAGCTGCCTCCG

TCCTAGTACTATTCCTAATACCACTACTCCACACCTCCAAGCTACGATCAATAACATTCCGTCCACTATC

ACAAATCCTGTTCTGGACCCTGGTAGCCAACCTCCTTGTCCTAACCTGAGTAGGAAGCCAACCGGTCGAA

CAACCATTCATCATCATCGGCCAACTAGCCTCACTCTCCTACTTCACTATCATCCTCGTCCTCTTT

> Vireo olivaceus voucher FMNH 334603 cytochrome b (cytb) gene, partial cds; mitochondrial

CTTTGGATCACTCCTAGGTATCTGCCTAGTCACACAAATTGTTACAGGCCTACTACTAGCAATACACTAC

ACTGCAGACACCTCCCTAGCATTCACCTCTGTAGCCCACACCTGCCGAAACGTGCAATTYGGATGACTAA

TCCGAAACCTACACGCAAACGGAGCCTCCTTYTTCTTTATCTGCATCTACCTTCACATCGGCCGAGGACT

ATACTACGGCTCATACCTAAACAAAGAAACCTGAAACGTAGGAGTCATCCTCCTACTAACTCTAATAGCA

ACTGCTTTCGTAGGGTACGTCCTTCCCTGAGGACAAATATCATTCTGAGGAGCTACAGTCATCACAAACC

TATTCTCAGCAATCCCATACATCGGACAAACACTAGTAGAATGAGCTTGAGGCGGATTCTCAGTAGACAA

CCCAACACTAACCCGATTCTTTGCCCTACACTTCCTACTACCATTCGTAATCGCAGGACTAACACTAGTT

CACCTCACCTTCCTACACGAAACAGGATCAAACAACCCCCTAGGAATTCCCTCAGACTGTGACAAAATCC

CATTCCACCCATACTACTCTATCAAAGACATTCTAGGCTTTGCCCTAATACTAGCATCACTAGTCGCCCT

AGCCCTATTCTCCCCAAACCTACTAGGAGACCCAGAAAACTTCACACCAGCCAACCCCCTAGCCACCCCA

CCACATATCAAACCTGAATGATATTTCCTATTCGCATACGCTATCCTCCGATCAATCCCCAACAAACTAG

GAGGAGTACTGGCCCTAGCCGCCTCCATCCTAGTCCTATTCCTAATACCACTACTTCACACCTCCAAACA

ACGATCCATAACCTTCCGTCCCCTATCCCAAATCCTCTTCTGAGTGCTGGTTACCAACGTACTCATCCTA

ACTTGAATCGGCAGCCAACCAGTTGAACAGCCATTCATCATCATCGGACAACTAGCTTCACTCAGCTACT

TCACAATCATTCTAGTCCTATTCCCTATCGCAGGTGTACTAGAAAACAAAATACTAAAACTTTAA

> Sterna hirundo cytochrome b (cytb) gene, partial cds; mitochondrial

TGAAACTTCGGATCTCTACTAGGCATTTGCCTATTAACACAGATCCTAACAGGACTTCTACTAGCCATAC

ACTACACCGCAGATACAACCCTAGCTTTTTCATCCGTAGCCCACACATGCCGAAACGTACAATACGGCTG

ATTAATCCGCAATCTCCATGCAAATGGGGCATCATTCTTCTTCATTTGTATCTACTTACATATTGGACGA

GGATTCTACTATGGCTCATACCTATACAAAGAGACATGAAATACAGGAGTTATTCTCTTATTGACCCTAA

TGGCCACCGCCTTCGTAGGGTACGTCCTACCATGAGGACAAATATCTTTCTGAGGTGCTACAGTCATCAC

CAACCTATTCTCAGCAATCCCCTACATCGGCCAAACCCTAGTAGAATGAGCCTGAGGTGGTTTCTCAGTA

GACAATCCAACACTCACCCGATTCTTTGCCCTACACTTCCTTCTCCCATTCATAATTGCAGGTCTCACCC

TTATCCACCTTACCTTTCTCCATGAATCTGGATCGAACAATCCACTGGGTATCCTATCAAACTGCGACAA

AATCCCATTCCACCCCTACTTCTCACTAAAAGATATCCTAGGATTCACTATCATATTCCTCCCACTAACG

ACCCTAGCTCTATTCTCACCAAATCTGTTAGGAGACCCAGAAAACTTTACCCCAGCAAACCCATTAGTTA

CACCTCCCCATATTAAACCCGAATGATATTTCCTATTCGCATACGCTATCCTACGCTCCATCCCTAACAA

ACTAGGGGGTGTATTAGCTTTAGCAGCCTCCGTACTAGTACTATTCCTAAGCCCCCTACTCCATAAGTCC

AAACAGCGTTCAATAACCTTCCGTCCCCTCTCCCAGCTCCTATTNTGAATCCTAGTCGCCAATCTCTTTA

TCCTGACATGAGTAGGCAGTCAACCCGTAGAACATCCATTCATCATCATCGGACAACTAGCTTCCTTAAC

CTACTTCACCATCCTACTGCTCCTATTCCCCGCTATTGGCACCCTAGAAAATAAAATACTTAATTACTAA

> Sula sula cytochrome b gene, mitochondrial gene encoding mitochondrial protein, partial cds

ACCTGAAACACAGGAGTAATTCTCCTACTGACACTAATAGCAACTGCTTTCGTAGGCTATGTCCTACCAT

GAGGGCAAATATCCTTCTGAGGAGCCACAGTCATTACCAACCTATTCTCAGCCATCCCATACATTGGCCA

AACCCTCGTAGAATGAGCTTGAGGCGGATTTTCAGTAGACAACCCTACCCTAACTCGATTCTTTGCTCTA

CACTTCCTCCTCCCATTCATTATCGCAGGTCTCGTCCTAATCCATCTCACATTCCTCCACGAATCAGGCT

CAAATAACCCACTAGGAATCTCATCAAACTCCGACAAAATCCCATTCCACCCCTACTTCACCCTAAAAGA

CATACTAGGATTCATACTCCTTCTACTTCCACTAACAACCATAGCCCTATTCTCTCCCAACCTCCTCGGA

GATCCAGAAAACTTCACCCCAGCAAACCCCCTCGTCACTCCCCCCCACATTAAACCAGAATGATACTTCC

TATTCGCATACGCCATTCTACGCTCAATCCCAAACAAACTAGGAGGAGTACTAGCCCTAGCAGCTTCCGT

CCTAATCCTCTTCCTCAGCCCCCTTCTCCACAAATCCAAACAACGTACAATAACCTTCGTCCCCCTCTCT

CAACTCCTATTCTGAACCTTAGTCGCCAACCTCCTCATCCTAACATGAGTCGGTAGCCAACCTGTAGAGC

ACCCATTCATTATCATCGGCCAACTAGCCTCCCTTACCTACTTCACTATCCTCCTCATCCTCTTCCCCCT

TATCGGAGCCCTAGAAAATAAAATACTCAATTACTAA

> Tachycineta albilinea cytochrome b (cytb) gene, partial cds; mitochondrial gene for mitochondrial product

TACACAGCAGAACACTCACTGGTCTTTGCCTCCGTCTCCCACATCTGTCGAGACGTTCAATTCGGCTGAC

TCATCCGAAACCTACACGCAAATGGAGCCTCCTTCTTCTTCATCTGCATTTACTTCCACATCGGACGAGG

AATTTACTACGGATCCTACCTAAACAAAGAGACCTGAAACATTGGAGTAATCCTCCTACTAGCCCTCATA

GCAACAGCCTTCGTAGGTTACGTACTACCCTGAGGACAAATATCATTCTGAGGGGCTACAGTAATCACAA

ACCTATTTTCAGCAATTCCATACATCGGCCAAACACTTGTAGAGTGAGCATGAGGTGGATTCTCAGTAGA

CAACCCAACCCTCACTGGATTCTTCGCCCTACACTTCCTACTACCCTTCATTATCGCAGGCCTCACCATC

GTCCACCTAACCCTACTACACGAAACAGGATCGAACAACCCCCTAGGCATTCCCTCAGACTGTGACAAAA

TCCCATTCCACCCGTACTACTCCACAAAAGATATCCTAGGGTTCGCACTATTACTCACCCTATTAGCCGC

CCTAGCCTTATTCTCCCCCAACCTCTTAGGAGACCCAGAAAACTTCACTCCAGCCAATCCCCTAGCCACC

CCACCACATATCAAACCTGAATGATACTTCTTATTCGCCTACGCTATCCTACGCTCTATTCCAAACAAAC

TAGGAGGCGTCCTTGCCCTAGCCGCCTCCATCCTAGTTCTATTCCTAATACCACTACTCCACACCTCCAA

ACTACGATCAATAACATTCCGTCCACTATCACAAATCCTATTCTGAACTCTAGTCGCCAACCTCCTCATC

TTAACCTGAGTAGGAAGCCAACCAGTCGAACAACCCTTCATCATCATCGGCCAACTAGCCTCGCTCTCCT

ACTTTACCATTATCCTCATCCTCTTCCCCCTTGTATCCCTCCTAGAAAACC

> Tachymarptis melba voucher UWBM:61463 cytochrome b (cytb) gene, partial cds; mitochondrial

GGCCTCNTACTAGCTATGCACTACACTGCAGACACAACCTTAGCCTTCTCATCAGTAGCCCACACATGCC

GAAACGTCCAATACGGATGACTAATCCGCAATCTGCATGCAAACGGAGCCTCATTCTTCTTCATCTGCAT

CTATCTCCACATCGGACGAGGATTCTACTATGGATCCTACCTCTACAAAGAAACCTGAAACACAGGAGTA

ATCCTACTACTGACCCTTATAGCTACCGCCTTCGTAGGCTATGTCCTCCCCTGAGGACAAATATCCTTCT

GAGGCGCAACAGTCATTACTAACCTCTTCTCAGCAATCCCATACATCGGCCAAACCCTCGTAGAATGAGC

CTGAGGGGGATTCTCAGTAGACAACCCCACATTAACCCGATTCTTCGCCCTACACTTCCTCCTCCCCTTC

CTAATCGCAGGCCTCACCCTCATCCACCTCACCTTCCTCCACGAATCAGGATCAAACAACCCCCTAGGAG

TTGTGTCAAACTGTGACAAAATCCCATTCCACCCCTACTTCTCCACAAAAGACCTTCTAGGATTTATCAT

CATATTCACTCCACTCATAACCCTAGCCCTATTCTCCCCTAACCTCTTAGGAGACCCTGAAAACTTCACC

CCAGCCAACCCACTAGTAACCCCTCCCCATATCAAACCAGAATGGTACTTTCTATTCGCATATGCCATCC

TACGCTCAATCCCCAACAAACTTGGAGGAGTACTGGCTCTTGCCGCTTCCGTACTAATCCTATTCCTATG

TCCATTCCTTCACAAGTCCGAACAACGTACAATAACATTCCGACCCTTATCGCAAATCTTATTCTGAATC

CTAGTAGCCAACCTACTTATCCTAACCTGAGTAGGTAGCCAACCAGT

> Pygoscelis adeliae haplotype PYAD117 cytochrome b (cytb) gene, partial cds; mitochondrial gene for mitochondrial product

ACAGGCATCATCCTCCTACTTACCCTCATAGCAACTGCTTTTGTAGGCTATGTTTTACCATGAGGACAAA

TGTCCTTCTGAGGGGCCACTGTCATCACCAACCTATTCTCGGCTATCCCATATATTGGCCAAACCCTCGT

AGAATGGGCCTGAGGCGGCTTCTCAGTGGACAACCCCACACTAACCCGATTCTTCGCACTACACTTCCTC

CTCCCCTTCGTAATCATAAGCCTCACCCTCATCCACCTTACCTTCCTCCACGAATCAGGCCCAAACAACC

CACTAGGCATCGTAGCCAACTCCGACAAAATTCCATTCCACCCCTACTACTCCACAAAAGATATCTTAGG

GTTCGCATTCATACTCCTTCCATTAACAACCCTCGCCCTATTTTCACCCAACCTACTAGGTGACCCAGAA

AACTTCACCCCAGCAAACCCACTAGTCACACCCCCACATATCAAACCAGAATGATACTTCCTATTCGCAT

ATGCTATCCTACGCTCGATCCCCAACAAACTGGGAGGTGTCCTAGCCTTAGCAGCATCCGTACTAATCCT

ATTCCTAATCCCCCTCCTCCACAAGTCTAAACAGCGCACAATAGCCTTCCGTCCCCTCTCCCAACTCCTA

TTCTGAGCCCTAGTAGCCAACCTTGCCATCCTAACATGGGTAGGTAGCCAACCAGTAGAACACCCCTTCA

TTATCATCGGACAATTAGCTTCCCTCACCTACTTCACCATCCTCCTGATCCTCTTCCCCTCCATCGGGGC

C

> Fregata minor isolate IIL-FMIN348 cytochrome b (cytb) gene, partial cds; mitochondrial

TTTTGTTTTCTAGAGCTCCGACTGCGGGCAGGAGAATAAGGAGGATGGTGAAGTAGGTGAAGGAGGCTAG

TTGACCGATGATAATGAAAGGGTGCTCTACTGGTTGGCTACCGATTCATGTTAGAACGAGGAGGTTGGCG

ACTAGGAGTCAGAATAGTAGTTGGGAGATGGGGCGAAAGGTTATTGTGCGTTGTTTAGATTTATGGAGTA

TGGGTGTCAGGAATAGGATTAATACAGAGGCTGCTAGGGCTAGTACTCCTCCTAGTTTGTTGGGGATTGA

GCGTAGGATGGCGTAGGCGAATAGGAAATATCATTCTGGTTTGATATGTGGGGGTGTTACTAGTGGGTTG

GCTGGAGTGAAGTTTTCTGGGTCTCCTAGAAGGTTAGGTGAGAATAAGGCTAGTATTGTTAGTGGCAGGA

ATATAAGTGTAAAGCCTAGAATATCTTTTAGGGAGAAGTAGGGGTGGAATGGAATTTTGTCGCAGTTTGA

TGTAATTCCTAGGGGATTGTTTGAGCCGGATTCGTGGAGGAAGGTTAGGTGGATTAGGGTGAGGCCTGCG

ATTATAAAGGGAAGGAGGAAGTGTAGGGCGAAGAATCGGGTTAGTGTGGGGTTGTCTACTGAGAAGCCTC

CTCAAGTTCATTCAACTAGGGTTTGTCCGATGTATGGGATTGCTGAGAATAGGTTGGTAATGACTGTAGC

TCCTCAGAAAGATATTTGTCCTCATGGTAGGACATACCCTACGAAGGCAGTTGCTATGAGGGTGAG

> Corvus monedula cytochrome b (cytb) gene, complete cds; mitochondrial

ATGGCCCTAAACTTACGAAAAAATCACCCCCTACTAAAAATCATCAACAACTCCCTAATTGACCTTCCCA

CTCCATCAAACATCTCAGCTTGATGAAACTTCGGATCCCTACTAGGCATCTGCCTCATTACCCAAATCAT

CACAGGCCTACTACTAGCCATGCACTACACAGCAGACACTTCCCTAGCCTTCAGCTCAGTAGCCCACATA

TGCCGAAACGTGCAATTCGGATGACTAATCCGAAATCTCCATGCAAACGGCGCCTCCTTCTTTTTCATTT

GCATTTACCTACATATCGGCCGAGGATTTTACTACGGCTCATACCTAAACAAAGAGACCTGAAACGTCGG

AGTAATTCTCCTTCTAGCCCTTATAGCAACTGCTTTCGTAGGGTATGTTCTACCCTGAGGACAAATATCC

TTCTGAGGAGCTACAGTCATCACAAACCTATTCTCAGCAATCCCATACGTCGGACAAACACTAGTAGAGT

GACTTTGAGGAGGATTCTCAGTAGACAACCCCACACTAACTCGATTCTTCGCCTTCCATTTCCTACTTCC

CTTTGTAATCGCAGGCCTAACACTAGTTCACCTAACCTTCCTTCACGAAACAGGCTCAAACAACCCACTA

GGAATCCCCTCAGACTGTGACAAAATCCCATTCCACCCTTACTACTCTATCAAAGACCTCCTAGGATTTG

CACTAATGCTCATCCCACTGGTCACACTAGCCCTATTCTCCCCAAACCTCCTAGGAGACCCAGAAAATTT

CACGCCCGCCAACCCTCTAGCCACACCCCCACATATTAAACCTGAATGATACTTCCTATTTGCATATGCT

ATTCTCCGATCTATCCCAAACAAACTAGGAGGAGTCCTAGCCCTAGCCGCCTCAGTCCTAGTACTATTCC

TAATCCCCTTCCTCCACGTCTCTAAACAACGTTCCATGACCTTCCGACCTCTCTCACAAATCCTATTCTG

AACCCTAGTCTCAGACCTCCTCATTCTAACATGAGTAGGAAGCCAACCAGTCGAACACCCATTCATCATC

ATCGGCCAACTAGCCTCCATCACCTACTTCACAATCATTCTAATCCTATTCCCCCTTGTGAGTGCTTTAG

AAAACAAAATACTCAACCTCTAA

> Uria lomvia cytochrome b gene, mitochondrial gene encoding mitochondrial protein, partial cds

CTTCGGATCCTTACTAGGCATCTGCCTACTAACACAAATCCTCACAGGACTCCTATTAGCCACACACTAC

ACTGCAGACACAACACTAGCCTTTTCATCCGTTGCCCACACATGTCGAAACGTTCAATATGGCTGACTGA

TTCGAAACCTCCATGCAAACGGAGCATCCTTCTTCTTCATCTGCATTTACCTCCACATTGGACGAGGATT

CTACTACGGCTCGTACCTAAACAAAGAAACCTGAAATACAGGTGTCATTCTCCTACTAGCCCTAATAGCA

ACCGCCTTCGTGGGTTATGTTCTACCATGAGGACAAATATCGTTCTGAGGGGCTACAGTCATTACTAACC

TATTCTCAGCAATCCCCTACATCGGTCAAACCCTCGTAGAATGAGCTTGAGGTGGTTTCTCAGTAGATAA

CCCGACACTAACCCGATTCTTTGCCCTCCATTTCCTCCTCCCATTCATAATTGCAGGTCTCGCCTTCATT

CATCTTACCTTCCTCCACGAATCTGGATCAAATAATCCCCTAGGAATCCTATCTAACTGCGATAAAATTC

CATTCCACCCTTACTTCTCACTAAAAGACATCCTAGGATTCATTATCATATTCCTTCCACTAACAACCCT

AGCCCTATTCTCCCCCAACCTACTAGGTGACCCGGAAAACTTTACCCCCGCAAACCCACTGGTTACACCT

CCCCATATTAAGCCAGAATGATACTTCCTATTTGCATACGCTATCCTACGCTCAATCCCCAATAAATTAG

GCGGAGTACTCGCCCTAGCAGCTTCCGTCCTAGTGCTATTCTTAACCCCCCTACTCCATAAATCTAAGCA

GCGCGCCATAACCTTTCGTCCACTCTCACAACTCCTATTCTGAACCCTAGTCGCCAACCTTCTCATTCTA

ACATGAGTAGGCAGCCAACCCGTAGAGCACCCATTCATCATCATCGGACAACTAGCCTCCCTTGCCTACT

TTACCATTCTGCTTCTCCTATTCCCCATCGTCGGGGCCTTAGAAAATAAAATACTTAACTACTAA

> Hirundo rustica cytochrome b gene, complete cds; mitochondrial

ATGGCCCCCAACCTTCGTAAAAACCACCCGCTACTGAAAATCATCAACGACTCCTTAATCGACCTGCCTA

CCCCATCAAACATCTCAACCTGATGAAACTTCGGCTCATTACTAGGACTATGCCTAGTCATACAAATCGT

CACAGGCCTACTTCTAGCTACCCACTATACAGCAGATACCTCACTAGCCTTCGCCTCTGTAGCCCATATA

TGCCGAGACGTACAGTTTGGCTGACTTATCCGAAACCTCCATGCAAACGGAGCCTCCTTCTTCTTCATCT

GTATCTACCTACACATCGGACGAGGATTCTACTACGGATCCTACCTAAACAAAGAAACTTGAAACGTCGG

AGTAGTACTGCTACTAGCACTAATAGCCACGGCCTTCGTAGGCTACGTCCTGCCCTGAGGACAAATATCA

TTCTGAGGGGCTACAGTAATCACGAACCTATTCTCAGCAATTCCGTACATCGGCCAAACACTTGTAGAAT

GAGCATGAGGAGGGTTCTCAGTAGACAACCCTACCCTAACCCGATTCTTCGCCCTACACTTCCTCCTCCC

ATTCGTCATCGCAGGACTGACCCTAGTACACTTAACCCTACTCCACGAAACAGGATCAAACAACCCACTA

GGAATCCCCTCAGACTGCGATAAAATCCCATTCCACCCATACTACTCCACAAAAGACATCCTAGGATTTA

TCATACTACTCATCGTACTAGCTTCCCTAGCACTATTCTCCCCAAACCTCTTAGGTGACCCGGAAAACTT

CACACCAGCCAACCCCCTGGCTACTCCACCGCACATCAAACCCGAATGATACTTCCTATTCGCCTACGCC

ATCCTCCGATCCATCCCAAACAAACTAGGAGGAGTACTAGCCCTAGCTGCCTCCGTCCTAGTATTATTCC

TAATACCTCTACTCCACACCTCCAAACTGCGATCAATAACATTCCGACCACTATCACAAATCCTATTCTG

GACCCTAGTCGCTAACTTACTTGTCCTAACCTGAGTAGGAAGCCAACCAGTAGAACACCCCTTCATCATC

ATTGGACAACTAGCCTCGCTGTCCTACTTCACCATCATCCTGGTCCTATTTCCACTTGTCTCCATCCTAG

AAAACAAAATACTCAAACTCTAG

> Branta leucopsis voucher IPMB 6970 cytochrome b (cytb) gene, partial cds; mitochondrial

AAATCCTAACAGGTTNACTACTAGCCATACACTACACCGCAGACACCTCACTCGCCTTCGCCTCAGTAGC

CCACACATGCCGAGATGTCCAATACGGATGACTCATCCGCAACCTTCACGCTAACGGCGCTTCATTCTTC

TTCATCTGCATCTACCTGCACATCGGACGAGGCCTCTACTACGGCTCCTACCTGTACAAAGAAACCTGAA

ACACAGGAGTAATCCTCCTACTTACCCTAATAGCAACCGCCTTCGTAGGATATGTCCTGCCATGAGGACA

AATATCATTCTGGGGGGCCACCGTAATCACCAACCTATTCTCAGCCATCCCATACATTGGACAAACTCTA

GTAGAATGAGCCTGAGGAGGATTCTCAGTGGACAACCCAACCCTGACCCGATTCTTCGCCATCCACTTCC

TACTACCCTTCCTAATTGCAGGAATCACCTTAGTCCACCTAACCTTCCTACACGAATCAGGCTCAAACAA

CCCCCTAGGACTCGTATCAGACTGCGACAAAATCCCATTCCACCCATACTTCTCCCTCAAAGACATCCTG

GGACTTACCCTGATAATCACCCCCCTAATAACACTAGCCCTATTCTCACCCAACCTTCTAGGAGACCCAG

AAAACTTCACCCCAGCAAACCCACTAGTAACCCCACCGCACATCAAACCAGAATGATACTTCCTATTCGC

CTACGCCATTCTACGCTCAATCCCAAACAAACTAGGAGGTGTATTGGCACTAGCCGCTTCCGTACTAATC

CTATTCCTAATCCCCTTCCTGCACAAATCAAAACAACGAACAATAACATTCCGACCTCTCTCCCAGCTCC

TATTCTGAATCCTAGTAGCCGACCTCCTCATCCTAACATGAGTAGGAAGCCAGCCCGTCGAACATCCATT

CATCATCATCGGCCAACTTGCCTCAATCGCCTACTTCACCATCCTCCTATTCCTCTTCCCCATCGTAAGT

GCCCTAGAGAACAAAATACTCAA

> Strigops habroptilis mitochondrion cytochrome b, partial cds

TTCGGATCACTCCTAGGGATCTGCCTAGCAACACAAATCCTAACTGGGCTTCTACTTGCCATACACTACA

CTGCAGACAACACCTTAGCATTTTCATCCGTAGCACATACATGCCGAAACGTCCAATACGGATGACTAAT

CCGTAATCTACATGCAAATGGAGCATCATTCTTCTTCATCTGCATCTACCTCCACATCGGACGAGGATTC

TACTACGGCTCCTACCTATACAAAGAAACCTGAAACACAGGAGTTATCCTCCTACTTACCCTCATAGCAA

CTGCCTTCGTCGGCTACGTCCTTCCATGAGGACAAATATCATTCTGGGGAGCCACAGTCATTACTAATCT

ATTCTTAGCCACCCCCTACATAGGACAGACCCTAGTAGAATGGGCATGAGGTGGGGTCTCCGTAGACAAC

CCCACTCTATCCCGATTCTTTGCCCTACCCCTCCTCCTCCCTTTTATAATCACAGGCATAGTCTTCGTCC

ACTTAACCTTTCTCCATGAAACAGGCTCAAACAATCCATTGGGACTCACCTCAGACTGAGACAAAATACC

ATTCCATCCATACTACACAATTAAAGATATCCTAGGATTCGCACTCATACTTCTCCTCCTCACCACCCTA

GCCCTATTCTCACCAAACCTCCTAGGAGACCCAGAAAACTTGACACCAGCTAACCCACTAGCCACACCTC

CACATATCAAACCAGAATGATACTTCCTATTTGCATACGCTATTCTACGATCTATCCCAAACAAACTAGG

AGGAGTCCTAGCCCTAGCTGCCTCCGTCCTAGTACTATTCTTAGCCACTCTTTTTCATAAATCCAAAAAA

CGGAACATGACCTTTCGGCAAATCTCTCAAATCCTATACTGAATCCCAGCAGTTTATTTTCTAATCCTAA

CATGAGTAGGAAGC

> Hydrobates pelagicus cytochrome b (cytb) gene, mitochondrial gene encoding mitochondrial protein, complete cds

ATGGCCCCCAACCCTCGAAAATCACACCCACTACTAAAAATAGTTAACAACTCACTAATTGACCTACCCA

CCCCCTCAAACATCTCTGCCTGATGAAACTTCGGGTCCCTACTAGCCCTCTGCTTAATAACACAGATCCT

AACTGGTCTACTACTAGCCATGCACTACACCGCAGACACAACCCTGGCATTCTCATCAGTCGCACACACA

TGCCGAAACGTACAATACGGCTGACTAATCCGAAACATACATGCAAACGGAGCCTCATTCTTCTTCATCT

GTATCTACATACATATCGGACGAGGATTTTACTACGGCTCCTACCTCCACAAAGAAACCTGAAACACTGG

CATCCTTCTCCTACTCACCCTCATAGCAACTGCCTTCGTAGGCTATGTACTACCATGAGGACAAATATCA

TTCTGAGGTGCTACCGTCATCACCAACATGTTCTCAGCAATCCCGTACATCGGACAGACCATTGTAGAAT

GGGCCTGAGGGGGCTTTTCAGTAGACAACCCTACACTGACCCGATTCTTTGCTCTACATTTCCTCCTTCC

CTTCATAATCGCAGGTCTCACTCTAATCCACCTTACCTTCCTTCACGAGTCCGGCTCAAACAACCCCCTA

GGCATCGTATCAAACTGTGACAAAATCCCGTTCCACCCCTACTACTCCCTAAAAGATATCTTAGGCCTTA

CACTTCTACTCCTACCCCTAACAACAATGGCCCTCTTCTCACCCAATCTACTAGGGGACCCAGAAAACTT

CACCCCAGCAAACCCACTTGTCACACCTCCCCATATCAAACCAGAATGATACTTCCTATTTGCATATGCT

ATCCTCCGCTCAATCCCCAACAAACTAGGAGGAGTACTAGCCCTAGCTGCCTCAGTACTAGTCCTATTCC

TAAGCCCCCTCCTACACAAATCCAAACAACGCACAATGGCCTTCCGCCCCCTCTCCCAACTCCTATTCTG

AACCCTAGTCGCTAACCTACTCATCCTAACATGAATTGGCAGCCAACCCGTAGAGCACCCGTTCATCATC

ATCGGCCAACTAGCCTCTACCACCTACTTCATCATCCTCCTAATCCTTTTCCCCATTACCAGTGCCCTAG

AAAACAAAATACTAAACTTCTAA

> Oceanodroma leucorhoa cytochrome b (cytb) gene, mitochondrial gene encoding mitochondrial protein, complete cds

ATGGCCCCCAACCCTCGAAAATCCCACCCCCTACTAAAAATAATCAACAACTCTCTGATCGACCTACCCA

CCCCTCCAAACATCTCTGCCTGATGAAATTTCGGATCCTTACTGGCCCTCTGCCTGGCAACACAAATCCT

AACTGGCTTATTACTGGCCATACACTACACCGCAGACACAACCCTAGCATTCTCATCCGTCGCACATACA

TGCCGAAACGTACAGTACGGCTGACTAATTCGAAACATACATGCAAACGGAGCCTCATTTTTCTTCATTT

GTATCTATATACATATCGGGCGAGGATTCTACTACGGATCCTATCTCCACAAAGAAACCTGAAACACCGG

AGTCCTTCTCTTACTCACCCTCATAGCAACTGCCTTCGTAGGCTATGTCCTGCCATGAGGACAAATATCA

TTTTGAGGCGCTACAGTCATCACCAACATATTCTCAGCAATCCCATACATCGGACAAACCATTGTAGAGT

GAGCCTGAGGGGGCTTCTCAGTAGACAACCCTACACTAACTCGATTCTTTGCCCTACACTTTCTCCTTCC

ATTCATAATTGCAGGCCTCACTTTAATCCACCTTACCTTCCTCCACGAATCCGGCTCAAACAACCCCCTA

GGCATCATGTCCAACTGTGATAAAATCCCATTTCACCCCTACTACTCCATAAAAGACATCCTAGGTATAG

CACTCCTACTCCTATTACTAACGACCATAGCCCTATTCTCACCCAATCTATTAGGGGACCCAGAAAACTT

CACCCCAGCAAATCCACTTGTCACACCCCCACACATCAAACCAGAGTGATACTTCTTATTTGCATACGCC

ATCCTACGCTCAATCCCCAATAAACTAGGAGGAGTACTAGCCCTAGCTGCTTCCGTACTGATTTTATTCC

TAAGCCCCCTTCTACACAAGTCCAAACAACGCACAATAGCATTCCGCCCCCTCTCCCAGCTCCTATTCTG

AATCCTAGTCGCTAACCTATTTATCCTAACATGAATTGGCAGCCAACCAGTAGAACATCCATTCATCGTC

ATTGGCCAACTAGCCTCCGCCACCTACTTTACCATCCTCTTAGTCCTCTTCCCCATCACCAGTGCCCTAG

AAAACAAAATACTAAACTTCTAG

> Diomedea exulans dabbenena cytochrome b (cytb) gene, mitochondrial gene encoding mitochondrial protein, complete cds

ATGGCCCCCAACCTCCGAAAATCTCACCCCCTACTAAAAATAGTCAATAATTCCCTAATCGACCTACCAA

CCCCATCAAACATCTCTGCTTGATGAAACTTTGGATCTCTCTTAGGCATCTGCCTAGCAACACAAATCCT

AACCGGCCTACTGCTAGCCATACACTACACAGCAGACACAACCCTAGCCTTCTCATCCATTGCCCACACA

TGCCGAAACGTACAGTACGGCTGATTAATCCGAAACCTCCACGCAAACGGAGCATCATTCTTCTTCATCT

GCATCTACCTTCACATCGGCCGCGGATTCTACTACGGCTCATACCTATACAAAGAAACCTGAAACACAGG

AGTCCTACTCCTACTCTCCCTCATAGCAACCGCCTTCGTAGGCTATGTTCTACCATGAGGACAAATATCA

TTCTGAGGGGCTACAGTCATTACTAACCTATTCTCAGCTGTCCCATACATTGGCCAAACCCTCGTAGAAT

GAGCTTGAGGTGGATTTTCAGTAGATAACCCCACCCTAACTCGATTCTTTGCCCTACACTTTCTCCTCCC

GTTCATAATCGCAGGCCTTGTCCTCATCCACCTCACCTTTCTCCACGAATCAGGCTCTAACAATCCACTG

GGAATCGTATCAAACTGCGACAAGATCCCATTCCATCCCTACTTCACCCTAAAAGATATCCTAGGATTCA

CACTTATACTTCTTCCACTAGCAACACTAGCTCTCTTCTCCCCCAACCTATTAGGCGACCCAGAAAACTT

CACACCAGCAAACCCATTAGTTACACCTCCCCATATCAAACCAGAGTGATACTTCCTATTCGCATACGCC

ATTCTACGCTCAATCCCCAATAAACTAGGAGGTGTACTAGCCCTAGCTGCATCCGTCCTAGTTCTATTCC

TAAGCCCCCTCCTACACAAATCCAAACAACGCACAATAGCCTTTCGCCCACTCTCTCAACTTCTATTCTG

ACTCTTAGTTGCCAACCTACTCATCCTGACATGAATCGGCAGCCAACCAGTAGAACACCCATTCATCATC

ATTGGTCAGCTGGCCTCTATCACCTACTTCACCATCCTCTTAATCCTCTTCCCCACTATCGCAGCCCTAG

AAAACAAAATACTTAACTACTAA

> giganteus Macronectes cytochrome b (cytb) gene, mitochondrial gene encoding mitochondrial protein, complete cds

ATGGCCCCAAACCTCCGAAAATCCCACCCCCTACTAAAAATAATTAACAACTCTCTAATTGACCTGCCCA

CCCCCTCAAACATCTCTGCCTGATGAAACTTCGGATCCCTCTTAGGCATCTGCCTAATAACCCAAATCCT

AACCGGCCTATTACTAGCCATGCACTACACTGCCGACACAACCCTAGCTTTCTCATCCGTTGCCCATACA

TGCCGAAATGTACAATATGGCTGACTCATCCGAAATCTACATGCAAATGGAGCCTCATTTTTTTTCATCT

GCATTTACCTACACATTGGACGAGGATTCTACTATGGCTCCTACCTTTACAAAGAAACTTGAAACACAGG

AGTCCTACTCCTACTCACCCTCATAGCAACTGCTTTCGTAGGATATGTCCTACCATGAGGACAAATATCA

TTCTGAGGGGCCACAGTCATCACCAATCTATTCTCGGCCATTCCCTATATTGGCCAGACCCTCGTAGAAT

GGGCCTGAGGGGGATTTTCAGTAGATAACCCCACACTAACCCGATTCTTTGCCCTACACTTCCTCCTCCC

CTTTGCAATTGCAGGACTTACCCTAATTCACCTCACCTTCCTTCACGAATCAGGCTCAAACAACCCCCTA

GGCATCGTATCAAACTGTGACAAAATCCCATTCCATCCCTACTTCACCCTAAAAGACATCCTAGGCTTCG

CACTCATAGTCCTCCCACTAACATCCCTAGCTCTATTCTCCCCTAACCTACTAGGAGACCCAGAAAACTT

TACCCCCGCAAACCCGCTAGTTACACCCCCTCATATCAAACCAGAGTGGTACTTCCTATTCGCATACGCC

ATCCTACGCTCAATCCCCAATAAATTAGGTGGAGTATTAGCTCTAGCGGCCTCCGTACTAGTCCTATTCC

TATCCCCATTCCTCCATAAAGCCAAACAGCGAACAATAACCTTCCGTCCCCTCTCCCAACTCCTATTTTG

AATCCTAGTCACTAACCTATTCATCCTAACATGAGTGGGGAGCCAACCAGTAGAGCATCCATTCATCATC

ATCGGCCAATTAGCCTCCATCACCTACTTTACCATCCTCCTAATCCTCTTCCCCATCATCGGAACCCTAG

AAAACAAAATACTCAACTTCTAA

> Macronectes hallii cytochrome b (cytb) gene, mitochondrial gene encoding mitochondrial protein, complete cds

ATGGCCCCAAACCTCCGAAAATCCCACCCCCTACTAAAAATAATTAACAACTCTCTAATTGACCTGCCCA

CCCCCTCAAACATCTCTGCCTGATGAAACTTCGGATCCCTCTTAGGCATCTGCCTAATAACCCAAATCCT

AACCGGCCTATTACTAGCCATGCACTACACTGCCGACACAACCCTAGCTTTCTCATCCGTTGCCCATACA

TGCCGAAATGTACAATATGGTTGACTCATCCGAAATCTACATGCAAATGGAGCCTCATTCTTTTTCATCT

GCATTTACCTACATATTGGACGAGGATTCTACTATGGCTCCTACCTTTACAAAGAAACCTGAAACACAGG

AATTATCCTTCTACTCACCCTCATAGCAACCGCCTTCGTAGGATATGTCTTACCCTGAGGCCAAATATCA

TTCTGAGGGGCCACAGTCATCACCAATCTATTCTCGGCCATTCCCTATATTGGCCAGACCCTCGTAGAAT

GGGCCTGAGGGGGATTTTCAGTAGATAACCCCACACTAACCCGATTCTTTGCCCTACACTTCCTCCTCCC

CTTTGCAATTGCAGGACTTACCCTAATTCACCTCACCTTCCTTCACGAATCAGGCTCAAACAACCCCCTA

GGCATCGTATCAAACTGTGACAAAATCCCATTCCATCCCTACTTCACCCTAAAAGACATCCTAGGCTTCA

CACTCATAGTCCTCCCACTAACATCCCTAGCTCTATTCTCCCCTAACCTACTAGGAGACCCAGAAAACTT

TACCCCCGCAAACCCGCTAGTTACACCCCCTCATATCAAACCAGAGTGGTATTTCCTATTCGCATACGCC

ATCCTACGCTCAATCCCCAATAAATTAGGTGGAGTATTAGCTCTAGCGGCCTCCGTACTAGTCCTATTCC

TATCCCCATTCCTCCACAAAGCCAAACAGCGAACAATAACCTTCCGTCCCCTCTCCCAACTCCTATTTTG

AATCCTAGTCACTAACCTATTCATCCTAACATGAGTGGGGAGCCAACCAGTAGAGCATCCATTCATCATC

ATCGGCCAATTAGCCTCCATCACCTACTTTACCATCCTCCTAATCCTCTTCCCCATCATCGGAACCCTAG

AAAACAAAATACTCAACTTCTAA

> Thalassarche melanophris melanophris cytochrome b (cytb) gene, mitochondrial gene encoding mitochondrial protein, complete cds

ATGGCCCCCAACCTCCGAAAATCTCACCCTCTACTAAAAATAATCAATAACTCCCTAATCGACCTACCCA

CTCCCTCAAACATCTCTGCTTGATGGAACTTCGGATCCCTCCTAGGCATCTGCCTAATAACACAAATCCT

AACCGGTCTACTACTAGCCATACACTACACAGCAGATACAACTCTAGCCTTCTCATCCATTGCCCATACA

TGCCGAAACGTACAGTACGGTTGATTAATCCGAAACCTTCACGCAAACGGAGCATCATTCTTCTTCATCT

GTATCTACCTTCACATCGGCCGTGGATTCTACTACGGCTCCTACTTGAACAAAGAAACCTGAAACACAGG

AATCCTACTCCTACTCACTCTCATAGCAACTGCCTTCGTAGGGTACGTCCTACCATGAGGACAAATATCA

TTCTGAGGAGCCACAGTCATTACCAACCTATTCTCAGCTATCCCATATATCGGCCAGACCCTCGTAGAAT

GAGCTTGAGGAGGATTTTCAGTAGATAACCCCACCTTAACTCGATTCTTCGCCCTACACTTCCTCCTTCC

ATTCATAATCGCAGGCCTTACCCTCATCCACCTCACCTTCCTTCATGAATCAGGGTCCAACAATCCCCTA

GGAATCCTATCAAACTGCGACAAAATTCCCTTCCACCCCTACTTTACCCTAAAAGATATCCTAGGGTTCA

CACTCATATTCCTACCACTAACAGCCCTAGCCCTATTCTCACCCACCCTGCTAGGCGACCCAGAAAACTT

CACTCCAGCAAACCCATTAGTCACCCCTCCCCACATCAAACCAGAATGATATTTCCTATTTGCATACGCC

ATCCTACGCTCAATCCCCAACAAACTAGGTGGCGTACTGGCCCTGGCTGCATCCGTCCTAGTCCTGTTCC

TAAGCCCCCTTCTCCACAAATCCAAACAACGTACACTAACTTTCCGCCCACTTTCCCAACTCCTATTTTG

ACTCCTAGTCACTAACCTATTCATTCTGACATGAATCGGCAGCCAACCAGTAGAACACCCATTCATTATC

ATTGGCCAACTAGCCTCTATCACCTACTTCACCATCCTCCTTATCCTCTTCCCCACCATCGCAGCCCTAG

AAAACAAGATACTTAACTACTAA

> Phoenicopterus ruber cytochrome b (CYTB) gene, partial cds; mitochondrial

CAATACGGCTGATTGATCCGCAATCTACACGCAAACGGAGCCTCATTTTTCTTCATCTGCATCTACTTCC

ACATTGGACGAGGCTTCTACTACGGCTCCTACCTATACAAAGAAACCTGAAACACAGGAGTCATCCTTCT

ACTCACCCTCATAGCAACTGCCTTCGTAGGGTACGTCTTACCATGGGGACAAATATCATTCTGAGGGGCT

ACAGTCATCACCAACCTATTCTCAGCAATCCCCTACATCGGCCAAACCCTTGTAGAATGAGCCTGAGGGG

GATTCTCTGTAGACAACCCCACACTAACCCGATTCTTCGCCCTGCACTTCCTCCTCCCCTTCGTAATCAC

AGGCCTCACCCTAATCCATCTCACCTTCCTTCACGAAACTGGCTCAAACAATCCTCTAGGAATCGTATCA

AACTGTGACAAAATCCCATTTCACCCCTACTTCTCCCTAAAAGATATCCTGGGATTCATACTTATACTCC

TCCCCCTAATAACCCTTGCCCTATTCTCACCCAACCTCCTAGGAGACCCAGAAAACTTCACGCCCGCAAA

CCCCCTAGTCACACCACCCCACATCAAACCAGAATGATACTTCCTATTCGCATACGCCATTCTACGCTCA

ATCCCCAACAAACTAGGAGGAGTACTGGCCCTAGCCGCTTCCGTTCTAGTCCTATTCTTAAGCCCACTCC

TCCACAAATCCAAGCAACGCACACTAACCTTCCGTCCCCTCTCCCAACTCCTATTCTGAACCCTAGTTGC

CAACCTTTTCATCCTAACATGAGTAGGAAGCCAACCCGTAGAACATCCATTCATCATCATCGGACAACTA

GCCTCCCTCACTTACTTCACCACACTCCTAATCCTTTTCCCCATCATCGGAGCCCTAGAAAACAAAATAC

TCAATTACTAA

> G.fulvus mitochondrial cytb gene

GGGTCTCTTCTAGGAATCTGCCTACTGACACAAATCTTAACCGGCCTCCTACTTGCAATACACTACACCG

CAGACACATCCTTAGCCTTTTCATCCGTTGCTCACACATGCCGAAACGTACAGNACGGATGACTAATCCG

CAACCTACATGCCAACGGAGCATCCTTCTTCTTCATTTGCATCTACCTACACATCGGCCGAGGCCTCTAC

TACGGCTCATACCTATATAAAGAGACCTGAAACACAGGAATTATCCTCTTGCTCACCCTCATGGCAACTG

CCTTCGTAGGTTACGTCCTACCATGAGGACAAATGTCCTTCTGAGGGGCTACAGTCATCACCAACCTATT

CTCAGCCATCCCATACATCGGACAAACCCTTGTAGAATGAGCCTGAGGGGGCTTCTCCGTAGATAACCCC

ACCCTCACCCGATTCTTCGCCCTGCATTTCTTACTTCCATTTGTAATCGCAGGTCTTACTCTAATCCACC

TTACCTTCCTCCACGAATCTGGCTCAAACAACCCCCTAGGCATTATCTCAAACTGCGACAAAATCCCATT

CCACCCATACTTNTCCCTCAAGGACATTCTAGGGTTCGTNCTAATACTACTCCCATTAACAGCCCTAGCC

CTATTCTCCCCAAACCTNTTAGGNGACCCAGAAAACTTCACCCCAGCAAACCCACTAGTCACACCCCCAC

ATATCAAGCCAGAATGATATTTTCTATTCGCGTACGCTATTCTACGCTCAATCCCAAATAAACTAGGAGG

AGTACTAGCCTTAGCTGCCTCCGTACTAATCCTATTCCTAATCCCCTTTCTACACAAATCCAAACAACGC

ACAATAACCTTTCGACCTCTCTCCCAACTCCTATACTGAACCCTAATCGCCAATCTCCTCATCCTCACAT

GAATCGGCAGCCAGCCAGTAGAACACCCCTTCATCATCATCGGCCAACTAGCCTCCATCACCTACTTTAC

TATCCTTCTAGTTCTCTTCCCCTTAACTGGGGCCCTAGAAAATAAA

> Calonectris diomedea borealis cytochrome b mitochondrial

TTGACCGATGATGATGAATGGATGTTCTACTGGTTGGCTACCCACTCATGTCAGGATGAATAGGTTGGCG

ACTAGGATTCAGAATAGAAGTTGGGAAAGGGGGCGGAAGGTTATTGCTCGTTGTTTAGCTTTGTGGAGGA

ATGGGGCTAGGAATAAGACTAGTACTGATGCAGCTAGGGCTAGTACTCCGCCTAATTTGTTCGGGATAGA

ACGTAAGATGGCATATGCGAATAGAAAGTATCATTCTGGCTTGATATGAGGGGGTGTGACTAGTGGGTTT

GCTGGAGTAAAGTTTTCTGGGTCTCCTAGTAGGTTTGGGGAAAATAAGGCTAGGGTTGTTAGTGGAAGGA

ATATAAGTATAAAGCCTAAGATGTCTTTTAGGGTGAAATAGGGGTGAAATGGGATTTTGTCACAGTTTGA

TACAATGCCAAGGGGATTGTTTGAGCCTGATTCGTGGAGGAAGGTGAGATGGATTAGAGTAAGTCCTGCA

ATTATGAAGGGAAGGAGGAAATGTAGGGCGAAGAATCGGGTGAGTGTGGGGTTGTCTACTGAGAATCCTC

CTCATGCCCATTCTACGAGGGTTTGGCCGATGTAGGGAATAGCTGAGAATAGGTTGGTGATGACTGTAGC

TCCTCAGAATGATATTTGGCCTCAAGGTAGGACATATCCTACGAAGGCAGTTGCTATGAGAGTGAGTAGG

AGAATGACCCCTGTGTTTCAGGTTTCTTTGTATAGGTAAGAGCCATAGTAGAATCCTCGTCCGATGTGGA

GGTAAATGCAGATGAAGAAGAATGAGGCTCCGTTTGCATGTAGGTTTCGAATTAGTCAACCATATTGTAC

GTTTCGGCATGTATAGGCAACGGATGAAAAGGCTAGGGTTGTGTCGGCGGTATAGTGCATAGCTAGTAGT

AGGCCAGTTAAAATTTGGGTTAGTAGACATACGCCTAGGAGGGACCC

> Riparia riparia cytochrome b geneTCTGTTTCCCATATCTGCCGAGACGTCCAATTCGGCTGACTAATCCGAAACCTTCATGCAAACGGAGCTT

CCTTCTTCTTCATTTGCATCTACTTCCATATCGGACGAGGATTCTACTACGGATCCTACCTTAATAAAGA

AACCTGAAACGTTGGAGTAGTCCTACTACTAGCACTCATGGCAACAGCCTTCGTAGGCTACGTACTGCCC

TGAGGCCAAATGTCATTCTGAGGGGCTACCGTAATTACAAACCTATTCTCAGCAATCCCATACATTGGCC

AAACACTTGTAGAATGAGCATGAGGGGGCTTCTCAGTAGACAATCCAACCCTCACCCGATTCTTCGCCCT

ACACTTCCTCCTACCATTTGTCATCGCAGGCCTCACCCTTGTCCACCTAACCCTGCTACACGAAACAGGA

TCAAACAACCCCCTAGGCATTCCATCAGACTGCGACAAAATTCCATTCCACCCATACTACTCCACAAAGG

ACATCCTAGGATTCGCACTACTACTAATTACATTAGCCTCCCTCGCCCTCTTTTCACCAAACCTCCTGGG

AGACCCAGAAAATTTCACACCAGCCAACCCCCTAGCCACCCCACCACATATTAAACCTGAATGATACTTC

CTATTCGCCTACGCCATCCTACGATCGATCCCAAACAAACTAGGAGGAGTCCTCGCCCTAGCCGCCTCCG

TACTCGTACTATTCTTAATACCACTTCTCCACACCTCTAAACTCCGATCCATAACATTCCGCCCACTATC

ACAAATCCTGTTCTGATCCCTAGTAGCCAACCTCCTCGTCCTAACCTGAGTAGGAAGCCAACCAGTCGAA

CATCCCTTCATCATTATCGGCCAACTGGCCTCACTCTCCTACTTCACTATCATCCTAATCCTCTTT

> Calidris alpina cytochrome b gene

TTTCGGATCACTCCTAGGCATCTGCCTCATAACACAGATCCTAACTGGCCTTCTACTTGCCATGCACTAT

ACTGCAGATACAACCCTAGCCTTCTCATCCGTCGCCCACACATGCCGAAACGTACAATACGGCTGACTAC

TCCGCAACCTACATGCAAACGGAGCCTCATTCTTTTTCATCTGCATCTACTTCCACATCGGACGAGGCTT

TTACTATGGCTCATATCTATTCAAAGAGACATGAAACACAGGAGTCATCCTCCTCCTAACCCTAATAGCA

ACCGCTTTCGTAGGATATCTCCTGTCCAATTATGTGGTGCGCTGCATATACTTGTCCCCCCCCATACTAC

ATACCATCCATGTTCCAAATCCATTAATCTACAACCGGGCTATACACCTCTCCACCCCTCACCCGCCTCC

AGAGGGTAACCGAAGCAATGAACCTAGGAATATTCACACACACTGTACTAAACCCATCAACCTGTTAGGA

TTATACATTAAAACTCTCTAAAGCGTACGGCAGTGCTTTAACACACGCTATGATTGGTCTAAGTGCAGAC

AGCTCGAAAACTCTCGAAGTGCACACCAGTCGTACCAGTAATGTCCTGCGTTACTAGCTTCAGGCCCATT

CTTTCCCCCTAAACCCTAGCACAACTTGCTCTTTTGCGCCTCTGGTTCCTATGTCAGGGCCATAAATAGG

TTAGTACTCATAACTTGCTCTTTACGAATACATCTGGTTGGCTATATCTCACCATTTTCGTCCGTGATCG

CGGCATTCCAAAATTCTTATACTTTTGGTTCCTTTTTTTTTTNGGGGGTCTTCACAGGTGGCCCACCCAG

CGCAACGGGTGAATACAATTTAAGACCTGAACATCTCGTGCGTTGCGGTCTATATTTTGGCCCTCAGGAG

> Larus andouinii

> Erythrura gouldiae

> Larus crassirostris

> Acrocephalus sechellensis

The tree file was obtained after sequence alignment using Clustal Omega <https://www.ebi.ac.uk>.

**Tree file.**

(((((((((((Calonectris:0.12484,Fregata:0.03344):0.06658,Grus:-0.14572):0.16791,Chrysolophus:0.04151):0.03031,Pygoscelis:0.06819):0.01436,

Sula:0.06572):0.00764,Amazona:0.09040):0.00202,Phoenicopterus:0.05950):0.00167,((((Rissa:0.03296,Larus:0.03440):0.02246,Sterna:0.06015):0.00859,(

Uria:0.06098,Cepphus:0.04620):0.01000):0.01348,Philomachus:0.07911):0.00286):0.00281,(((Hydrobates:0.04871,Oceanodroma:0.05803):0.02647,(((

giganteus:0.00714,Macronectes:0.00773):0.02431,Fulmarus:0.03166):0.03792,(

Diomedea:0.04884,Thalassarche:0.05090):0.02036):0.00215):0.00402,Haematopus:0.06897):0.00081):0.00239,((((jamaicensis:0.02388,Buteo:0.01549):0.02330,

Haliaeetus:0.04891):0.00698,Accipiter:0.05407):0.01049,G.fulvus:0.05972)

:0.02057):0.00064,(((Calidris:0.26266,Strigops:0.09646):0.00788,(((((

Riparia:0.05877,(bicolor:0.04658,Tachycineta:0.05276):0.00639):0.00668,

Hirundo:0.06765):0.01605,Parus:0.06281):0.00460,(((Taeniopygia:0.04357,

Lonchura:0.04415):0.02181,Turdus:0.07257):0.00567,Passerculus:0.07481)

:0.00624):0.00307,(Vireo:0.06821,((coerulescens:0.05094,Aphelocoma:0.04784)

:0.02435,Corvus:0.05773):0.01547):0.00489):0.01850):0.00668,(((Branta:0.06982,((Meleagris:0.08116,Gallus:0.06711):0.00728,Colinus:0.08032):0.01177)

:0.00707,Tachymarptis:0.07108):0.00239,Falco:0.08830):0.00129):0.00379,

Phalacrocorax:0.08008);


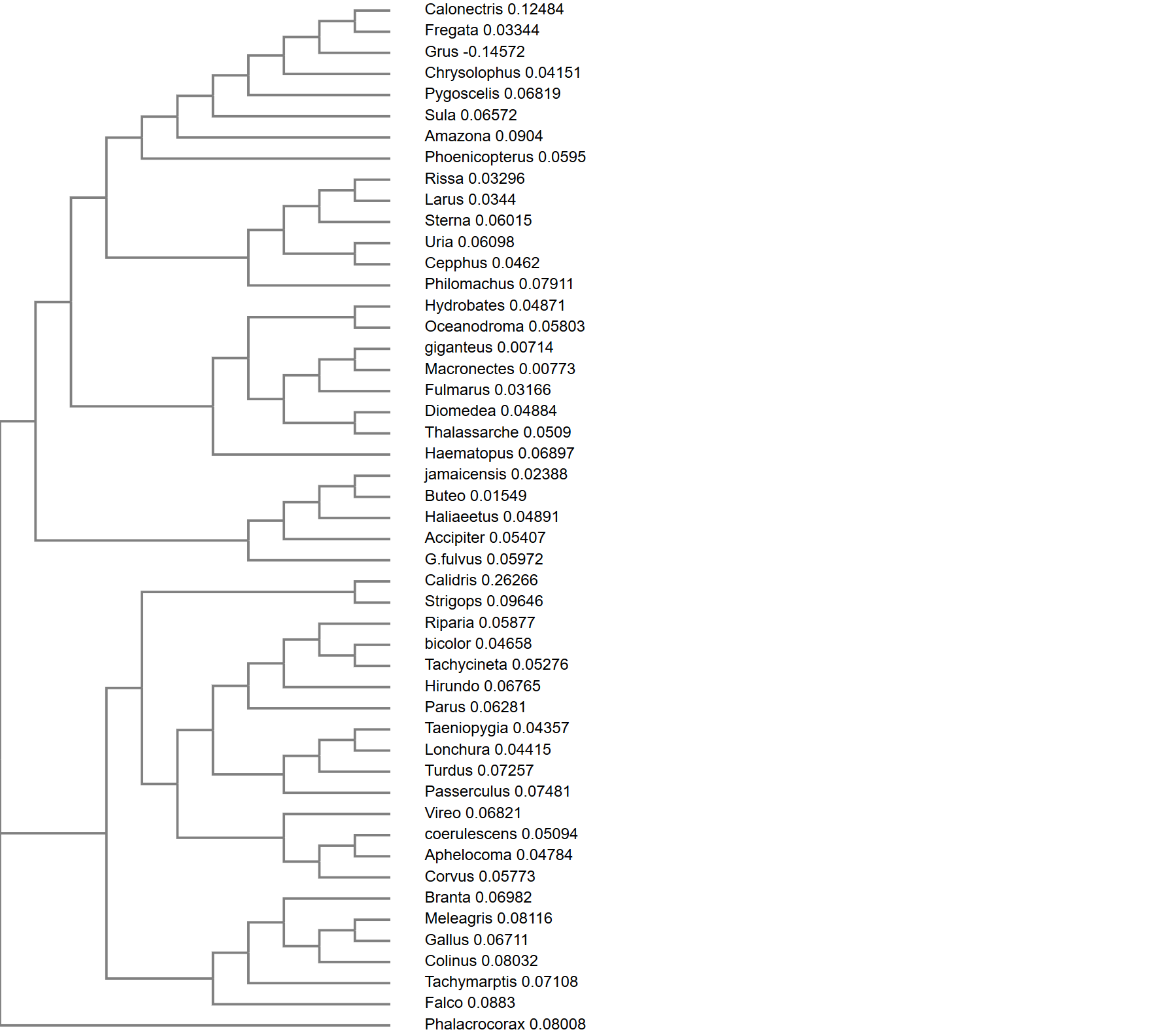

Supplement: Supplementary file 3 — Supplementary Material [file ECE3-11-12908-s004.docx]
