# Supplementary material for: The influence of phylogeny and life history on telomere lengths and telomere rate of change among bird species: A meta‐analysis
Source: Ecol Evol. 2021 Sep 10;11(19):12908–22. doi: 10.1002/ece3.7931 (PMC8495793; doi:10.1002/ece3.7931)
Supplement: Supplementary file 4 — Supplementary Material [file ECE3-11-12908-s002.pdf]

The influence of phylogeny and life history strategies on adult telomere lengths among bird species - supp1 - TEL

09/03/2020

Contents

Load required packages **2**

Set working directory and Load the phylogenic trees (n=100) **2**

Load the consensus tree (n=1 out of 100) **2**

Load the dataset **6**

set.seed **6**

Mean telomere length in adult and the historical relationships among the species, as reflected by the phylogeny **6**

Proportion of Variance explained by phylogeny in Adult mean TL 8

PCA1 and the historical relationships among the species, as reflected by the phylogeny **9**

Proportion of Variance explained by phylogeny in PCA1 12

PCA2 and the historical relationships among the species, as reflected by the phylogeny **13**

Proportion of Variance explained by phylogeny in PCA2 15

PCA3 and the historical relationships among the species, as reflected by the phylogeny **16**

Proportion of Variance explained by phylogeny in PCA3 18

PCA1 + PCA2 + PCA3 and the historical relationships among the species, as reflected by the phylogeny **19**

Posterior distribution of PCs fixed effect 21

Proportion of Variance explained by phylogeny in Telomeres considering PCA1-2-3 as fixed factors 22

Unadjusted Pearson's correlation **31**
Variance and covariance explained by phylogeny (animal) 49
Proportion of variance explained by phylogeny 51
Phylogeny correlation, mode, and 95% CRI (correlation between MeanTL and PCs, explained by phylogeny) 55
Residual correlations (Correlation between MeanTL and PCs, not explained by phylogeny), mode and 95% CRI 58

Load required packages

```
library(ape)
library(apTreeshape)
library(caper)
library(MCMCglmm)
library(phytools)
library(sjPlot)
library(stargazer)
library(dplyr)
library(ggplot2)
```

Set working directory and Load the phylogenic trees (n=100)

Load the consensus tree (n=1 out of 100)

```
library(ape)
Bird.phylo<-read.tree("/home/quentin/Documents/disque D/collab/FC/meta-analyse-review/2020-06-12/consens
Bird.phylo
```

```
##
## Phylogenetic tree with 53 tips and 48 internal nodes.
##
## Tip labels:
##  Macronectes_halli, Macronectes_giganteus, Fulmarus_glacialis, Calonectris_diomedea, Diomedea_exulans
##
## Rooted; includes branch lengths.
```

```
str(Bird.phylo)
```

```
## List of 4
##  $ edge       : int [1:100, 1:2] 54 55 56 57 58 59 60 61 62 62 ...
##  $ edge.length: num [1:100] 0.0962 0.4615 0.2308 0.0769 0.0385 ...
##  $ Nnode      : int 48
##  $ tip.label  : chr [1:53] "Macronectes_halli" "Macronectes_giganteus" "Fulmarus_glacialis" "Calonect
##  - attr(*, "class")= chr "phylo"
##  - attr(*, "order")= chr "cladewise"
```

```r
names(Bird.phylo)
```

```
## [1] "edge"        "edge.length" "Nnode"       "tip.label"
```

```r
plot(Bird.phylo,cex=0.5)
tiplabels(cex=0.25)
nodelabels(cex=0.25)
edgelabels(cex=0.25)
```

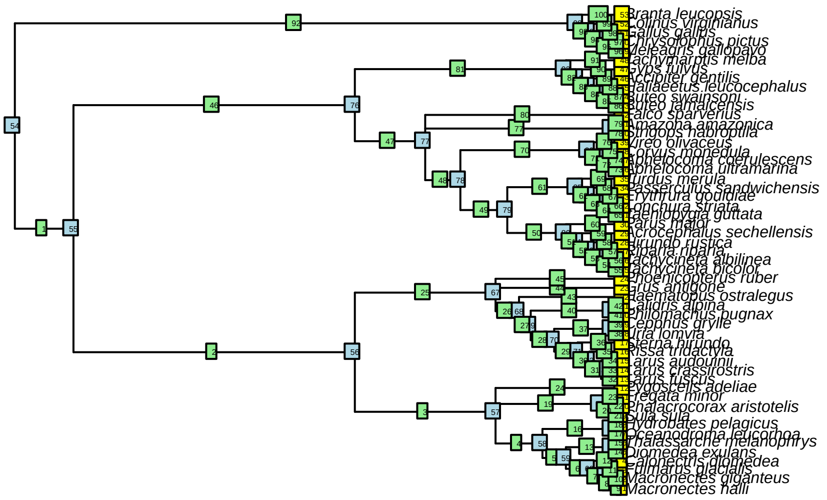


```r
summary(Bird.phylo)
```

```
##
## Phylogenetic tree: Bird.phylo
##
##   Number of tips: 53
##   Number of nodes: 48
##   Branch lengths:
##     mean: 0.07980769
##     variance: 0.01540834
##     distribution summary:
##        Min.    1st Qu.     Median    3rd Qu.       Max.
## 0.01923077 0.01923077 0.03846154 0.07692308 0.92307692
##   No root edge.
##   First ten tip labels: Macronectes_halli
```

```
##                              Macronectes_giganteus
##                              Fulmarus_glacialis
##                              Calonectris_diomedea
##                              Diomedea_exulans
##                              Thalassarche_melanophrys
##                              Oceanodroma_leucorhoa
##                              Hydrobates_pelagicus
##                              Sula_sula
##                              Phalacrocorax_aristotelis
##   No node labels.
```

```
check.species <- function(x) {any(x==Bird.phylo$tip.label)}
print(check.species)
```

```
## function(x) {any(x==Bird.phylo$tip.label)}
```

```
is.ultrametric(Bird.phylo)
```

```
## [1] TRUE
```

```
plot(Bird.phylo,no.margin=TRUE,edge.width=2,cex=0.7)
```

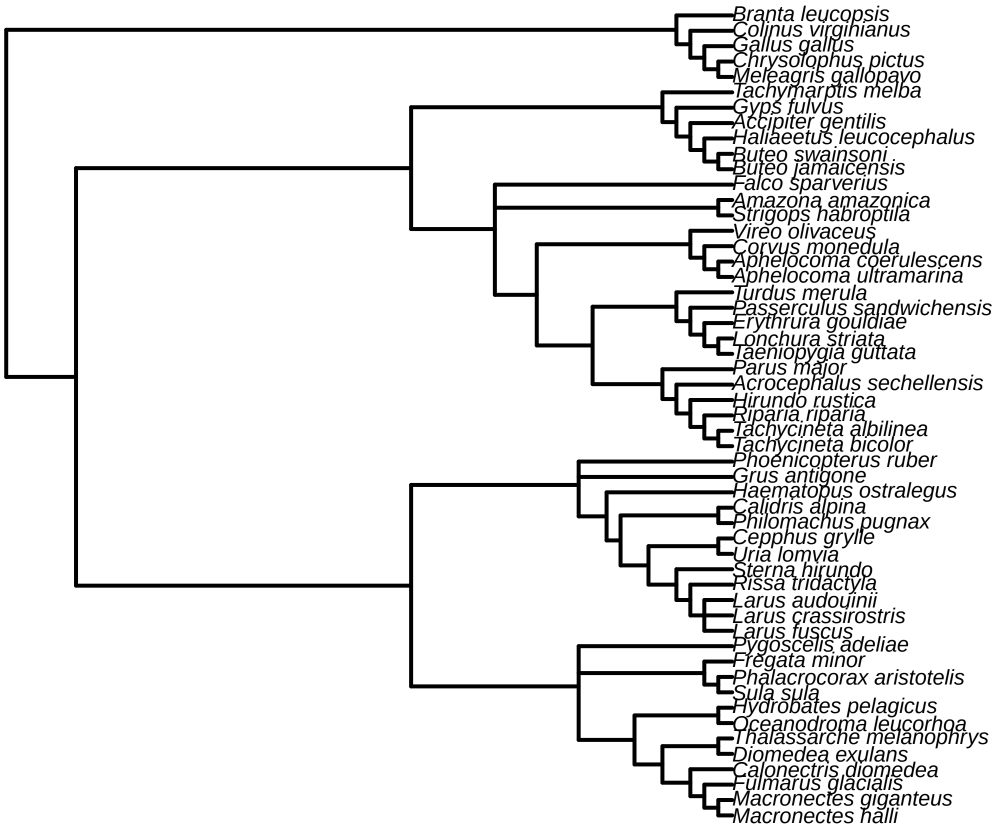

```r
str(Bird.phylo)
```

```
## List of 4
##  $ edge       : int [1:100, 1:2] 54 55 56 57 58 59 60 61 62 62 ...
##  $ edge.length: num [1:100] 0.0962 0.4615 0.2308 0.0769 0.0385 ...
##  $ Nnode      : int 48
##  $ tip.label  : chr [1:53] "Macronectes_halli" "Macronectes_giganteus" "Fulmarus_glacialis" "Calonec
##  - attr(*, "class")= chr "phylo"
##  - attr(*, "order")= chr "cladewise"
```

```r
summary(Bird.phylo)
```

```
##
## Phylogenetic tree: Bird.phylo
##
##   Number of tips: 53
##   Number of nodes: 48
##   Branch lengths:
##     mean: 0.07980769
##     variance: 0.01540834
##     distribution summary:
##        Min.    1st Qu.     Median    3rd Qu.       Max.
## 0.01923077 0.01923077 0.03846154 0.07692308 0.92307692
##   No root edge.
##   First ten tip labels: Macronectes_halli
##                         Macronectes_giganteus
##                         Fulmarus_glacialis
##                         Calonectris_diomedea
##                         Diomedea_exulans
##                         Thalassarche_melanophrys
##                         Oceanodroma_leucorhoa
##                         Hydrobates_pelagicus
##                         Sula_sula
##                         Phalacrocorax_aristotelis
##   No node labels.
```

```r
IA <- inverseA(Bird.phylo, nodes = "TIPS")
IAasreml <- sm2asreml(IA$Ainv, IA$node.names)
summary(IAasreml)
```

```
##       Row            Column          Ainverse
##  Min.   : 1.00   Min.   : 1.00   Min.   :-25.367524
##  1st Qu.:24.00   1st Qu.: 7.00   1st Qu.: -0.026630
##  Median :35.00   Median :15.00   Median : -0.000968
##  Mean   :32.58   Mean   :17.09   Mean   :  0.441079
##  3rd Qu.:42.00   3rd Qu.:25.00   3rd Qu.: -0.000160
##  Max.   :53.00   Max.   :53.00   Max.   : 30.973672
```

Load the dataset

```
Bird.data <- read.csv("/home/quentin/Documents/disque D/collab/FC/meta-analyse-review/2020-06-12/2020-1
Bird.data$animal<-Bird.data$Latin
Bird.data$Latin %in% Bird.phylo$tip.label
```

```
##  [1] TRUE TRUE TRUE TRUE TRUE TRUE TRUE TRUE TRUE TRUE TRUE TRUE TRUE TRUE TRUE
## [16] TRUE TRUE TRUE TRUE TRUE TRUE TRUE TRUE TRUE TRUE TRUE TRUE TRUE TRUE TRUE
## [31] TRUE TRUE TRUE TRUE TRUE TRUE TRUE TRUE TRUE TRUE TRUE TRUE TRUE TRUE TRUE
## [46] TRUE TRUE TRUE TRUE TRUE TRUE TRUE TRUE
```

```
dim(Bird.data)
```

```
## [1] 53 64
```

set.seed

```
set.seed(36849)
```

Mean telomere length in adult and the historical relationships among the species, as reflected by the phylogeny

```
p.var1=var(Bird.data$Mean.telomere.adult)
prior1 <-list(G=list(G1=list(V=(diag(1)*0.2*p.var1),n=1)),
                      R=list(V=(0.8*p.var1),n=2))
set.seed(1)
m11.mcmc <- MCMCglmm(Mean.telomere.adult ~1, random = ~animal, mev=Bird.data$mev ,
                     data = Bird.data, prior=prior1,
                     pedigree = Bird.phylo, nitt = 105000, thin = 50, burnin = 5000,
                     pr = TRUE, pl = TRUE, verbose = TRUE, DIC  = TRUE)
```

```
summary(m11.mcmc)
```

```
##
##  Iterations = 5001:104951
##  Thinning interval  = 50
##  Sample size  = 2000
##
##  DIC: 393.327
##
##  G-structure:  ~animal
##
##        post.mean l-95% CI u-95% CI eff.samp
## animal     15.91   0.8529    49.39     1398
```

```
##
##  R-structure:  ~units
##
##       post.mean l-95% CI u-95% CI eff.samp
## units     90.35    58.14    128.1     2000
##
##  Location effects: Mean.telomere.adult ~ 1
##
##             post.mean l-95% CI u-95% CI eff.samp pMCMC
## (Intercept)    14.030    8.928   18.690     2000 0.001 ***
## ---
## Signif. codes:  0 '***' 0.001 '**' 0.01 '*' 0.05 '.' 0.1 ' ' 1
```

```
par(mar=c(1,4,4,1))
plot(m11.mcmc)
```

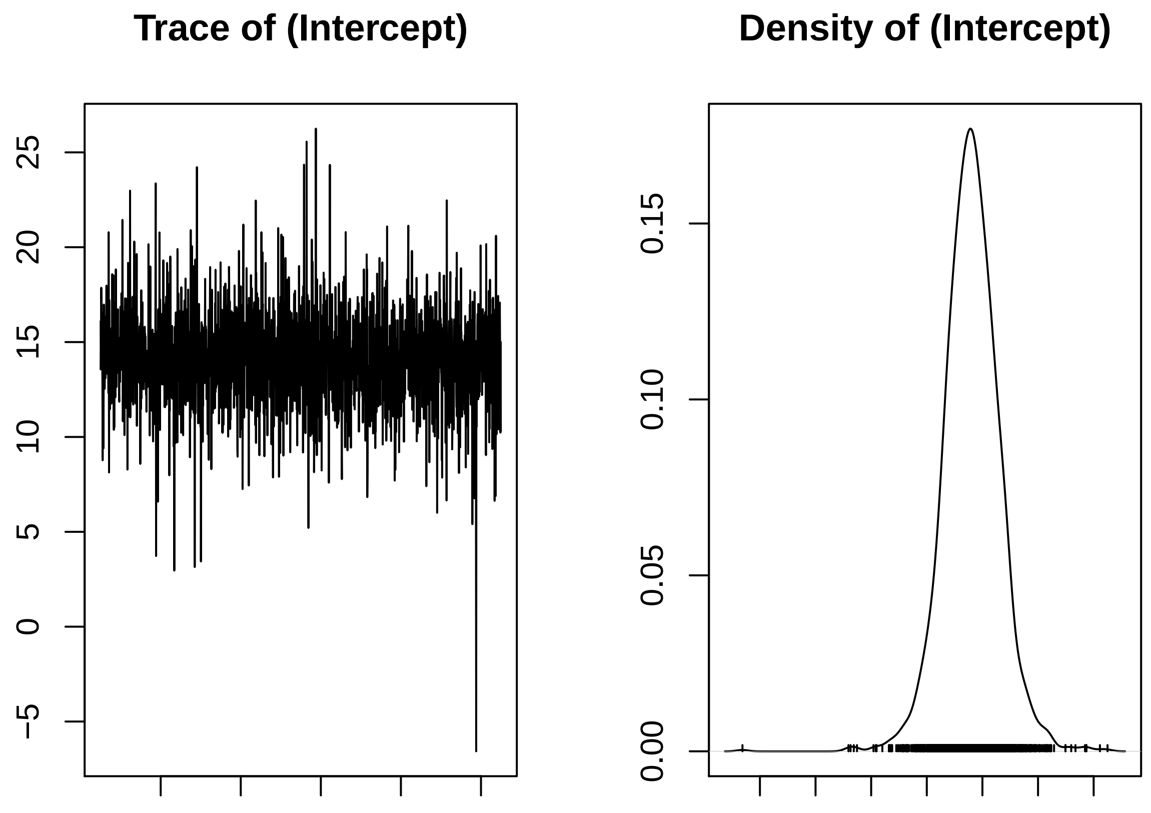

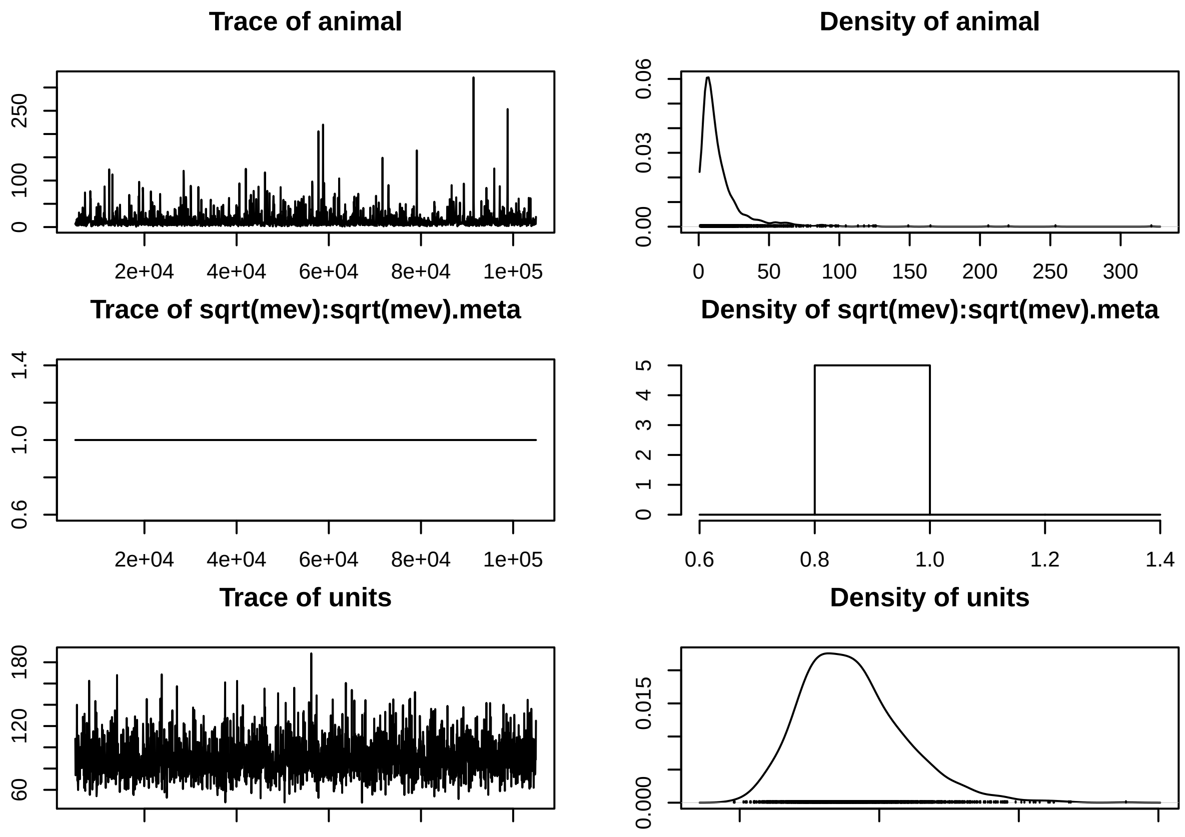

Proportion of Variance explained by phylogeny in Adult mean TL

```
TL_phylogeny<-m11.mcmc$VCV[,'animal']/(m11.mcmc$VCV[,'animal']+m11.mcmc$VCV[,'units']+m11.mcmc$VCV[,"sq
posterior.mode(TL_phylogeny)
```

```
##       var1
## 0.07397586
```

```
effectiveSize(TL_phylogeny)
```

```
##     var1
## 1310.431
```

```
HPDinterval(TL_phylogeny)
```

```
##           lower     upper
## var1 0.01350046 0.3744376
## attr(,"Probability")
## [1] 0.95
```

```
hist(TL_phylogeny, breaks=15)
abline(v=posterior.mode((TL_phylogeny)), col="red")
abline(v=median((TL_phylogeny)), col="blue")
abline(v=mean((TL_phylogeny)), col="green")
```

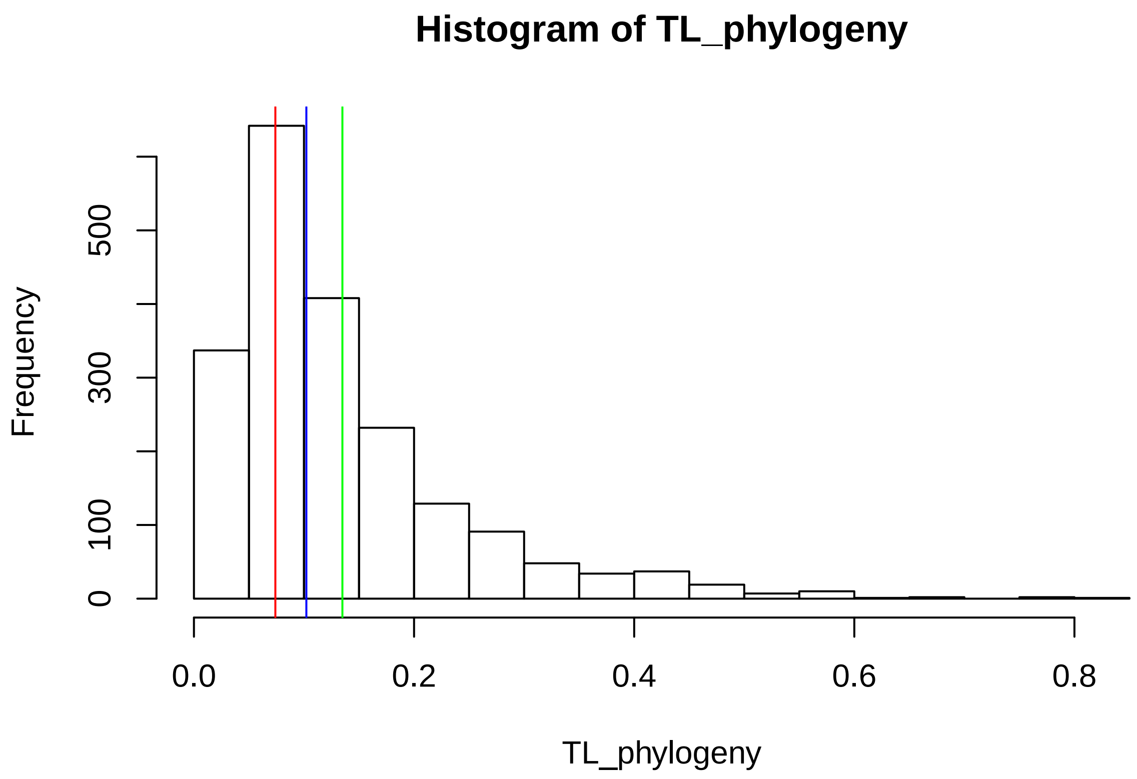

PCA1 and the historical relationships among the species, as reflected by the phylogeny

#defining priors This first line specifies the dependent variable, cbind(PCA1, PCA2), and the fixed effects, -1 + trait + Modality. The term trait is not part of the original data set– rather, it's a reserved term in the MCMCglmm package. trait comes from phylogenetics, where the dependent variable is some observed trait in an organism. For our purposes, including the trait term tells the model to estimate an intercept for each of the observed outcomes. We follow the advice from the tutorial and enter this as -1 + trait. This guarantees an intercept for each non-baseline level of the outcome, rather than an overall intercept term with an offset for k-2 levels.

```
p.var1=var(Bird.data$PCA1)
prior1 <-list(G=list(G1=list(V=(diag(1)*0.2*p.var1),n=1)),
                       R=list(V=(0.8*p.var1),n=2))
```

The above specifies uninformative priors where most weight is divided such that 20% of the variance in each trait is placed on the between-subject level and the remaining on the residual variance. Those are non informative priors, but still we excpect that there is more variation between individual/species, that within.

As always in Bayesian analysis, one needs to verify that prior specifications do not determine the result by evaluating the sensitivity of inferences to different priors.

```
set.seed(1)
m22.mcmc <-MCMCglmm(PCA1 ~ 1, random = ~animal, mev=Bird.data$mev , data = Bird.data, prior=prior1 ,fam
```

```
summary(m22.mcmc)
```

```
##
##  Iterations = 5001:104951
##  Thinning interval  = 50
##  Sample size  = 2000
##
##  DIC: 206.9867
##
##  G-structure:  ~animal
##
##        post.mean l-95% CI u-95% CI eff.samp
## animal     10.37    1.644     21.4     1942
##
##  R-structure:  ~units
##
##       post.mean l-95% CI u-95% CI eff.samp
## units       2.3   0.9158    3.945     1978
##
##  Location effects: PCA1 ~ 1
##
##             post.mean l-95% CI u-95% CI eff.samp pMCMC
## (Intercept)    0.1518  -3.0162   3.5963     2000 0.925
```

```
par(mar=c(1,4,4,1))
plot(m22.mcmc)
```

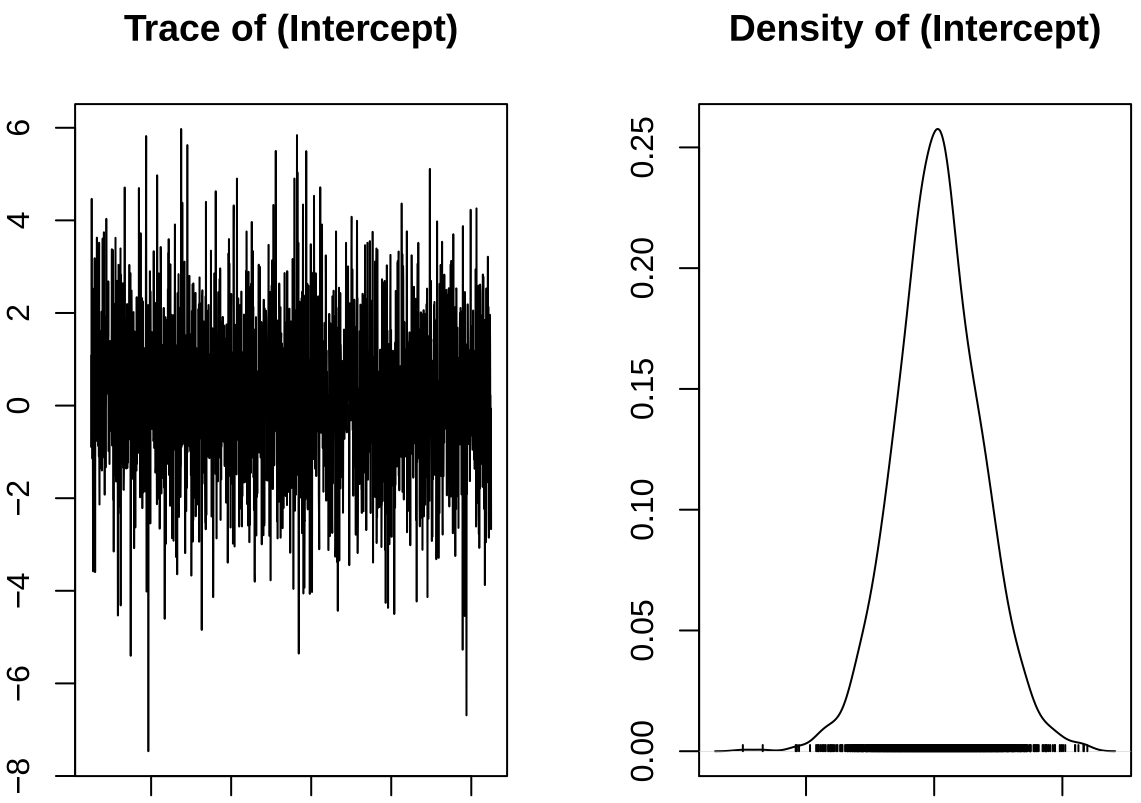
Trace of (Intercept)
6
4
2
0
−2
−4
−6
−8
Density of (Intercept)
0.25
0.20
0.15
0.10
0.05
0.00

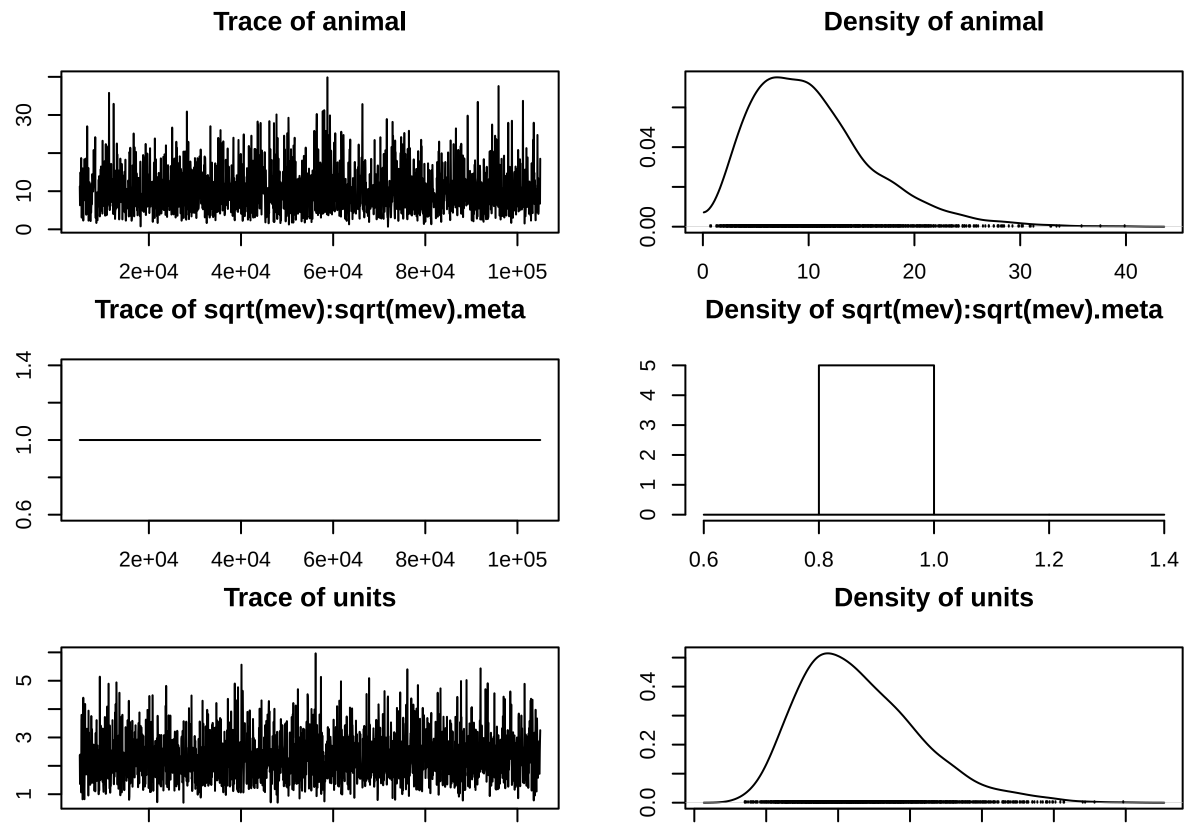

Proportion of Variance explained by phylogeny in PCA1

```
PCA1_phylogeny<-m22.mcmc$VCV[,'animal']/(m22.mcmc$VCV[,'animal']+m22.mcmc$VCV[,'units']+m22.mcmc$VCV[,"s
posterior.mode(PCA1_phylogeny)
```

```
##      var1
## 0.8032231
```

```
effectiveSize(PCA1_phylogeny)
```

```
##     var1
## 1833.804
```

```
HPDinterval(PCA1_phylogeny)
```

```
##          lower     upper
## var1 0.3990639 0.9202694
## attr(,"Probability")
## [1] 0.95
```

```r
hist(PCA1_phylogeny, breaks=15)
abline(v=posterior.mode((PCA1_phylogeny)), col="red")
abline(v=median((PCA1_phylogeny)), col="blue")
abline(v=mean((PCA1_phylogeny)), col="green")
```

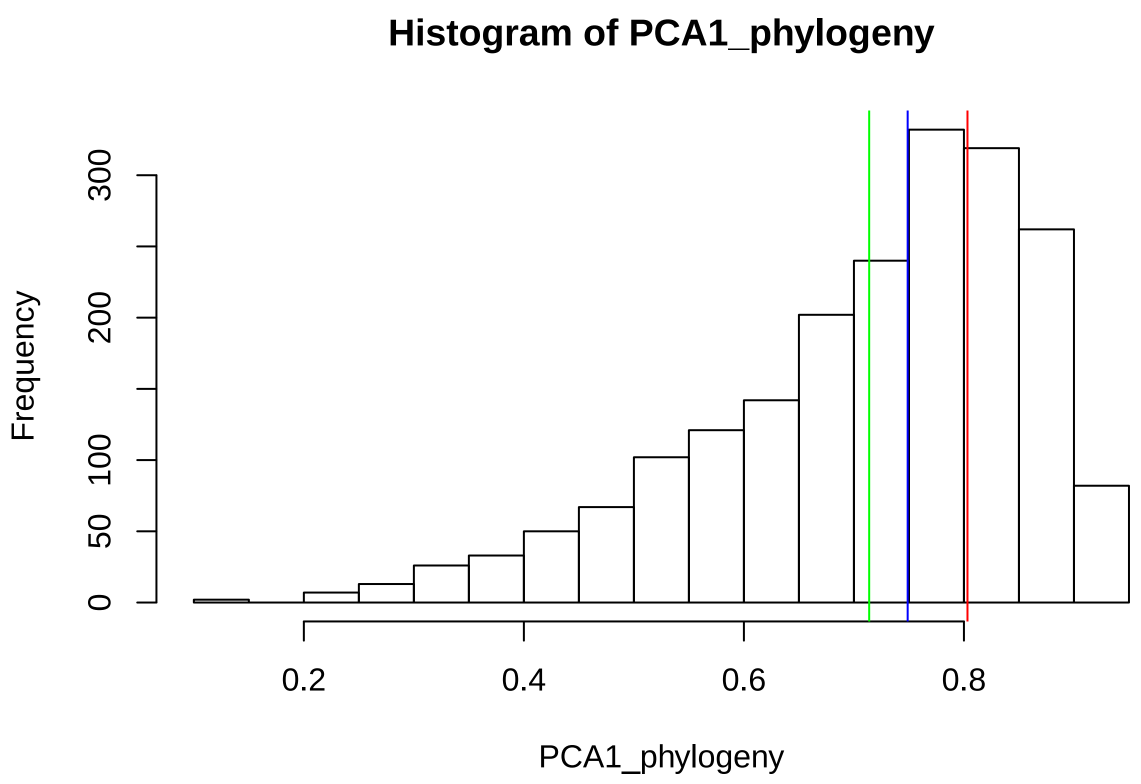

PCA2 and the historical relationships among the species, as reflected by the phylogeny

```r
p.var1=var(Bird.data$PCA2)
prior1 <-list(G=list(G1=list(V=(diag(1)*0.2*p.var1),n=1)),
                      R=list(V=(0.8*p.var1),n=2))
set.seed(1)
m33.mcmc <- MCMCglmm(PCA2 ~ 1, random = ~animal , mev=Bird.data$mev , data = Bird.data,
                    family=c("gaussian"), prior=prior1, pedigree = Bird.phylo, nitt = 105000, thin = 50
                     burnin = 5000, pr = TRUE, pl = TRUE, verbose = TRUE, DIC  = TRUE)
```

```r
summary(m33.mcmc)
```

```
##
##  Iterations = 5001:104951
##  Thinning interval  = 50
```

```
##  Sample size  = 2000
##
##  DIC: 117.4362
##
##  G-structure:  ~animal
##
##        post.mean l-95% CI u-95% CI eff.samp
## animal     1.439    0.251    3.033     2000
##
##  R-structure:  ~units
##
##       post.mean l-95% CI u-95% CI eff.samp
## units    0.3931   0.1797   0.6661     2000
##
##  Location effects: PCA2 ~ 1
##
##             post.mean l-95% CI u-95% CI eff.samp pMCMC
## (Intercept)    0.4437  -0.8149   1.6801     3583 0.458
```

```
par(mar=c(1,4,4,1))
plot(m33.mcmc)
```

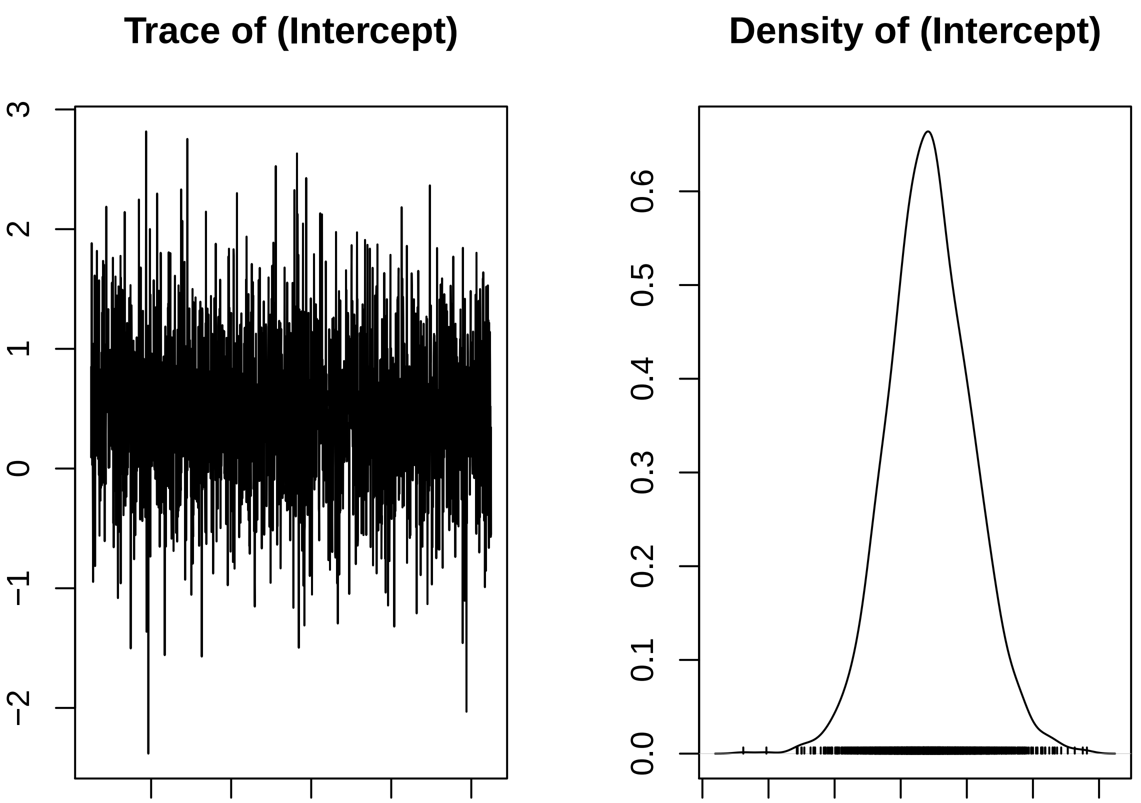

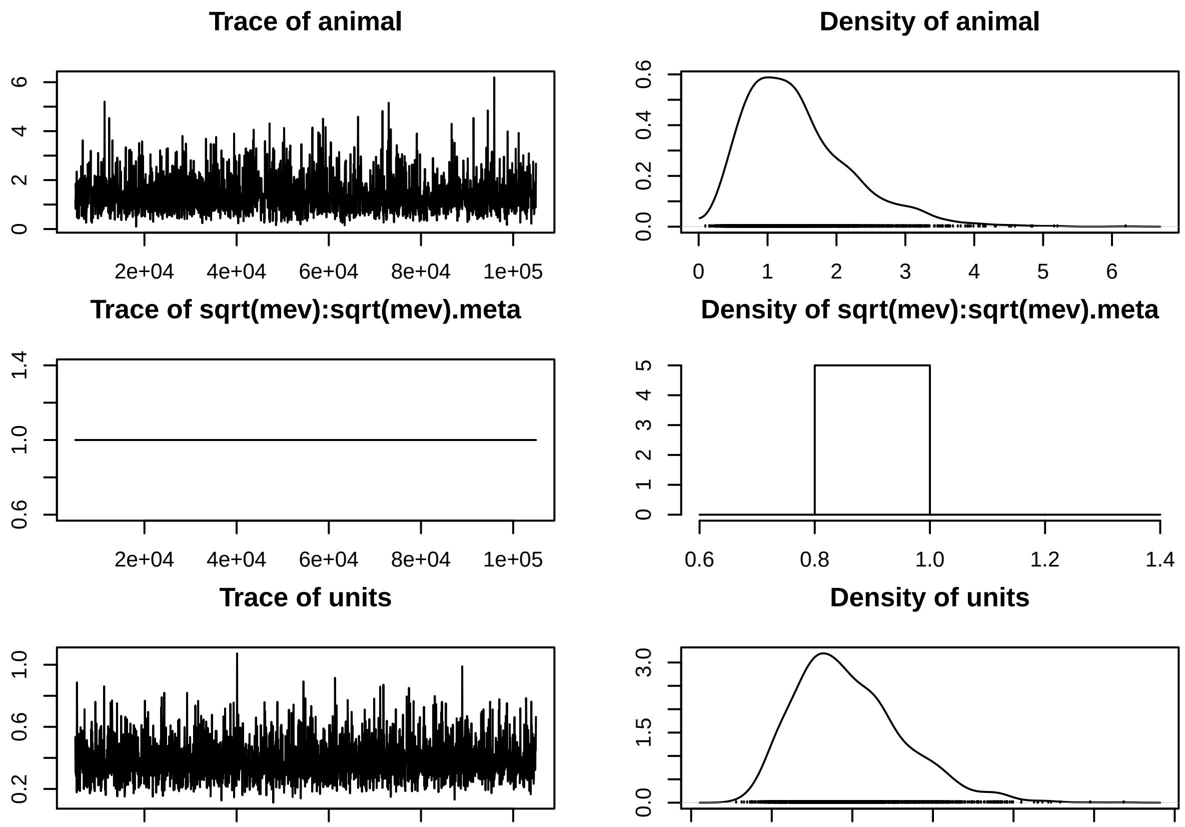

Proportion of Variance explained by phylogeny in PCA2

```
PCA2_phylogeny<-m33.mcmc$VCV[,'animal']/(m33.mcmc$VCV[,'animal']+m33.mcmc$VCV[,'units']+m33.mcmc$VCV[,"
posterior.mode(PCA2_phylogeny)
```

```
##      var1
## 0.4623099
```

```
effectiveSize(PCA2_phylogeny)
```

```
## var1
## 2000
```

```
HPDinterval(PCA2_phylogeny)
```

```
##          lower     upper
## var1 0.2038243 0.7227777
## attr(,"Probability")
## [1] 0.95
```

```r
hist(PCA2_phylogeny, breaks=15)
abline(v=posterior.mode((PCA2_phylogeny)), col="red")
abline(v=median((PCA2_phylogeny)), col="blue")
abline(v=mean((PCA2_phylogeny)), col="green")
```

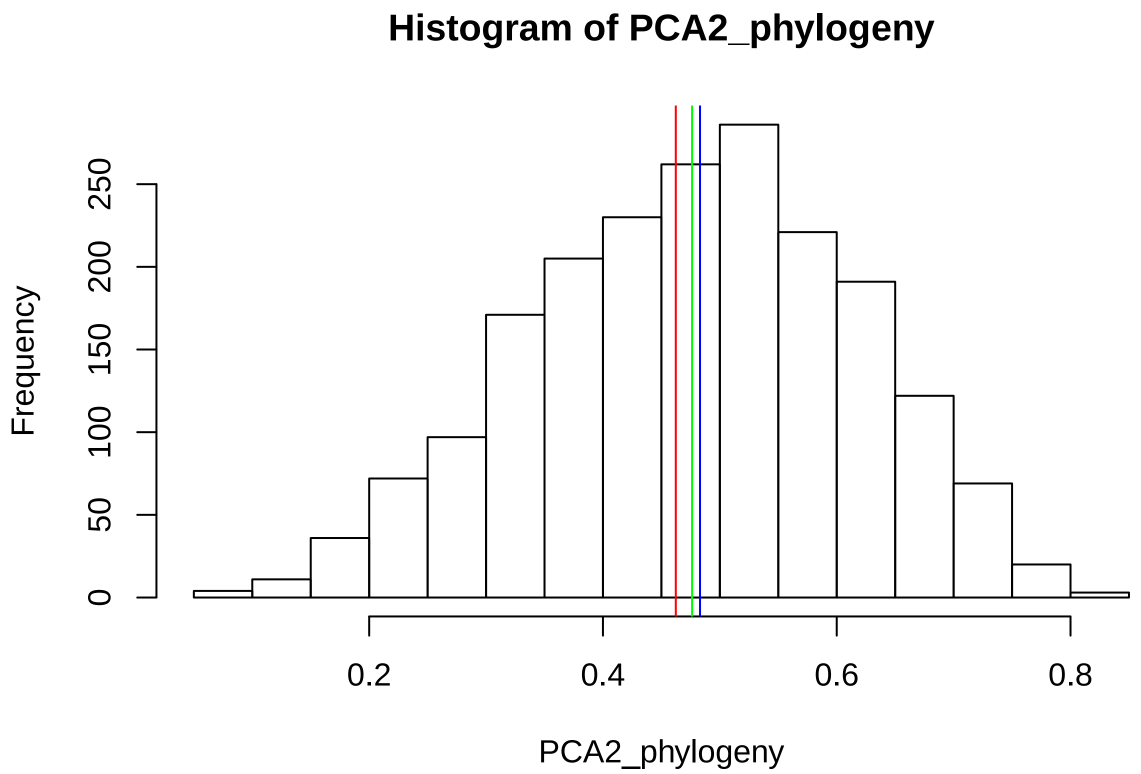

PCA3 and the historical relationships among the species, as reflected by the phylogeny

```r
p.var1=var(Bird.data$PCA3)
prior1 <-list(G=list(G1=list(V=(diag(1)*0.2*p.var1),n=1)),
                       R=list(V=(0.8*p.var1),n=2))

set.seed(1)
m44.mcmc <- MCMCglmm(PCA3 ~ 1, random = ~animal, mev=Bird.data$mev, data = Bird.data,
                     prior=prior1,
                     family=c("gaussian"), pedigree = Bird.phylo, nitt = 105000, thin = 50,
                     burnin = 5000, pr = TRUE, pl = TRUE, verbose = TRUE, DIC  = TRUE)
```

```r
summary(m44.mcmc)
```

```
##
```

```
##  Iterations = 5001:104951
##  Thinning interval  = 50
##  Sample size  = 2000
##
##  DIC: 153.7216
##
##  G-structure:  ~animal
##
##        post.mean l-95% CI u-95% CI eff.samp
## animal    0.3952  0.01604    1.411     1144
##
##  R-structure:  ~units
##
##       post.mean l-95% CI u-95% CI eff.samp
## units    0.8621   0.4621    1.286     1777
##
##  Location effects: PCA3 ~ 1
##
##             post.mean l-95% CI u-95% CI eff.samp pMCMC
## (Intercept)   0.03394 -0.65944  0.77686     2000 0.941
```

```
par(mar=c(1,4,4,1))
plot(m44.mcmc)
```

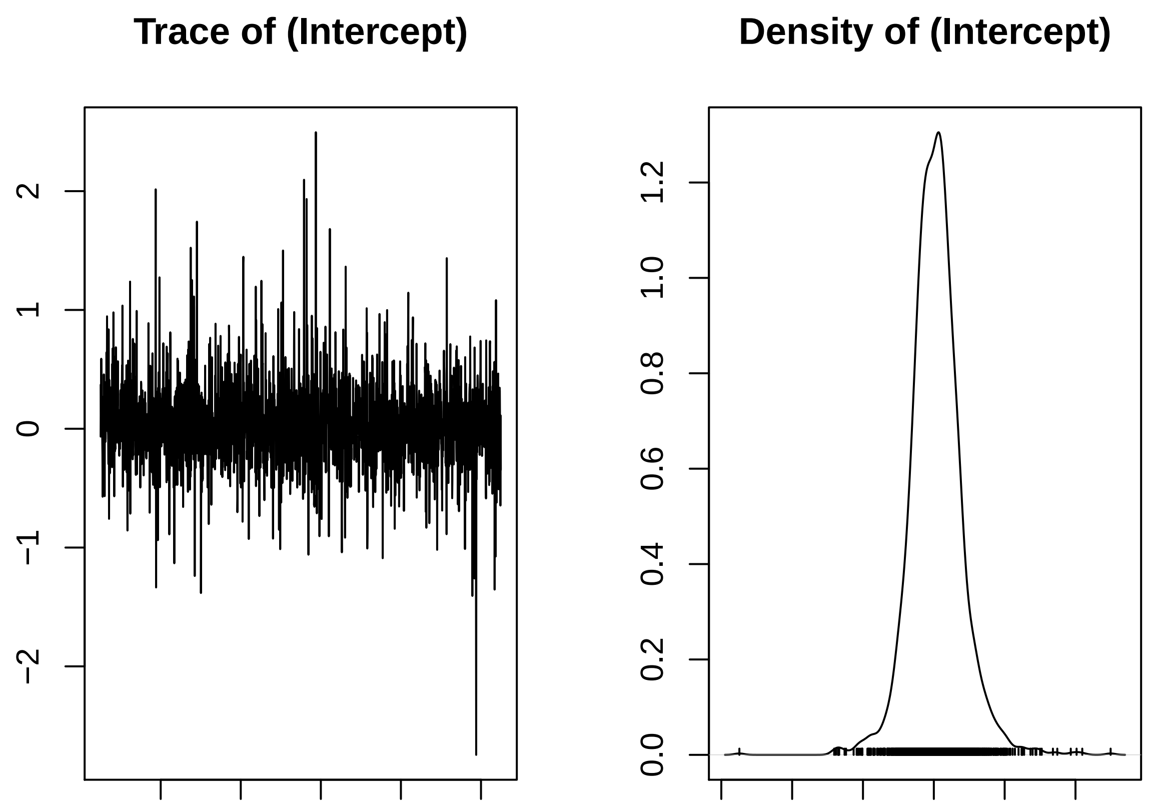

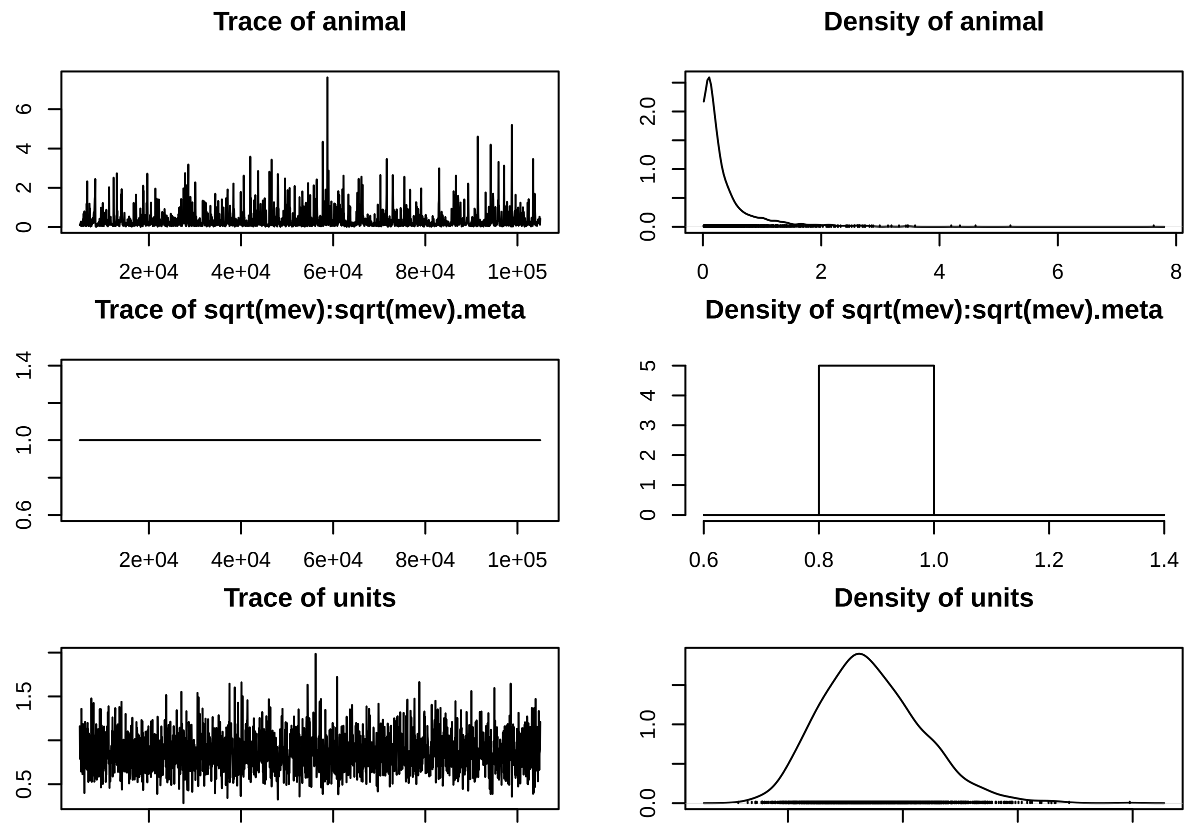

Proportion of Variance explained by phylogeny in PCA3

```
PCA3_phylogeny<-m44.mcmc$VCV[,'animal']/(m44.mcmc$VCV[,'animal']+m44.mcmc$VCV[,'units']+m44.mcmc$VCV[,"

posterior.mode(PCA3_phylogeny)
```

```
##       var1
## 0.04244583
```

```
effectiveSize(PCA3_phylogeny)
```

```
##    var1
## 945.583
```

```
HPDinterval(PCA3_phylogeny)
```

```
##           lower    upper
## var1 0.00757482 0.464313
## attr(,"Probability")
## [1] 0.95
```

```r
hist(PCA3_phylogeny, breaks=15)
abline(v=posterior.mode((PCA3_phylogeny)), col="red")
abline(v=median((PCA3_phylogeny)), col="blue")
abline(v=mean((PCA3_phylogeny)), col="green")
```

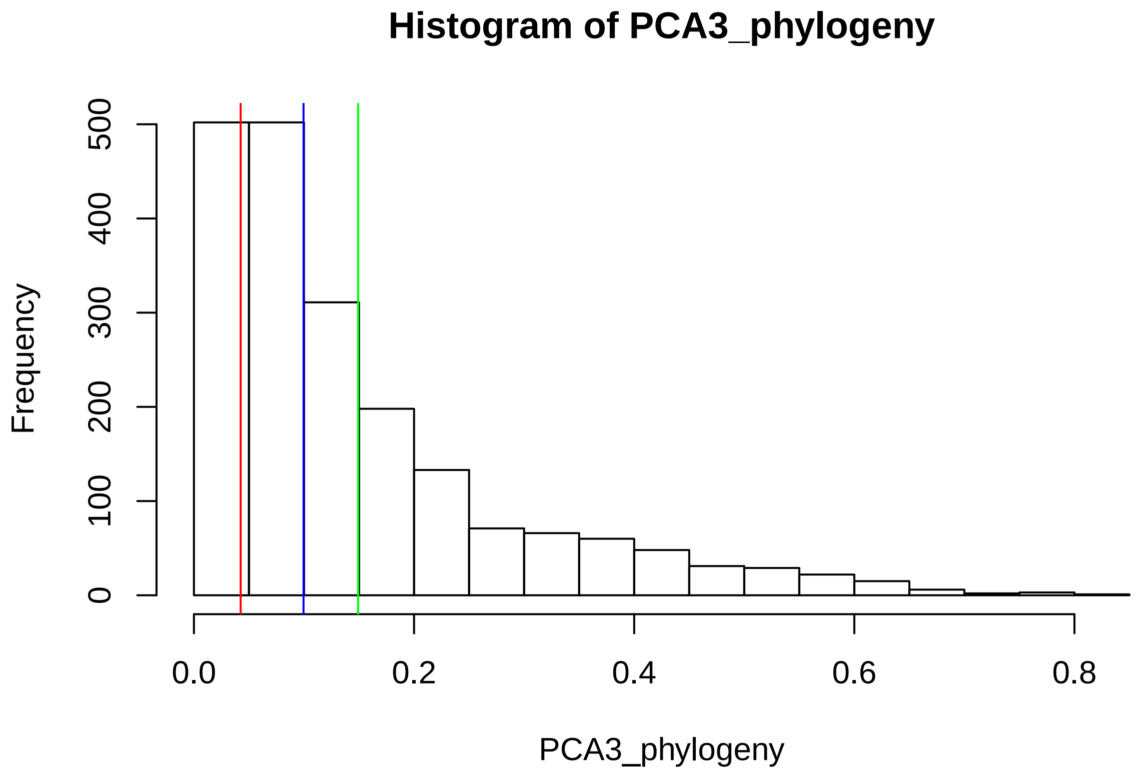

PCA1 + PCA2 + PCA3 and the historical relationships among the species, as reflected by the phylogeny

```r
p.var1=var(Bird.data$Mean.telomere.adult)
prior1 <-list(G=list(G1=list(V=(diag(1)*0.2*p.var1),n=1)),
                       R=list(V=(0.8*p.var1),n=2))


m55.mcmc <- MCMCglmm(Mean.telomere.adult ~PCA1+PCA2+PCA3, random = ~animal   , mev=Bird.data$mev,
                    data = Bird.data, prior=prior1,
                    pedigree = Bird.phylo, nitt = 105000, thin = 200, burnin = 5000,
                    pr = TRUE, pl = TRUE, verbose = TRUE, DIC  = TRUE)
```

```r
oneModel <- clean.MCMC(m55.mcmc)  # get all the info from summary(modelName)
oneModel$modelName <- getName.MCMC(m55.mcmc)  # add the model's name in a new column
oneModel<-oneModel[,-c(5)]
stargazer(oneModel, type = "text", summary = FALSE)
```

```
##
## ==============================================================================
##           variable         post.mean l.95..CI u.95..CI pMCMC  effect  modelName
## ------------------------------------------------------------------------------
## 1       (Intercept)         13.756    8.803    19.559  0.004  fixed   m55.mcmc
## 2           PCA1            -0.849   -1.953    0.310  0.140  fixed   m55.mcmc
## 3           PCA2             1.714   -1.132    4.524  0.224  fixed   m55.mcmc
## 4           PCA3            -0.225   -2.795    2.093  0.848  fixed   m55.mcmc
## 5          animal           19.823    1.634    62.932         random  m55.mcmc
## 6 sqrt(mev):sqrt(mev).meta     1         1        1           random  m55.mcmc
## 7          units            90.783   55.658   129.069        residual m55.mcmc
## ------------------------------------------------------------------------------
```

```r
par(mar=c(1,4,4,1))
plot(m55.mcmc)
```

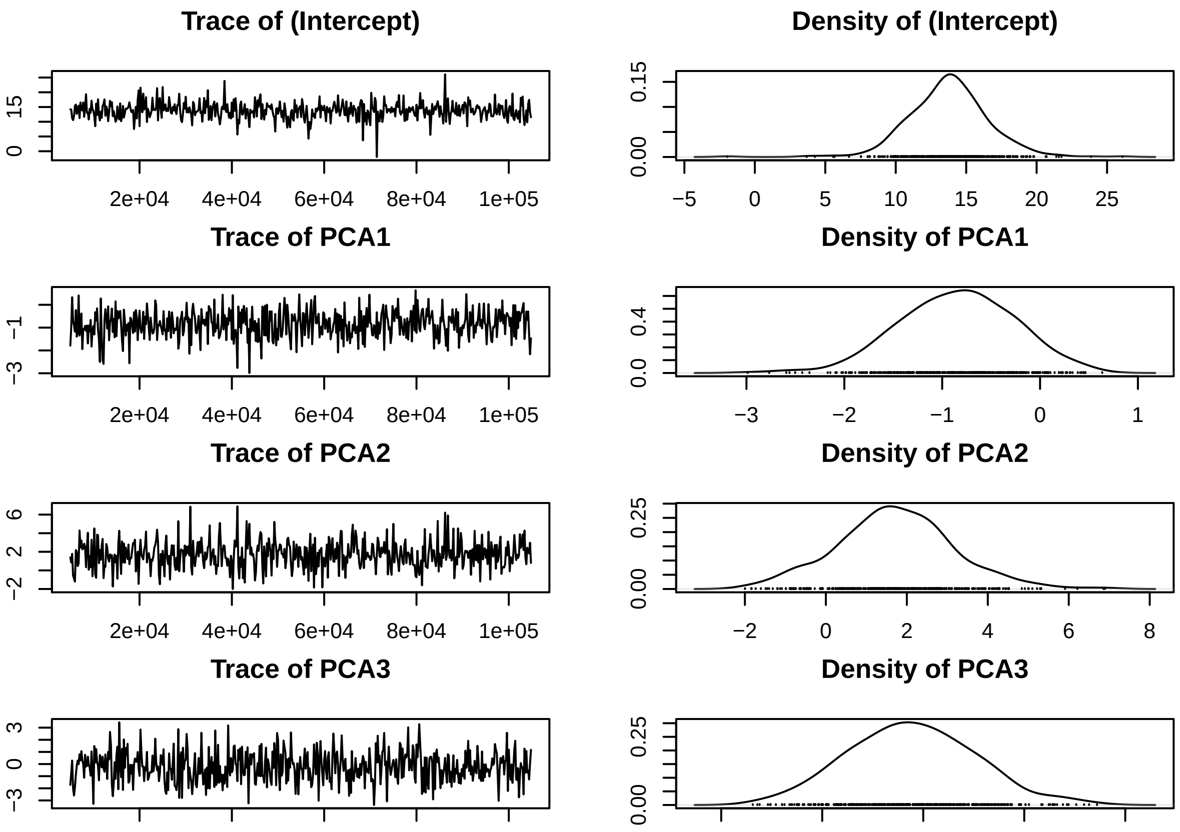

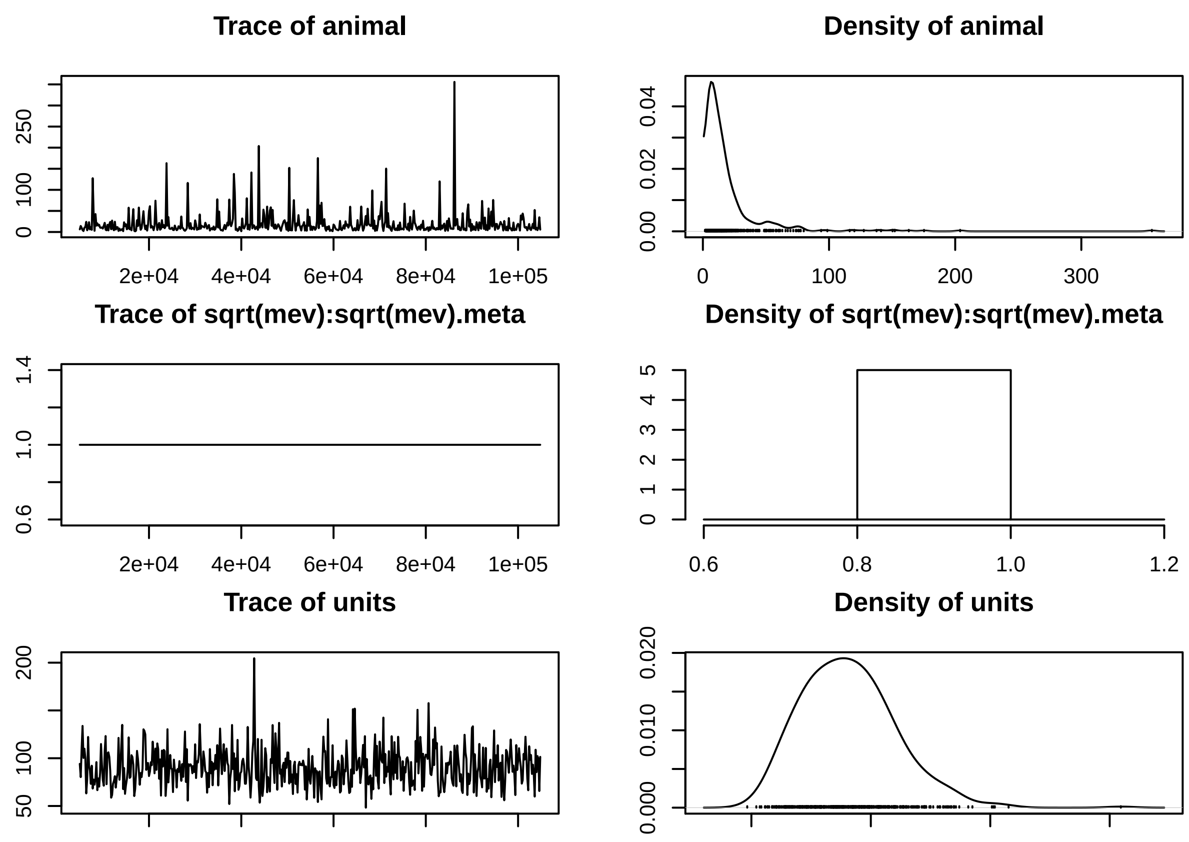

Posterior distribution of PCs fixed effect

```
par(mfrow=c(1,3))
hist(m55.mcmc$Sol[,2], breaks=15, xlab="PCA1 posteriors", main="")
abline(v=posterior.mode((m55.mcmc$Sol[,2])), col="red")
abline(v=median((m55.mcmc$Sol[,2])), col="blue")
abline(v=mean((m55.mcmc$Sol[,2])), col="green")

hist(m55.mcmc$Sol[,3], breaks=15, xlab="PCA2 posteriors", main="")
abline(v=posterior.mode((m55.mcmc$Sol[,3])), col="red")
abline(v=median((m55.mcmc$Sol[,3])), col="blue")
abline(v=mean((m55.mcmc$Sol[,3])), col="green")

hist(m55.mcmc$Sol[,4], breaks=15, xlab="PCA3 posteriors", main="")
abline(v=posterior.mode((m55.mcmc$Sol[,4])), col="red")
abline(v=median((m55.mcmc$Sol[,4])), col="blue")
abline(v=mean((m55.mcmc$Sol[,4])), col="green")
```

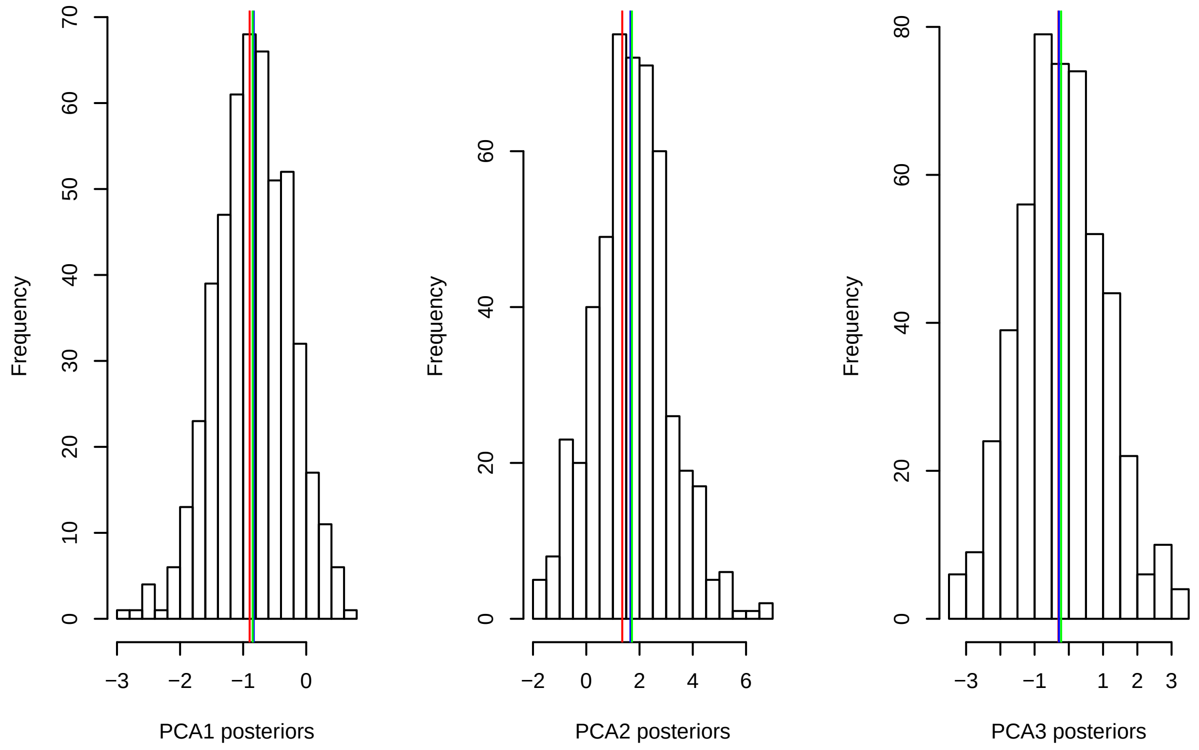

Proportion of Variance explained by phylogeny in Telomeres considering PCA1-2-3 as fixed factors

```
TL_phylogeny<-m55.mcmc$VCV[,'animal']/(m55.mcmc$VCV[,'animal']+m55.mcmc$VCV[,'units']+m55.mcmc$VCV[,"sq

posterior.mode(TL_phylogeny)
```

```
##     var1
## 0.050027
```

```
effectiveSize(TL_phylogeny)
```

```
## var1
##  500
```

```
HPDinterval(TL_phylogeny)
```

```
##           lower    upper
## var1 0.01601154 0.440403
## attr(,"Probability")
## [1] 0.95
```

```r
hist(TL_phylogeny, breaks=30)
abline(v=posterior.mode((TL_phylogeny)), col="red")
abline(v=median((TL_phylogeny)), col="blue")
abline(v=mean((TL_phylogeny)), col="green")
```

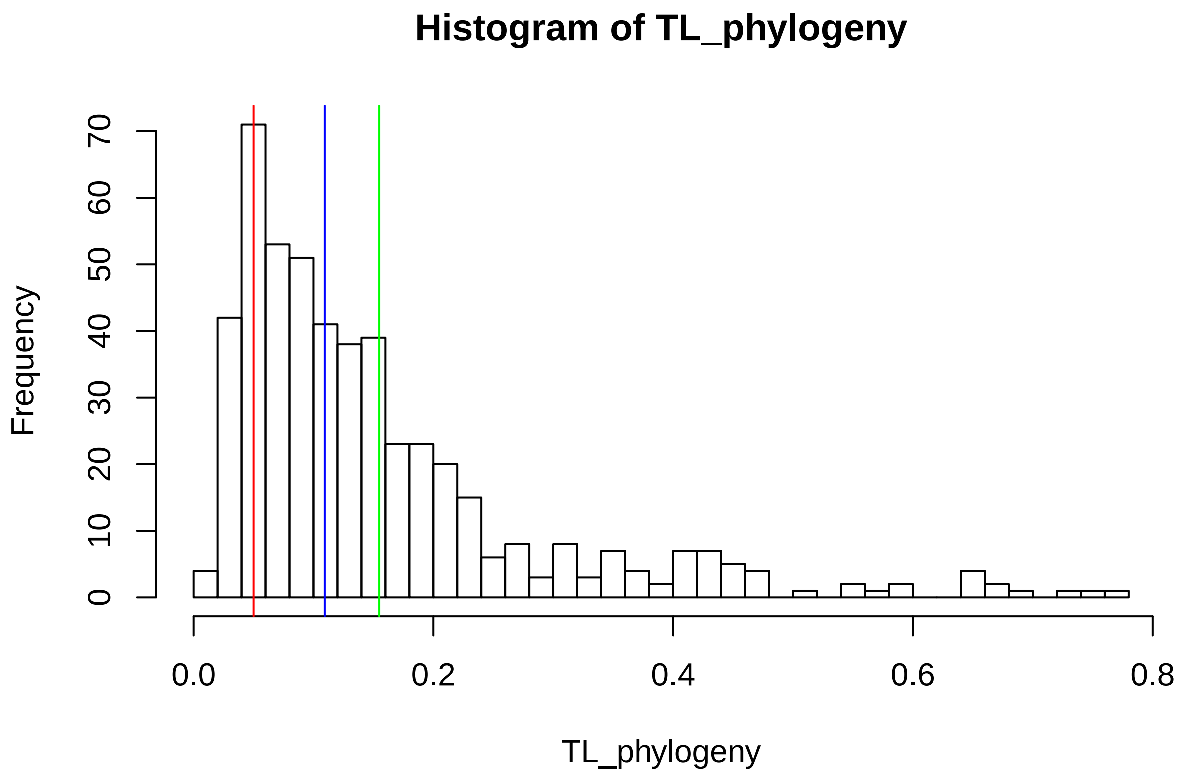


```r
par(mar=c(1,4,4,1))
plot(m55.mcmc)
```

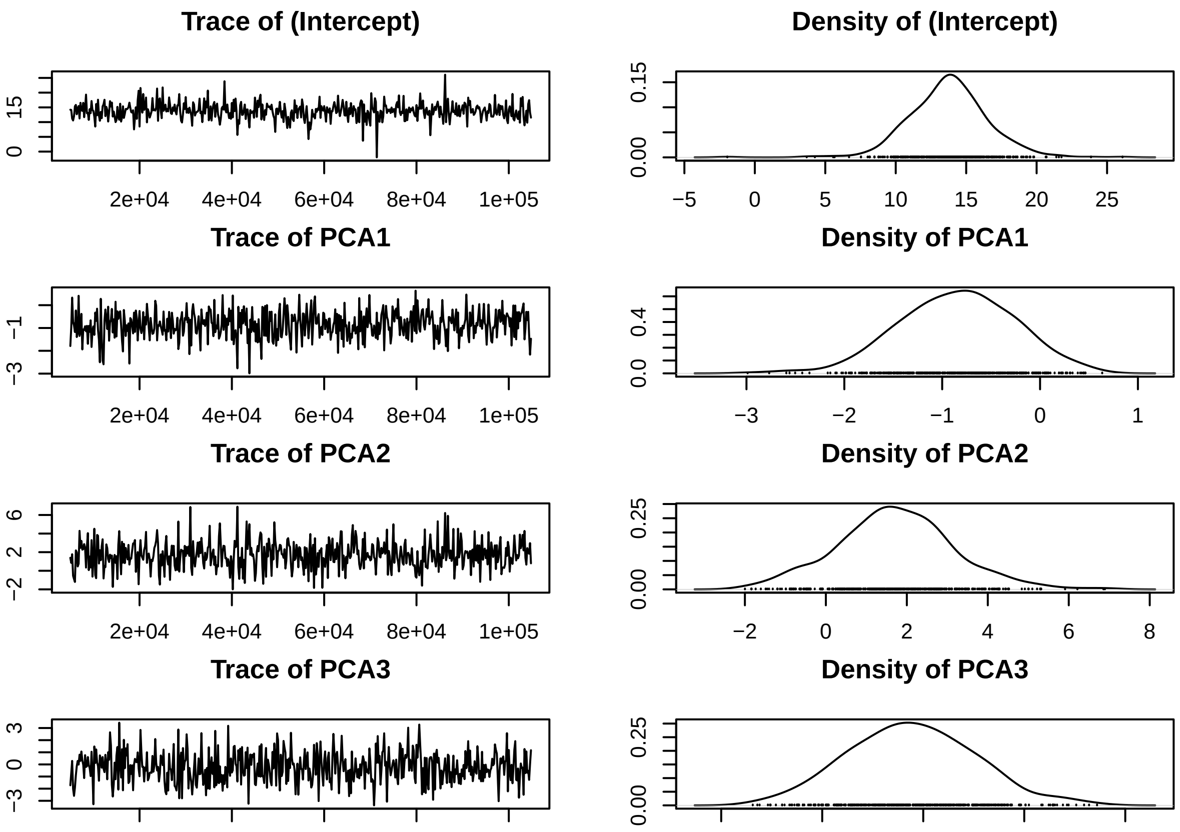
Trace of (Intercept)
Density of (Intercept)
Trace of PCA1
Density of PCA1
Trace of PCA2
Density of PCA2
Trace of PCA3
Density of PCA3

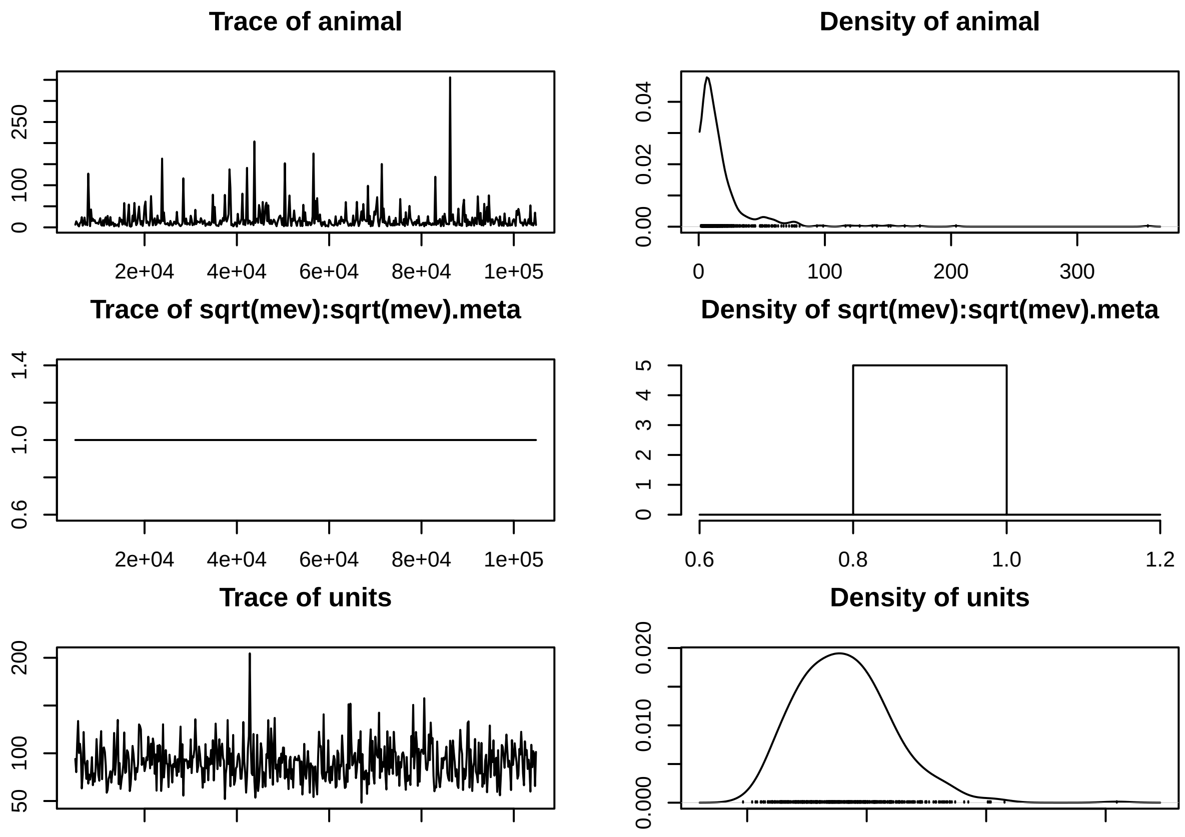


```r
p<-ggplot(Bird.data, aes(x=Bird.data$PCA1, y=Bird.data$Mean.telomere.adult)) +
  geom_point(size=2)+scale_color_grey()+
  theme_classic()+
  geom_smooth(method=lm,linetype="dashed",color="black",fill="grey")
p+labs(x="PCA1",y="Adult TL (bp)")
```

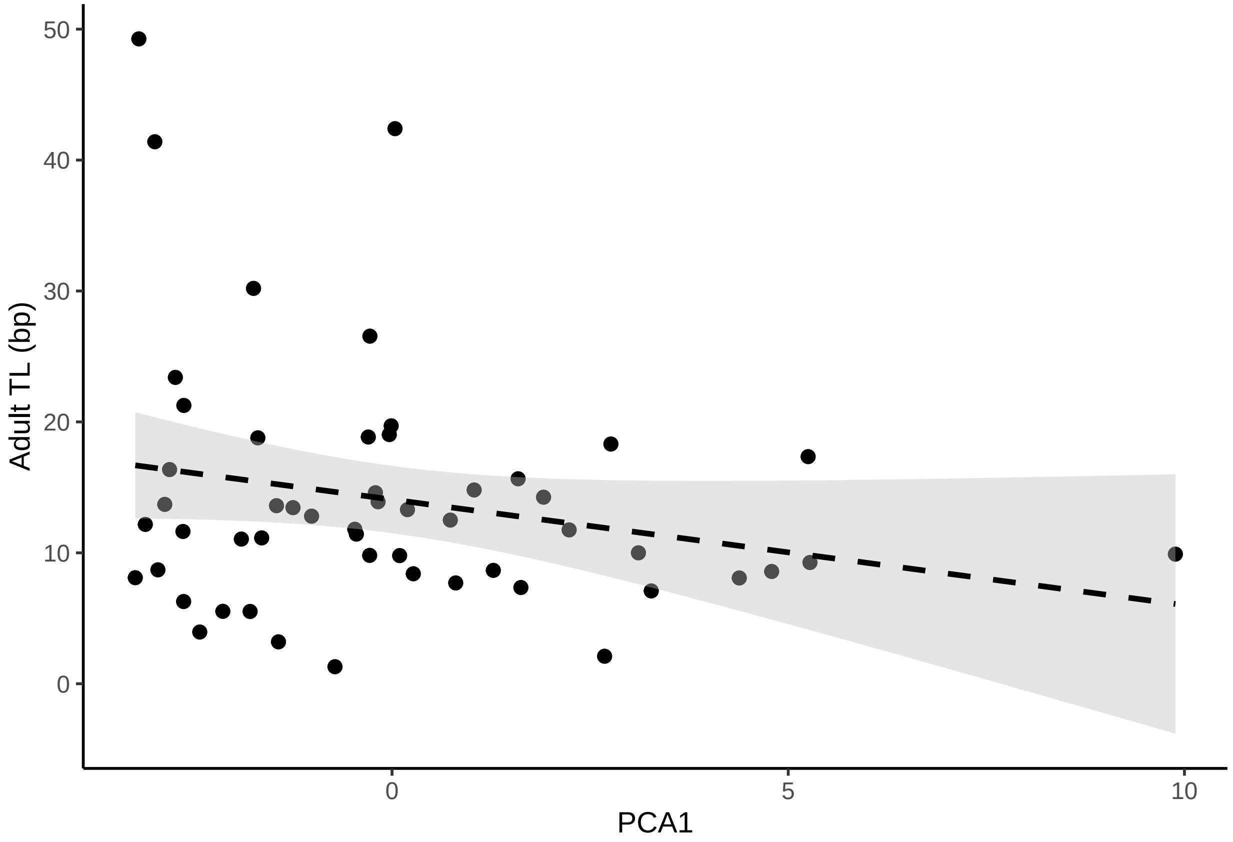


```
p<-ggplot(Bird.data, aes(x=Bird.data$PCA2, y=Bird.data$Mean.telomere.adult))+
  geom_point(size=2)+scale_color_grey()+
  theme_classic()+
  geom_smooth(method=lm,linetype="dashed",color="black",fill="grey")
p+labs(x="PCA2",y="Adult TL (bp)")
```

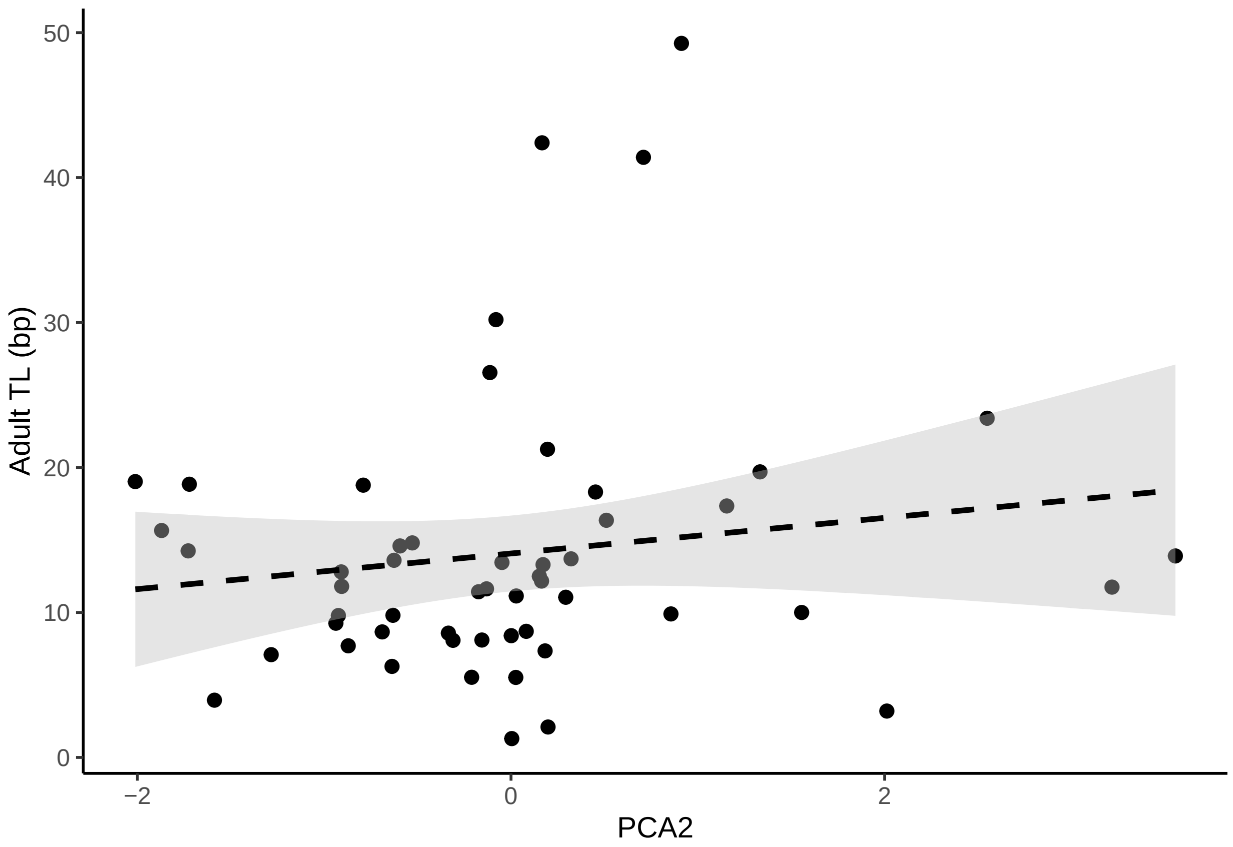


```
p<-ggplot(Bird.data, aes(x=Bird.data$PCA3, y=Bird.data$Mean.telomere.adult))+
  geom_point(size=2)+scale_color_grey()+
  theme_classic()+
  geom_smooth(method=lm,linetype="dashed",color="black",fill="grey")
p+labs(x="PCA3",y="Adult TL (bp)")
```

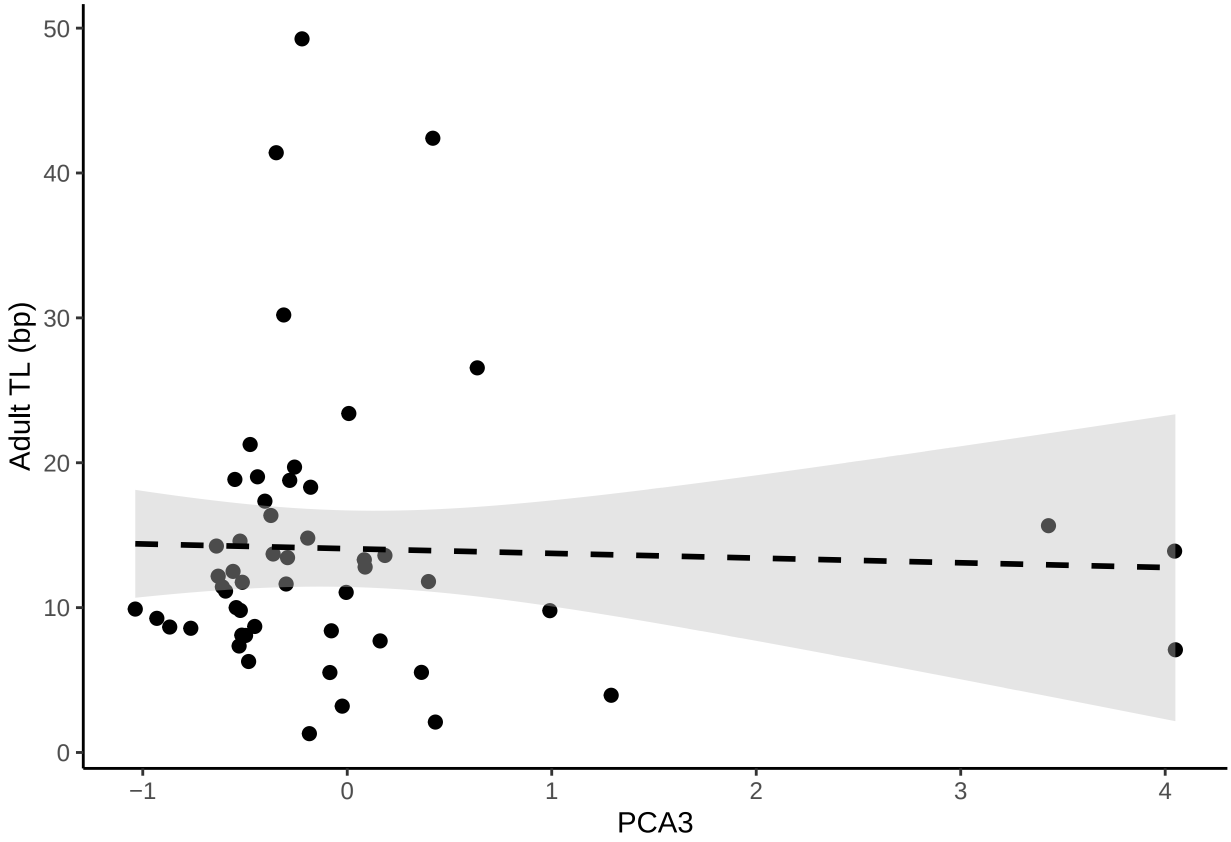

#By orders

```
p<-ggplot(Bird.data, aes(x=Bird.data$PCA3, y=Bird.data$Mean.telomere.adult, color=Bird.data$Order))+
  geom_point(size=2)
p+labs(x="PCA3",y="Adult TL (bp)")
```

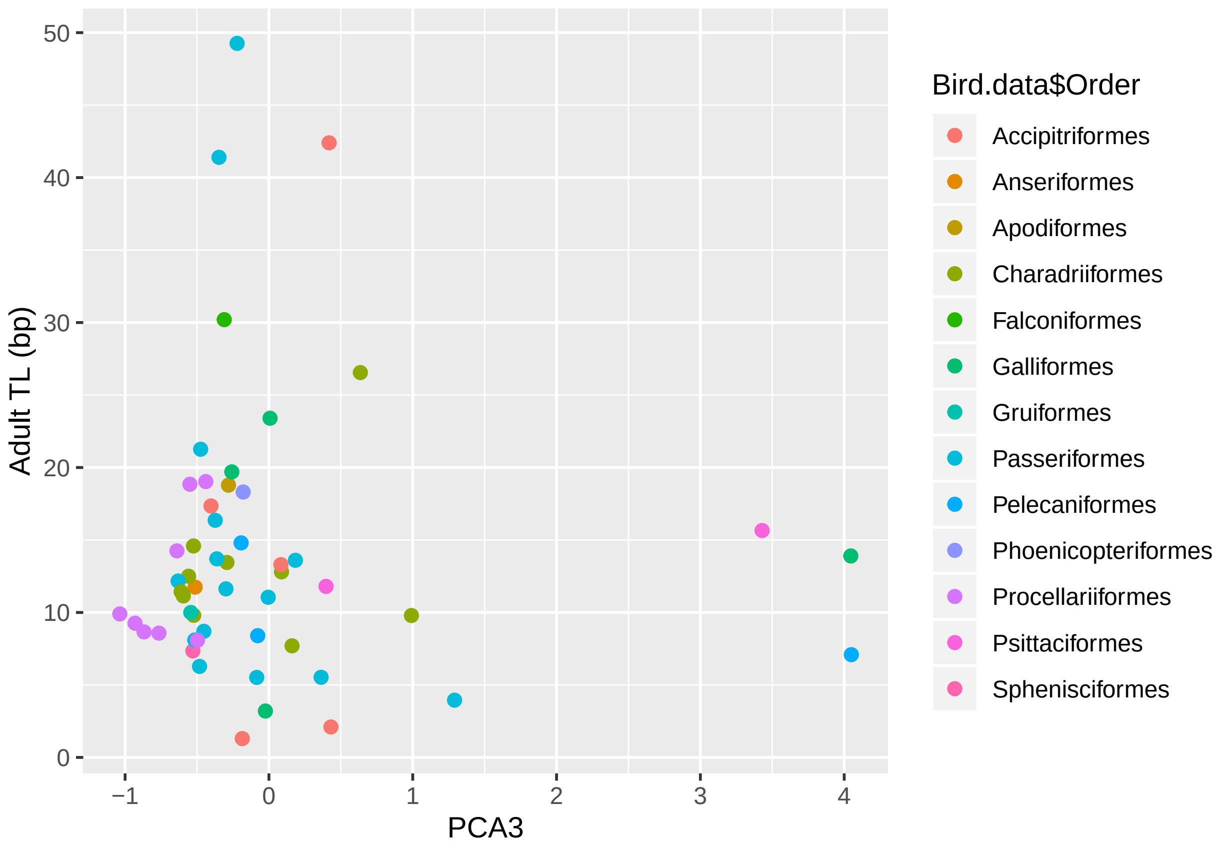


```
p<-ggplot(Bird.data, aes(x=Bird.data$PCA2, y=Bird.data$Mean.telomere.adult, color=Bird.data$Order))+
  geom_point(size=2)
p+labs(x="PCA2",y="Adult TL (bp)")
```

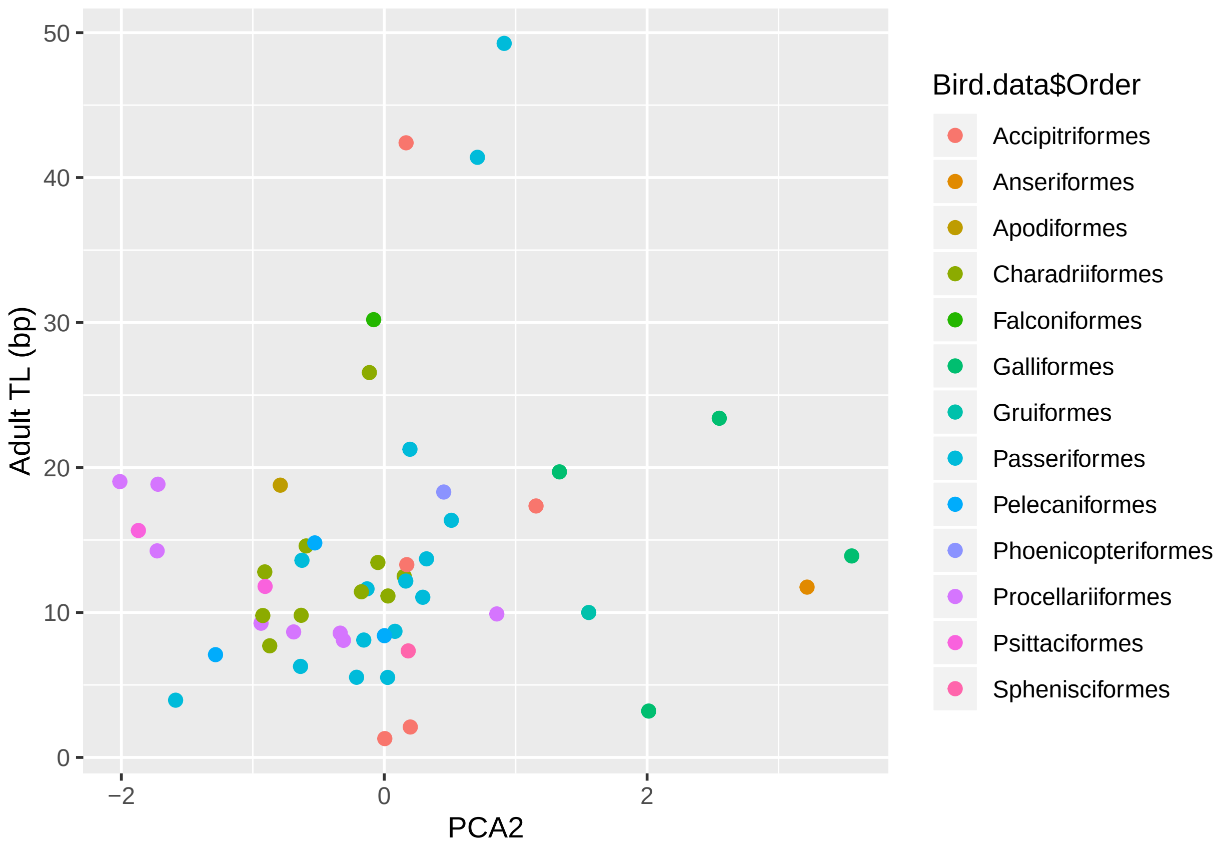


```r
p<-ggplot(Bird.data, aes(x=Bird.data$PCA1, y=Bird.data$Mean.telomere.adult, color=Bird.data$Order))+
  geom_point(size=2)
p+labs(x="PCA1",y="Adult TL (bp)")
```

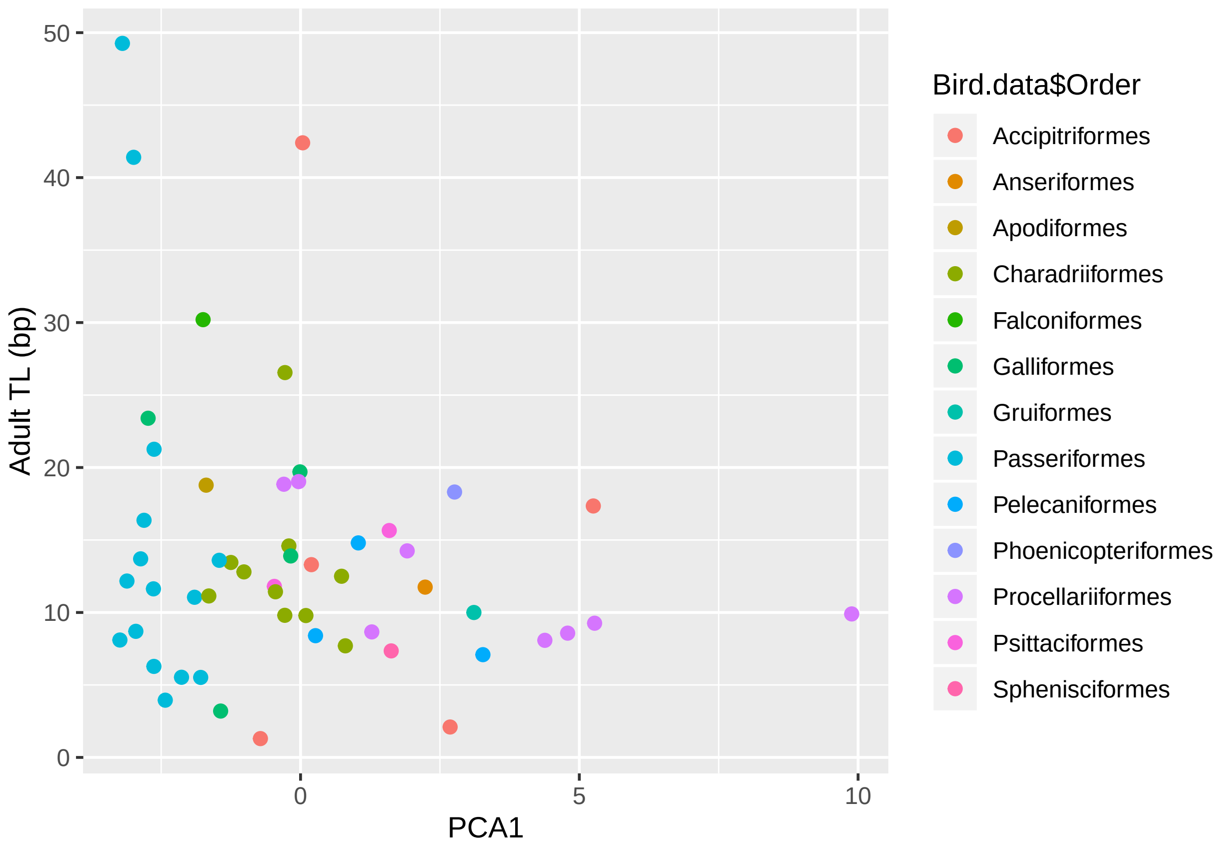

Unadjusted Pearson's correlation

```
cor.test(Bird.data$Mean.telomere.adult, Bird.data$PCA1, alternative = c("two.sided"), method = c("pears
```

```
##
##  Pearson's product-moment correlation
##
## data:  Bird.data$Mean.telomere.adult and Bird.data$PCA1
## t = -1.6733, df = 51, p-value = 0.1004
## alternative hypothesis: true correlation is not equal to 0
## 95 percent confidence interval:
##  -0.4694718  0.0449387
## sample estimates:
##        cor
## -0.2281261
```

```
cor.test(Bird.data$Mean.telomere.adult, Bird.data$PCA2, alternative = c("two.sided"), method = c("pears
```

```
##
##  Pearson's product-moment correlation
##
## data:  Bird.data$Mean.telomere.adult and Bird.data$PCA2
```

```
## t = 1.061, df = 51, p-value = 0.2937
## alternative hypothesis: true correlation is not equal to 0
## 95 percent confidence interval:
##  -0.1284398  0.4013091
## sample estimates:
##       cor
## 0.1469558
```

```r
cor.test(Bird.data$Mean.telomere.adult, Bird.data$PCA3, alternative = c("two.sided"), method = c("pears
```

```
##
##  Pearson's product-moment correlation
##
## data:  Bird.data$Mean.telomere.adult and Bird.data$PCA3
## t = -0.25744, df = 51, p-value = 0.7979
## alternative hypothesis: true correlation is not equal to 0
## 95 percent confidence interval:
##  -0.3033649  0.2365723
## sample estimates:
##         cor
## -0.03602509
```

#By orders #Procellariiformes

```r
proc<-Bird.data[Bird.data$Order=="Procellariiformes",]
cor.test(proc$Mean.telomere.adult, proc$PCA1, alternative = c("two.sided"), method = c("pearson"))
```

```
##
##  Pearson's product-moment correlation
##
## data:  proc$Mean.telomere.adult and proc$PCA1
## t = -2.1593, df = 6, p-value = 0.07415
## alternative hypothesis: true correlation is not equal to 0
## 95 percent confidence interval:
##  -0.93176162  0.08127514
## sample estimates:
##        cor
## -0.6612703
```

```r
cor.test(proc$Mean.telomere.adult, proc$PCA2, alternative = c("two.sided"), method = c("pearson"))
```

```
##
##  Pearson's product-moment correlation
##
## data:  proc$Mean.telomere.adult and proc$PCA2
## t = -2.933, df = 6, p-value = 0.02619
## alternative hypothesis: true correlation is not equal to 0
## 95 percent confidence interval:
##  -0.9554447 -0.1369107
## sample estimates:
##       cor
## -0.767534
```

```
cor.test(proc$Mean.telomere.adult, proc$PCA3, alternative = c("two.sided"), method = c("pearson"))
```

```
##
##  Pearson's product-moment correlation
##
## data:  proc$Mean.telomere.adult and proc$PCA3
## t = 1.8923, df = 6, p-value = 0.1073
## alternative hypothesis: true correlation is not equal to 0
## 95 percent confidence interval:
##  -0.1639607  0.9197791
## sample estimates:
##       cor
## 0.6113464
```

#Charadriiformes

```
cha<-Bird.data[Bird.data$Order=="Charadriiformes",]
cor.test(cha$Mean.telomere.adult, cha$PCA1, alternative = c("two.sided"), method = c("pearson"))
```

```
##
##  Pearson's product-moment correlation
##
## data:  cha$Mean.telomere.adult and cha$PCA1
## t = -0.37244, df = 8, p-value = 0.7192
## alternative hypothesis: true correlation is not equal to 0
## 95 percent confidence interval:
##  -0.7024379  0.5437728
## sample estimates:
##        cor
## -0.1305506
```

```
cor.test(cha$Mean.telomere.adult, cha$PCA2, alternative = c("two.sided"), method = c("pearson"))
```

```
##
##  Pearson's product-moment correlation
##
## data:  cha$Mean.telomere.adult and cha$PCA2
## t = 1.1414, df = 8, p-value = 0.2867
## alternative hypothesis: true correlation is not equal to 0
## 95 percent confidence interval:
##  -0.3341408  0.8124216
## sample estimates:
##       cor
## 0.3742141
```

```
cor.test(cha$Mean.telomere.adult, cha$PCA3, alternative = c("two.sided"), method = c("pearson"))
```

```
##
##  Pearson's product-moment correlation
##
```

```
## data:  cha$Mean.telomere.adult and cha$PCA3
## t = 0.84052, df = 8, p-value = 0.425
## alternative hypothesis: true correlation is not equal to 0
## 95 percent confidence interval:
##  -0.4201188  0.7754109
## sample estimates:
##       cor
## 0.2848573
```

#Passeriformes

```
pas<-Bird.data[Bird.data$Order=="Passeriformes",]
cor.test(pas$Mean.telomere.adult, pas$PCA1, alternative = c("two.sided"), method = c("pearson"))
```

```
##
##  Pearson's product-moment correlation
##
## data:  pas$Mean.telomere.adult and pas$PCA1
## t = -1.6166, df = 13, p-value = 0.13
## alternative hypothesis: true correlation is not equal to 0
## 95 percent confidence interval:
##  -0.7617435  0.1304818
## sample estimates:
##        cor
## -0.4091275
```

```
cor.test(pas$Mean.telomere.adult, pas$PCA2, alternative = c("two.sided"), method = c("pearson"))
```

```
##
##  Pearson's product-moment correlation
##
## data:  pas$Mean.telomere.adult and pas$PCA2
## t = 3.3425, df = 13, p-value = 0.005297
## alternative hypothesis: true correlation is not equal to 0
## 95 percent confidence interval:
##  0.2571296 0.8841828
## sample estimates:
##       cor
## 0.6798447
```

```
cor.test(pas$Mean.telomere.adult, pas$PCA3, alternative = c("two.sided"), method = c("pearson"))
```

```
##
##  Pearson's product-moment correlation
##
## data:  pas$Mean.telomere.adult and pas$PCA3
## t = -0.95629, df = 13, p-value = 0.3564
## alternative hypothesis: true correlation is not equal to 0
## 95 percent confidence interval:
##  -0.6794033  0.2945861
## sample estimates:
##        cor
## -0.2563634
```

#Multivariate phylogenetic analysis

```
### defininf priors
phen.var<-apply(Bird.data[,c("Mean.telomere.adult","PCA1","PCA2", "PCA3")],2,function(m)
  var(m,na.rm=T))

#phenotypic variance divided 80% residual and 20% between-subject as prior, uninformative
prior3 <- list(R=list(V=diag(4)*phen.var*0.8, nu=2),
              G=list(G1=list(V=diag(4)*0.2*phen.var, nu=2)))

## Multivariate Models
m112.mcmc <-  MCMCglmm(cbind(Mean.telomere.adult,PCA1,PCA2,PCA3) ~ trait-1,

                      random = ~us(trait):animal,
                      rcov = ~us(trait):units,
                      mev=Bird.data$mev,
                      data = Bird.data,
                      family=c("gaussian", "gaussian", "gaussian", "gaussian"),
                      pedigree = Bird.phylo,
                      verbose=T,
                      prior=prior3,
                      nitt=105000,
                      thin = 50, burnin = 10000, pr = TRUE, pl = TRUE,  DIC  = TRUE)
```

```
summary(m112.mcmc)
```

```
##
##  Iterations = 10001:104951
##  Thinning interval  = 50
##  Sample size  = 1900
##
##  DIC: 838.8021
##
##  G-structure:  ~us(trait):animal
##
##                                                      post.mean  l-95% CI
## traitMean.telomere.adult:traitMean.telomere.adult.animal   35.8472   4.03158
## traitPCA1:traitMean.telomere.adult.animal                  -6.4779 -31.98775
## traitPCA2:traitMean.telomere.adult.animal                   0.1728  -8.73642
## traitPCA3:traitMean.telomere.adult.animal                  -0.1311  -6.57440
## traitMean.telomere.adult:traitPCA1.animal                  -6.4779 -31.98775
## traitPCA1:traitPCA1.animal                                 11.0561   1.92590
## traitPCA2:traitPCA1.animal                                  1.0666  -2.19796
## traitPCA3:traitPCA1.animal                                 -0.5104  -3.44715
## traitMean.telomere.adult:traitPCA2.animal                   0.1728  -8.73642
## traitPCA1:traitPCA2.animal                                  1.0666  -2.19796
## traitPCA2:traitPCA2.animal                                  1.3687   0.20596
## traitPCA3:traitPCA2.animal                                 -0.2359  -1.48830
## traitMean.telomere.adult:traitPCA3.animal                  -0.1311  -6.57440
## traitPCA1:traitPCA3.animal                                 -0.5104  -3.44715
## traitPCA2:traitPCA3.animal                                 -0.2359  -1.48830
## traitPCA3:traitPCA3.animal                                  0.6539   0.03702
##                                                      u-95% CI eff.samp
```

```
## traitMean.telomere.adult:traitMean.telomere.adult.animal 100.3564   1121.3
## traitPCA1:traitMean.telomere.adult.animal                 17.5844    962.9
## traitPCA2:traitMean.telomere.adult.animal                  8.2066    957.7
## traitPCA3:traitMean.telomere.adult.animal                  5.8417    788.8
## traitMean.telomere.adult:traitPCA1.animal                 17.5844    962.9
## traitPCA1:traitPCA1.animal                                23.0722   1556.5
## traitPCA2:traitPCA1.animal                                 5.0851   1103.6
## traitPCA3:traitPCA1.animal                                 2.3544    825.4
## traitMean.telomere.adult:traitPCA2.animal                  8.2066    957.7
## traitPCA1:traitPCA2.animal                                 5.0851   1103.6
## traitPCA2:traitPCA2.animal                                 3.0404   1133.9
## traitPCA3:traitPCA2.animal                                 0.7919    736.7
## traitMean.telomere.adult:traitPCA3.animal                  5.8417    788.8
## traitPCA1:traitPCA3.animal                                 2.3544    825.4
## traitPCA2:traitPCA3.animal                                 0.7919    736.7
## traitPCA3:traitPCA3.animal                                 1.9617    902.0
##
##  R-structure:  ~us(trait):units
##
##                                                         post.mean l-95% CI
## traitMean.telomere.adult:traitMean.telomere.adult.units   95.7379  57.1744
## traitPCA1:traitMean.telomere.adult.units                  -2.1016  -8.7489
## traitPCA2:traitMean.telomere.adult.units                   1.3876  -1.1701
## traitPCA3:traitMean.telomere.adult.units                  -0.6477  -4.0537
## traitMean.telomere.adult:traitPCA1.units                  -2.1016  -8.7489
## traitPCA1:traitPCA1.units                                  2.6126   0.9060
## traitPCA2:traitPCA1.units                                  0.3567  -0.1998
## traitPCA3:traitPCA1.units                                  0.3339  -0.2749
## traitMean.telomere.adult:traitPCA2.units                   1.3876  -1.1701
## traitPCA1:traitPCA2.units                                  0.3567  -0.1998
## traitPCA2:traitPCA2.units                                  0.4909   0.1852
## traitPCA3:traitPCA2.units                                 -0.2816  -0.5935
## traitMean.telomere.adult:traitPCA3.units                  -0.6477  -4.0537
## traitPCA1:traitPCA3.units                                  0.3339  -0.2749
## traitPCA2:traitPCA3.units                                 -0.2816  -0.5935
## traitPCA3:traitPCA3.units                                  0.9012   0.4485
##                                                          u-95% CI eff.samp
## traitMean.telomere.adult:traitMean.telomere.adult.units 137.223264     1739
## traitPCA1:traitMean.telomere.adult.units                  3.374676     1900
## traitPCA2:traitMean.telomere.adult.units                  4.247047     2010
## traitPCA3:traitMean.telomere.adult.units                  2.543228     1786
## traitMean.telomere.adult:traitPCA1.units                  3.374676     1900
## traitPCA1:traitPCA1.units                                 4.335664     1746
## traitPCA2:traitPCA1.units                                 0.988975     1484
## traitPCA3:traitPCA1.units                                 1.007569     1604
## traitMean.telomere.adult:traitPCA2.units                  4.247047     2010
## traitPCA1:traitPCA2.units                                 0.988975     1484
## traitPCA2:traitPCA2.units                                 0.796190     1656
## traitPCA3:traitPCA2.units                                -0.007427     1286
## traitMean.telomere.adult:traitPCA3.units                  2.543228     1786
## traitPCA1:traitPCA3.units                                 1.007569     1604
## traitPCA2:traitPCA3.units                                -0.007427     1286
## traitPCA3:traitPCA3.units                                 1.383284     1588
##
```

```
##  Location effects: cbind(Mean.telomere.adult, PCA1, PCA2, PCA3) ~ trait - 1
##
##                             post.mean l-95% CI u-95% CI eff.samp   pMCMC
## traitMean.telomere.adult  14.10248  7.16382 21.78657     1900 0.00105 **
## traitPCA1                  0.11689 -3.30092  3.90086     1900 0.96000
## traitPCA2                  0.40532 -0.82111  1.77839     1900 0.51579
## traitPCA3                  0.02954 -0.92711  0.99422     1900 0.94421
## ---
## Signif. codes:  0 '***' 0.001 '**' 0.01 '*' 0.05 '.' 0.1 ' ' 1
```

```r
par(mar=c(1,4,4,1))
plot(m112.mcmc)
```

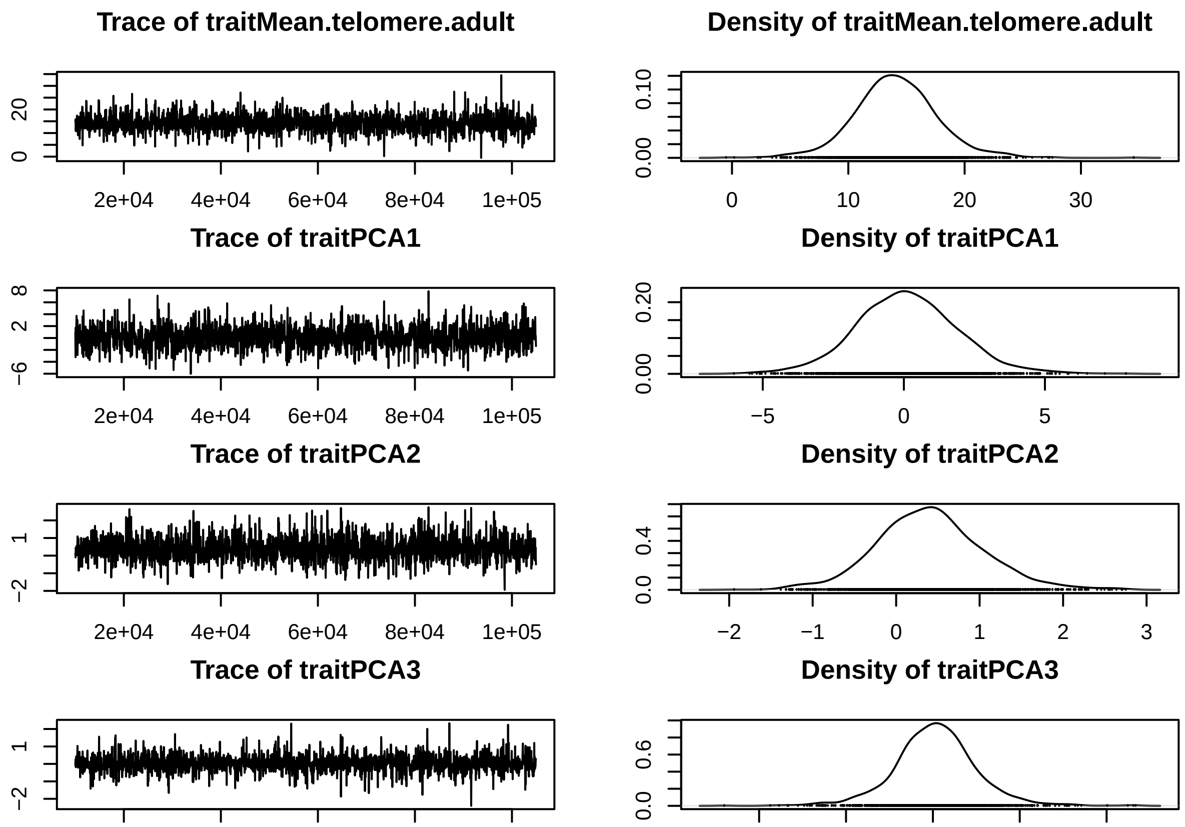

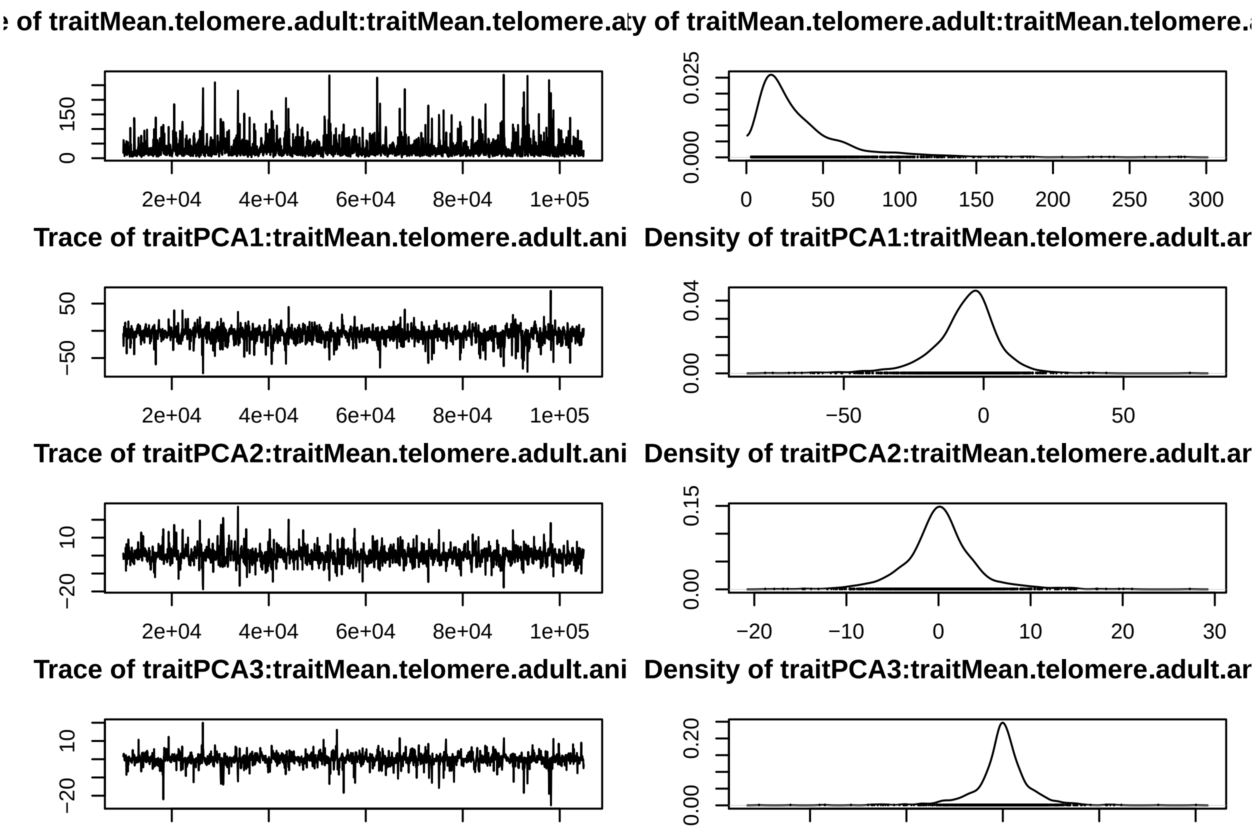

e of traitMean.telomere.adult:traitMean.telomere.a
y of traitMean.telomere.adult:traitMean.telomere.a
0
150
2e+04
4e+04
6e+04
8e+04
1e+05
0.000
0.025
0
50
100
150
200
250
300
Trace of traitPCA1:traitMean.telomere.adult.ani
Density of traitPCA1:traitMean.telomere.adult.ar
−50
50
0.00
0.04
Trace of traitPCA2:traitMean.telomere.adult.ani
Density of traitPCA2:traitMean.telomere.adult.ar
−20
10
0.00
0.15
−20
−10
0
10
20
30
Trace of traitPCA3:traitMean.telomere.adult.ani
Density of traitPCA3:traitMean.telomere.adult.ar
−20
10
0.00
0.20

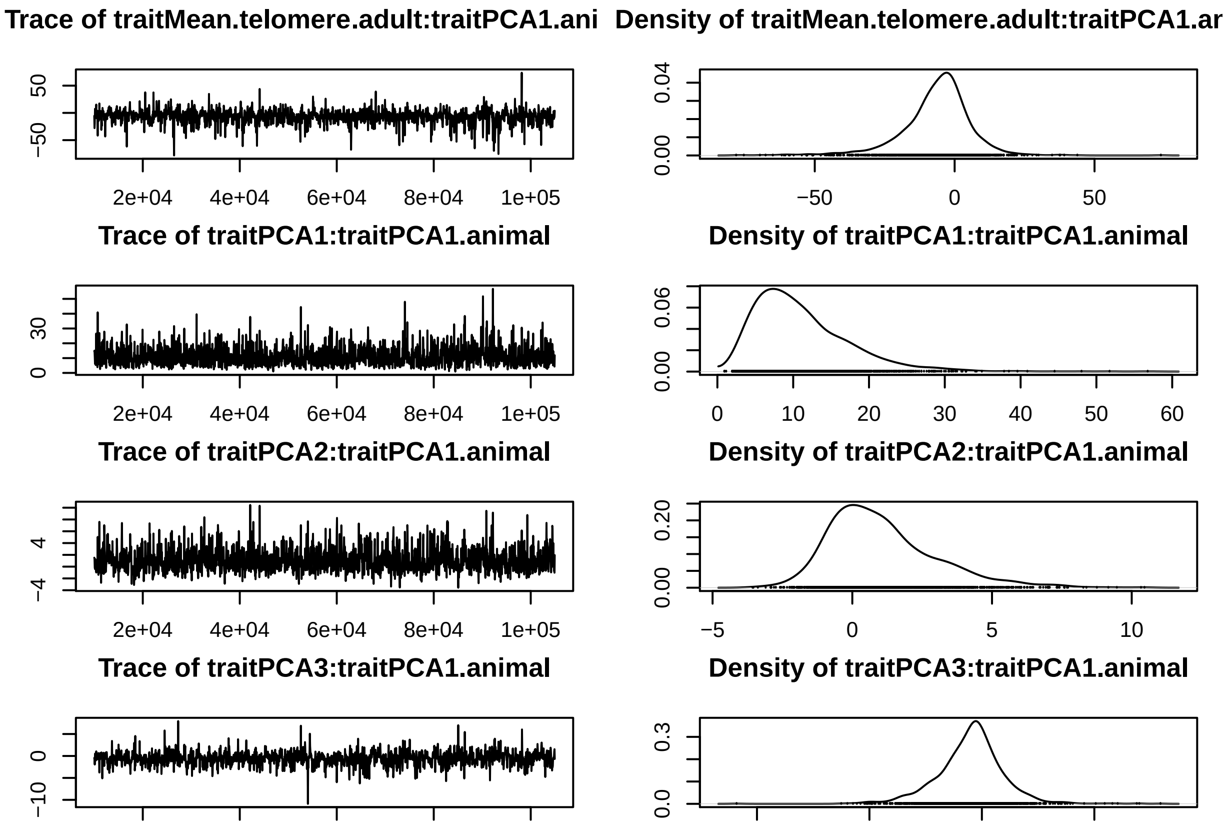
Trace of traitMean.telomere.adult:traitPCA1.ani
Density of traitMean.telomere.adult:traitPCA1.ar
Trace of traitPCA1:traitPCA1.animal
Density of traitPCA1:traitPCA1.animal
Trace of traitPCA2:traitPCA1.animal
Density of traitPCA2:traitPCA1.animal
Trace of traitPCA3:traitPCA1.animal
Density of traitPCA3:traitPCA1.animal
2e+04
4e+04
6e+04
8e+04
1e+05

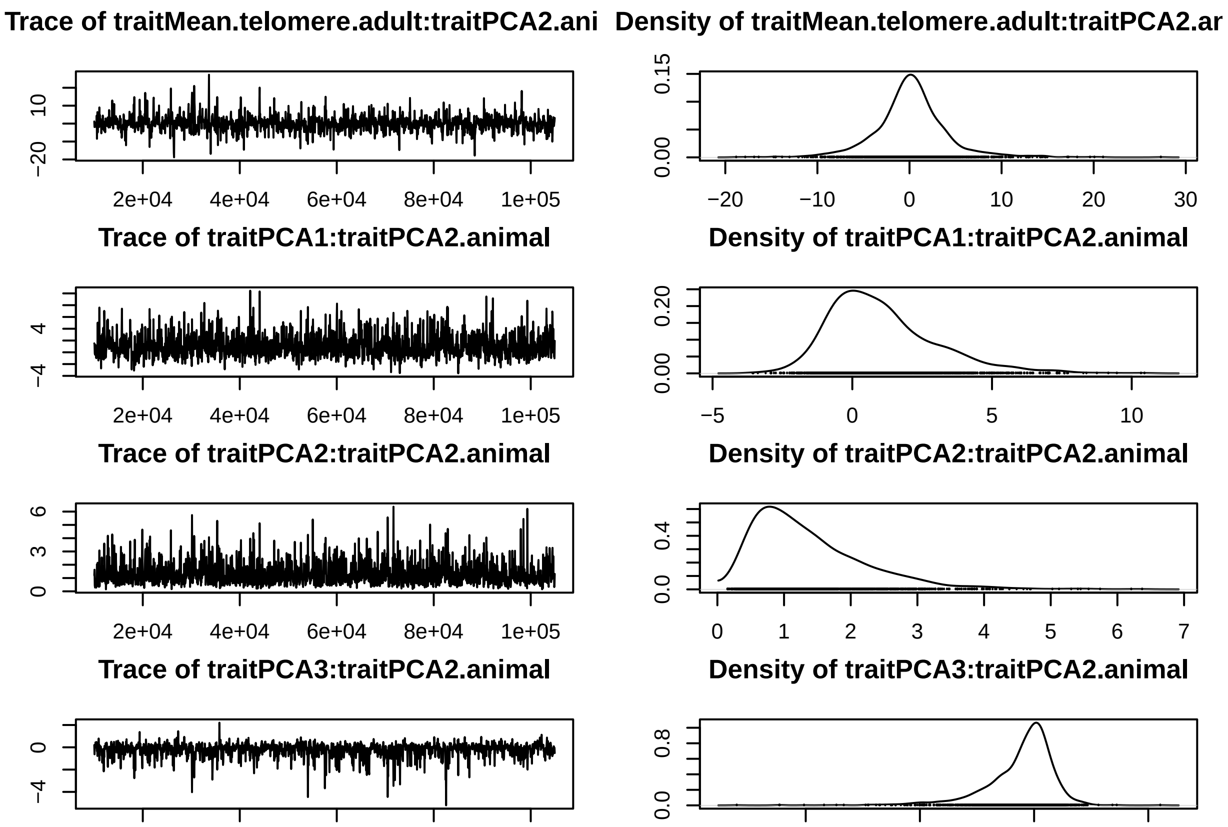
Trace of traitMean.telomere.adult:traitPCA2.ani
Density of traitMean.telomere.adult:traitPCA2.ar
Trace of traitPCA1:traitPCA2.animal
Density of traitPCA1:traitPCA2.animal
Trace of traitPCA2:traitPCA2.animal
Density of traitPCA2:traitPCA2.animal
Trace of traitPCA3:traitPCA2.animal
Density of traitPCA3:traitPCA2.animal

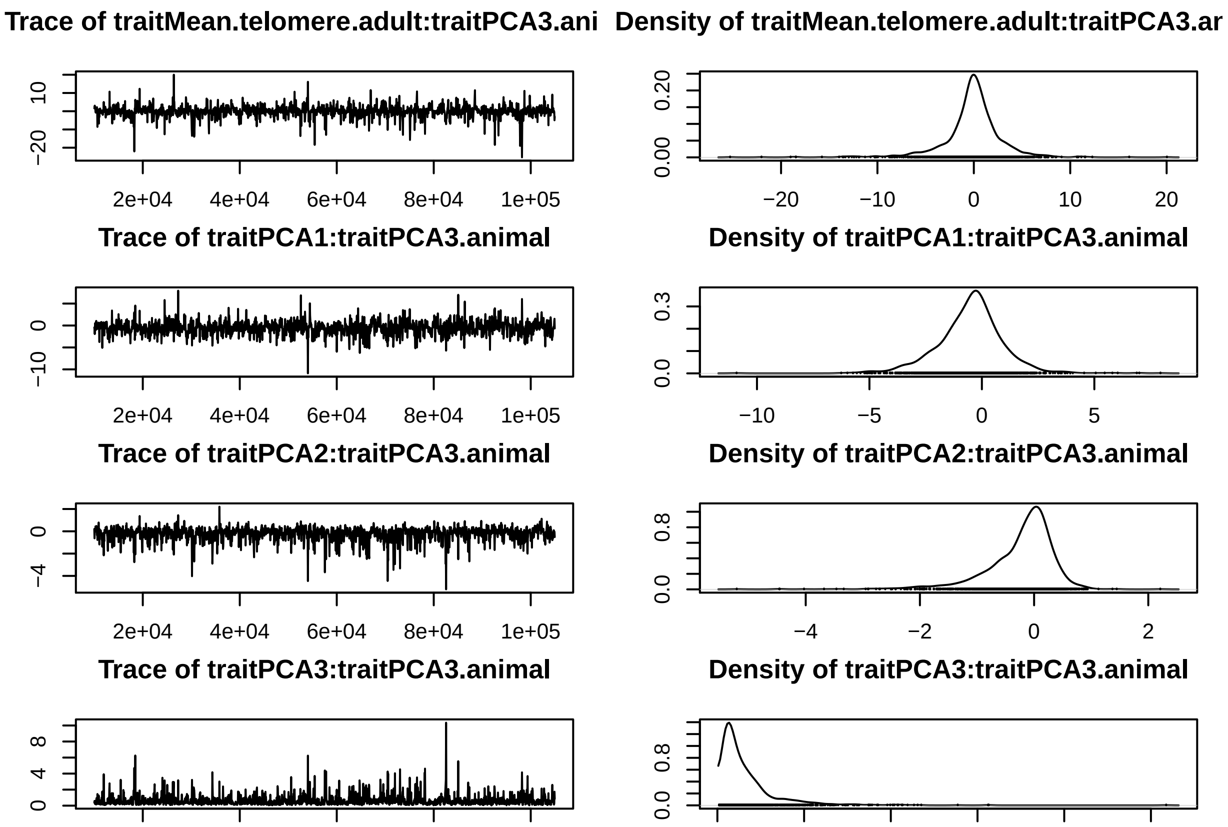

Trace of traitMean.telomere.adult:traitPCA3.ani
Density of traitMean.telomere.adult:traitPCA3.ar
Trace of traitPCA1:traitPCA3.animal
Density of traitPCA1:traitPCA3.animal
Trace of traitPCA2:traitPCA3.animal
Density of traitPCA2:traitPCA3.animal
Trace of traitPCA3:traitPCA3.animal
Density of traitPCA3:traitPCA3.animal
2e+04
4e+04
6e+04
8e+04
1e+05

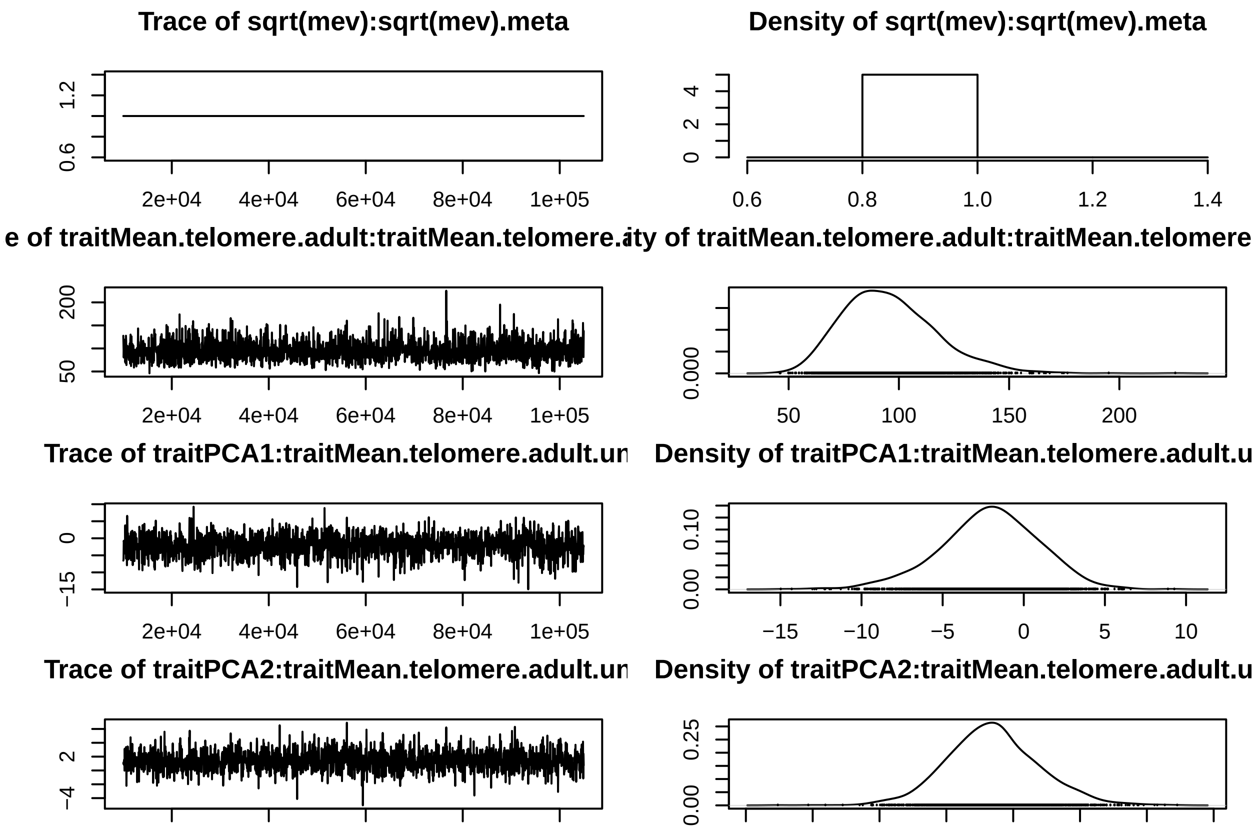
Trace of sqrt(mev):sqrt(mev).meta
Density of sqrt(mev):sqrt(mev).meta
e of traitMean.telomere.adult:traitMean.telomere.a
ity of traitMean.telomere.adult:traitMean.telomere
Trace of traitPCA1:traitMean.telomere.adult.un
Density of traitPCA1:traitMean.telomere.adult.u
Trace of traitPCA2:traitMean.telomere.adult.un
Density of traitPCA2:traitMean.telomere.adult.u
2e+04
4e+04
6e+04
8e+04
1e+05
0.6
1.2
0
2
4
0.6
0.8
1.0
1.2
1.4
50
200
0.000
50
100
150
200
0
−15
0.00
0.10
−15
−10
−5
0
5
10
2
−4
0.00
0.25

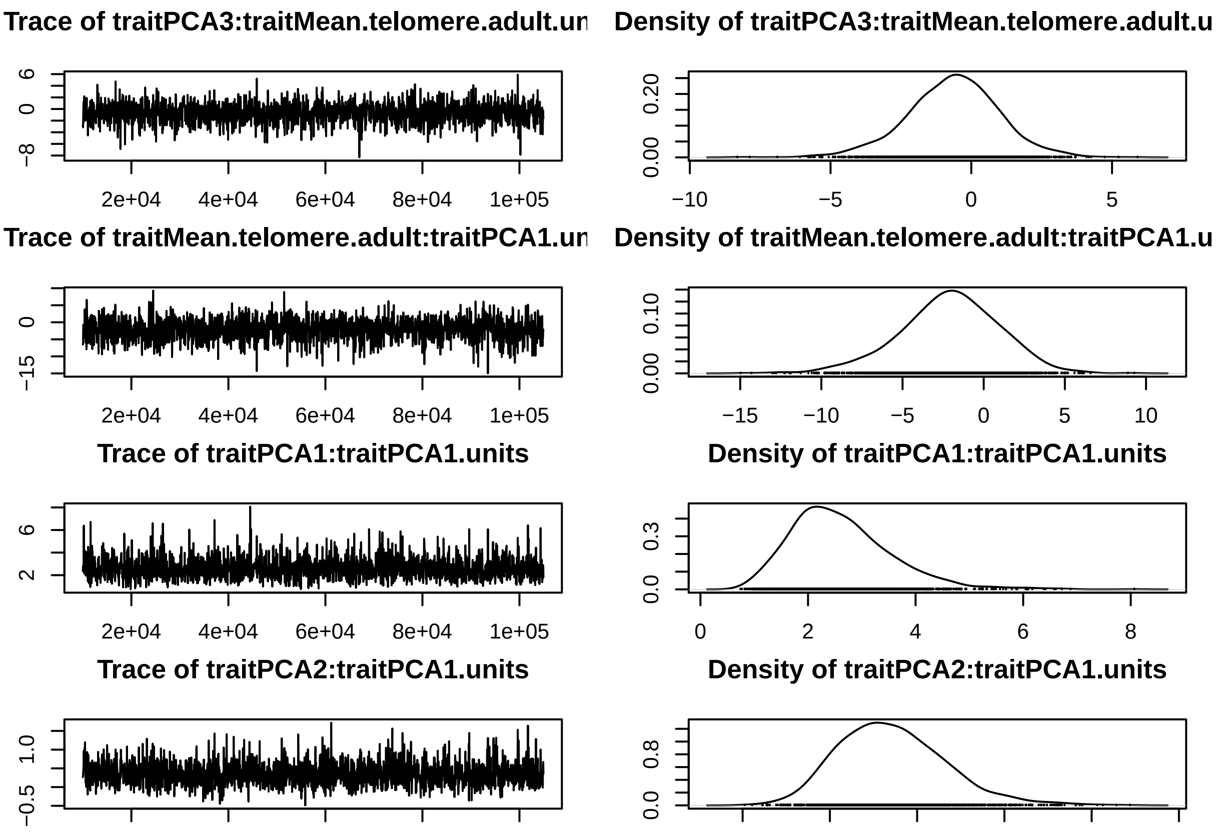

Trace of traitPCA3:traitMean.telomere.adult.un
Density of traitPCA3:traitMean.telomere.adult.u
Trace of traitMean.telomere.adult:traitPCA1.un
Density of traitMean.telomere.adult:traitPCA1.u
Trace of traitPCA1:traitPCA1.units
Density of traitPCA1:traitPCA1.units
Trace of traitPCA2:traitPCA1.units
Density of traitPCA2:traitPCA1.units

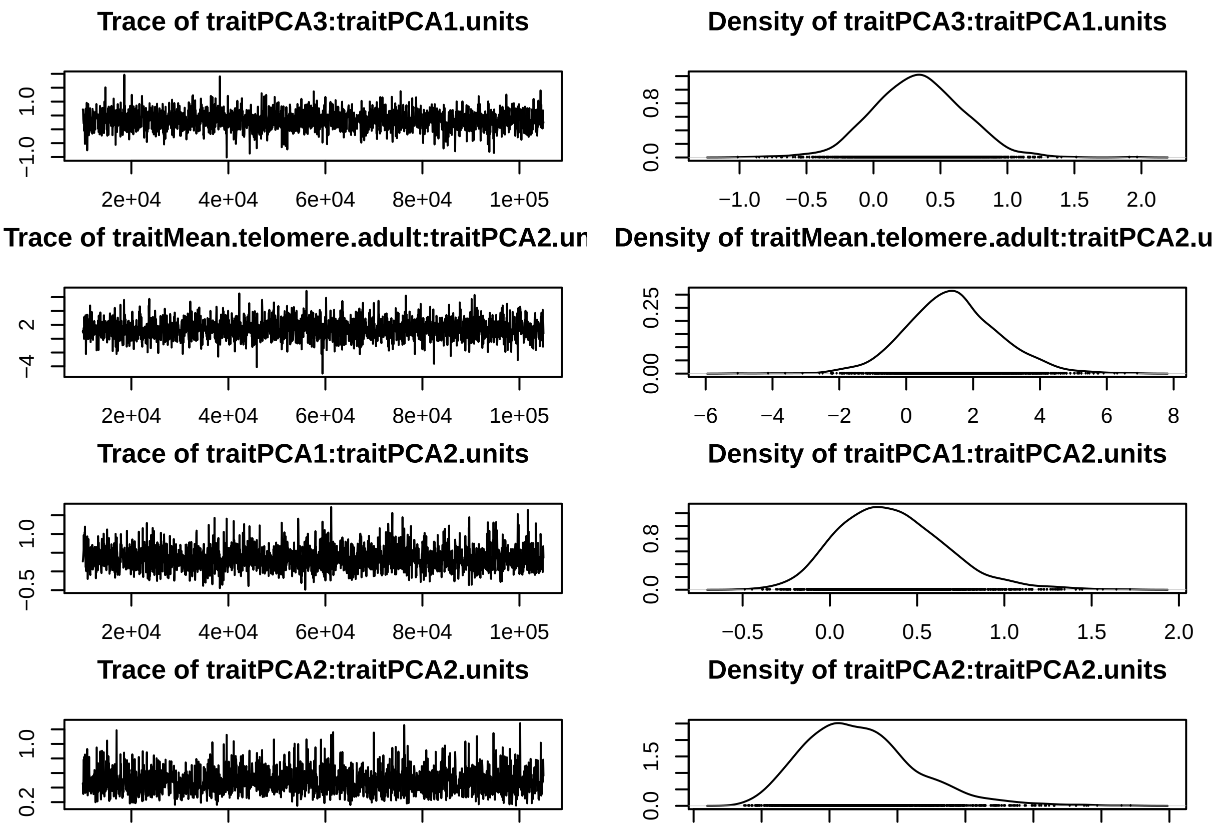
Trace of traitPCA3:traitPCA1.units
Density of traitPCA3:traitPCA1.units
Trace of traitMean.telomere.adult:traitPCA2.un
Density of traitMean.telomere.adult:traitPCA2.u
Trace of traitPCA1:traitPCA2.units
Density of traitPCA1:traitPCA2.units
Trace of traitPCA2:traitPCA2.units
Density of traitPCA2:traitPCA2.units
2e+04
4e+04
6e+04
8e+04
1e+05

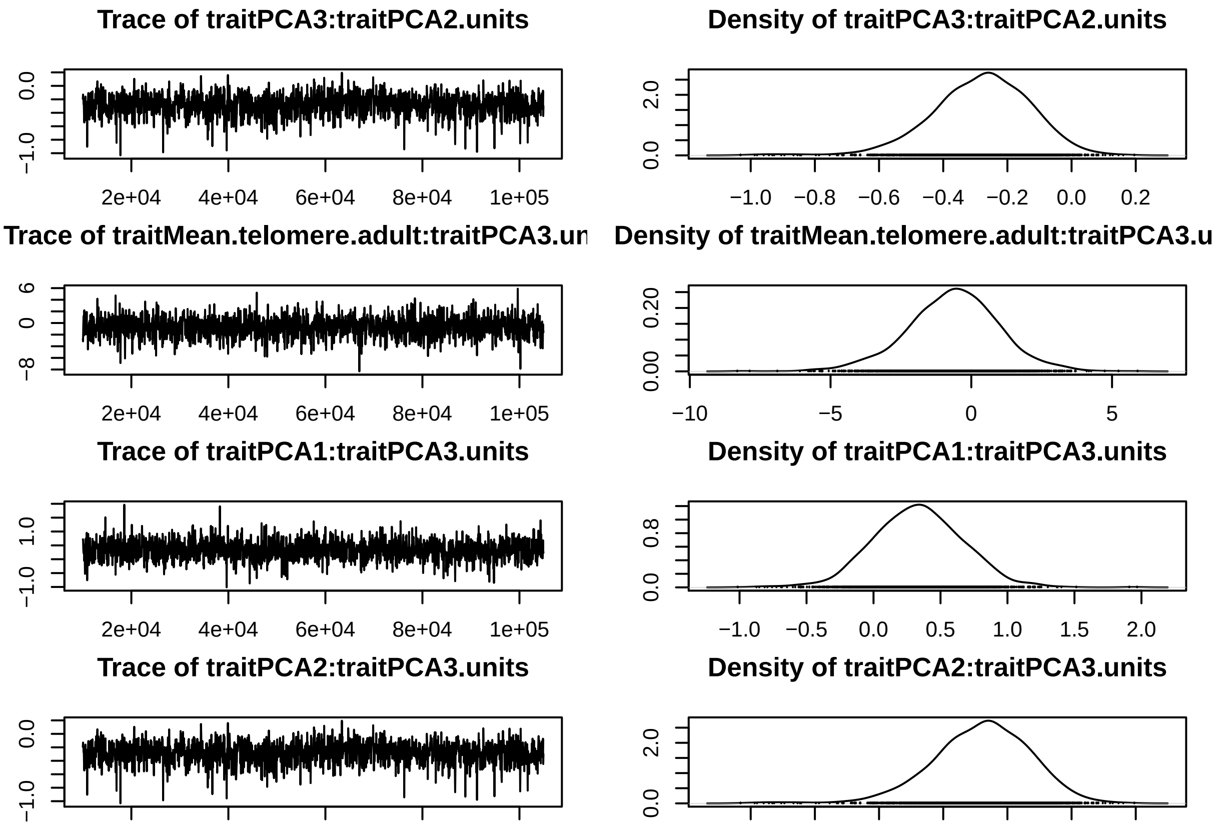
Trace of traitPCA3:traitPCA2.units
Density of traitPCA3:traitPCA2.units
Trace of traitMean.telomere.adult:traitPCA3.un
Density of traitMean.telomere.adult:traitPCA3.u
Trace of traitPCA1:traitPCA3.units
Density of traitPCA1:traitPCA3.units
Trace of traitPCA2:traitPCA3.units
Density of traitPCA2:traitPCA3.units
2e+04
4e+04
6e+04
8e+04
1e+05
−1.0 −0.8 −0.6 −0.4 −0.2 0.0 0.2
−10 −5 0 5
−1.0 −0.5 0.0 0.5 1.0 1.5 2.0

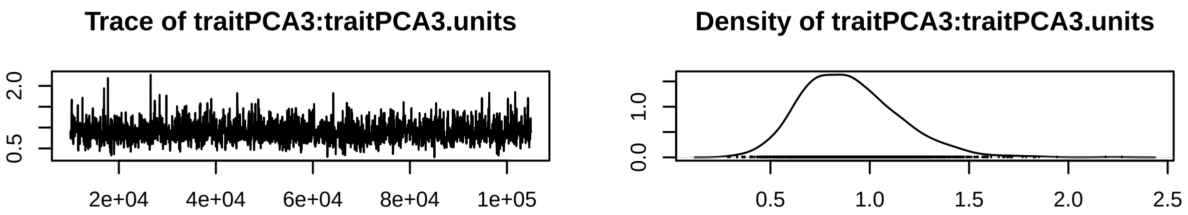


```r
p<-ggplot(Bird.data, aes(x=Bird.data$PCA1, y=Bird.data$Mean.telomere.adult)) +
  geom_point(size=2)+scale_color_grey()+
  theme_classic()+geom_smooth(method=lm,linetype="dashed",color="black",fill="grey")
p+labs(x="PCA1",y="Adult TL (bp)")
```

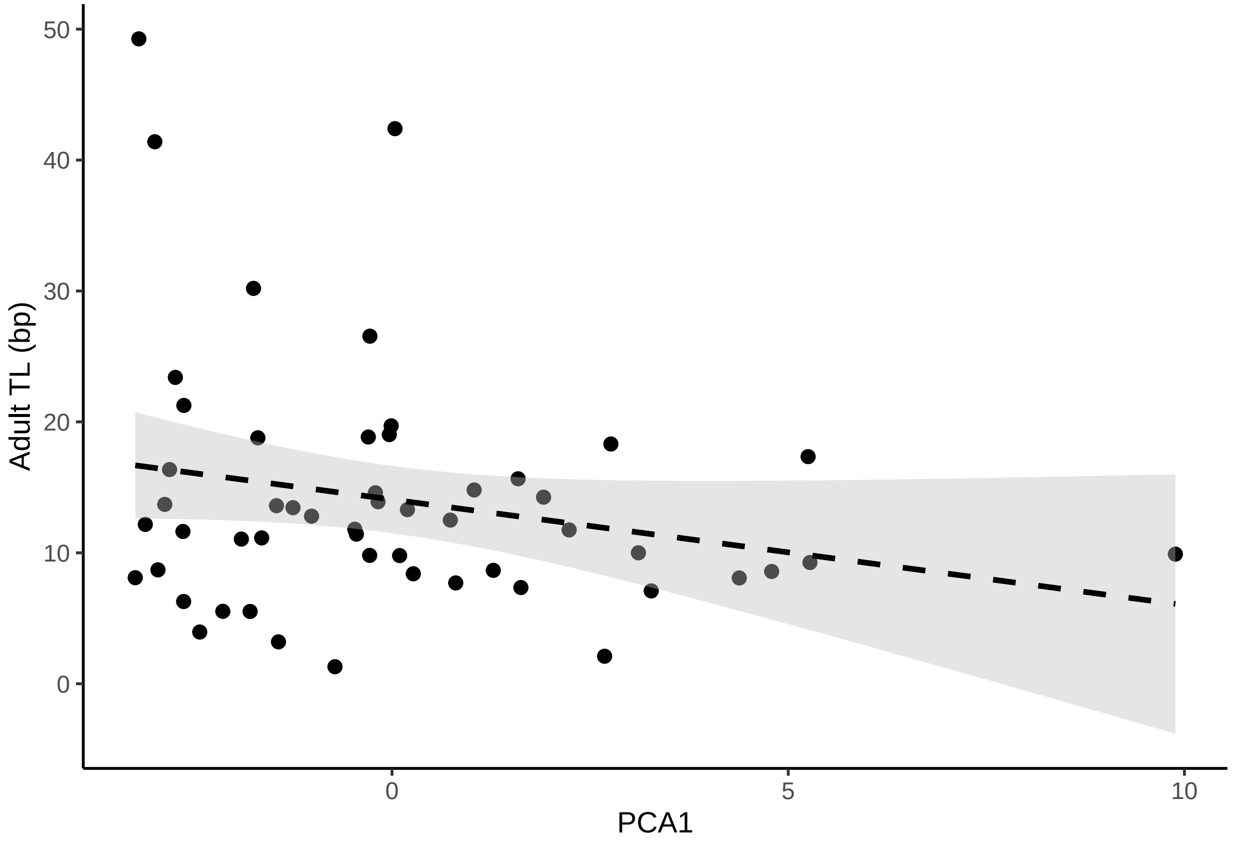


```
p<-ggplot(Bird.data, aes(x=Bird.data$PCA2, y=Bird.data$Mean.telomere.adult)) +
  geom_point(size=2)+
  scale_color_grey()+
  theme_classic()+
  geom_smooth(method=lm,linetype="dashed",color="black",fill="grey")
p+labs(x="PCA2",y="Adult TL (bp)")
```

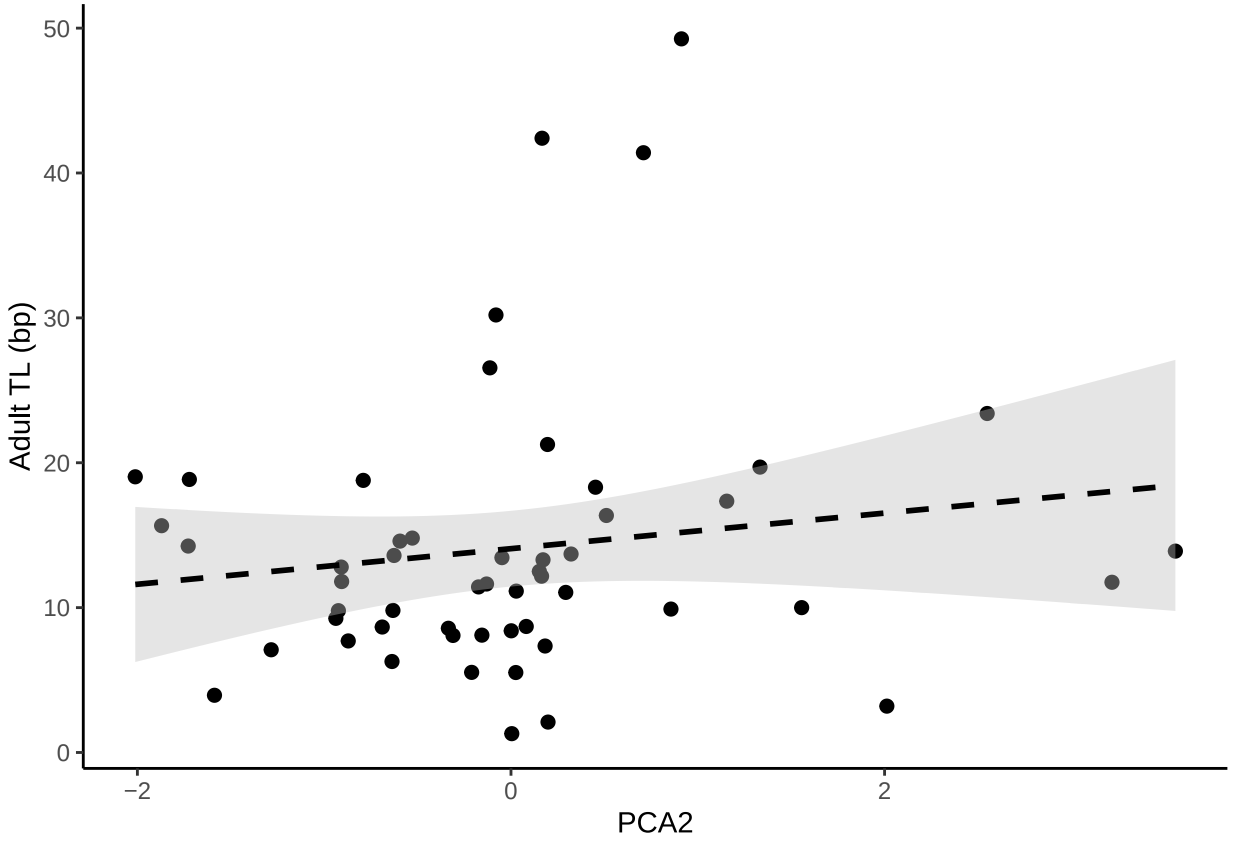


```
p<-ggplot(Bird.data, aes(x=Bird.data$PCA3, y=Bird.data$Mean.telomere.adult)) +
  geom_point(size=2)+
  scale_color_grey()+theme_classic()+
  geom_smooth(method=lm,linetype="dashed",color="black",fill="grey")
p+labs(x="PCA3",y="Adult TL (bp)")
```

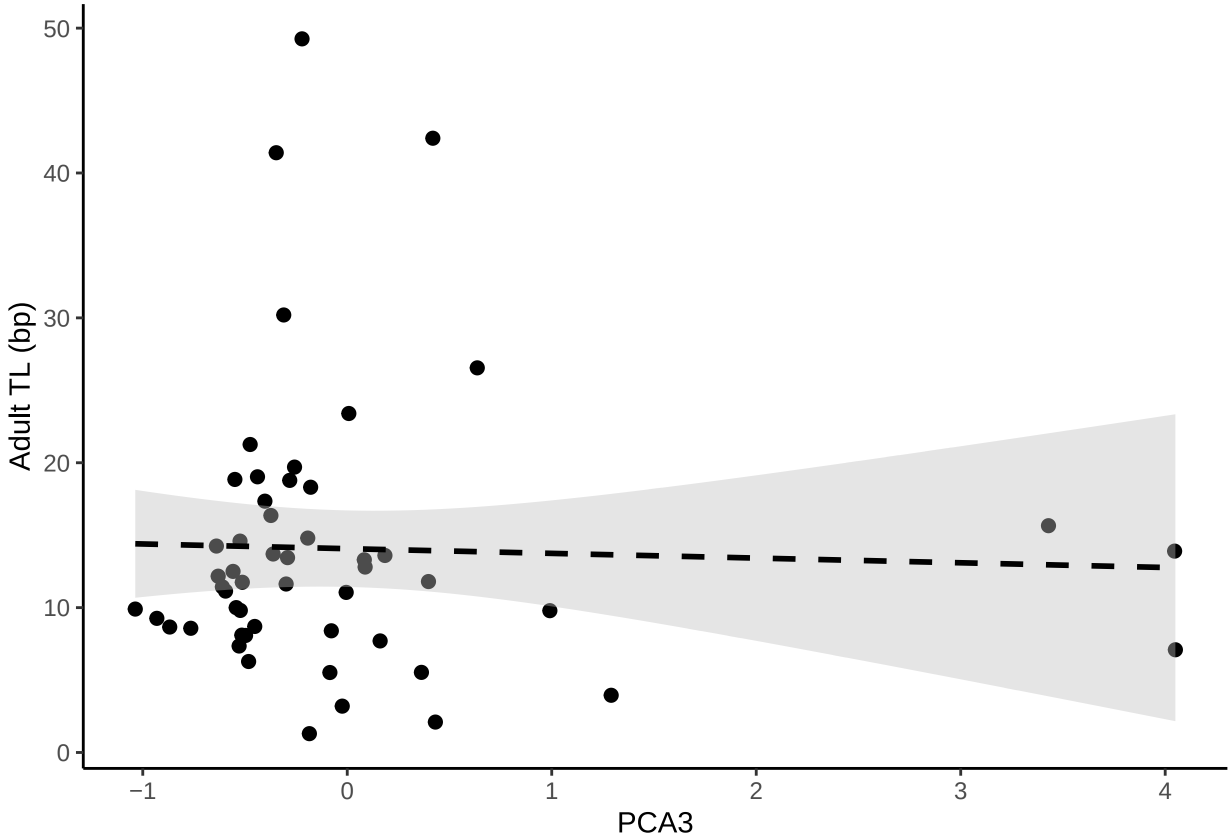

We can use the MCMCglmm function posterior.cor() to convert the posterior (co)variance components to posteriors of the correlation. To this end, we need to identify the columns holding the posteriors (varX covXY, covYX, varY). The following output illustrates the principle:

Variance and covariance explained by phylogeny (animal)

```
#colnames(m112.mcmc$VCV) #to see where the VCV matrix elements are

#MeanTL + PCA1
posterior.mode((m112.mcmc$VCV[,c('traitMean.telomere.adult:traitMean.telomere.adult.animal',"traitPCA1:t
```

```
## traitMean.telomere.adult:traitMean.telomere.adult.animal
##                                                 16.206317
##                traitPCA1:traitMean.telomere.adult.animal
##                                                 -1.740873
##                traitMean.telomere.adult:traitPCA1.animal
##                                                 -1.740873
##                               traitPCA1:traitPCA1.animal
##                                                  8.307329
```

```
HPDinterval((m112.mcmc$VCV[,c('traitMean.telomere.adult:traitMean.telomere.adult.animal',"traitPCA1:tra
```

```
##                                                                    lower      upper
```

```
## traitMean.telomere.adult:traitMean.telomere.adult.animal   4.031578 100.35637
## traitPCA1:traitMean.telomere.adult.animal                -31.987751  17.58439
## traitMean.telomere.adult:traitPCA1.animal                -31.987751  17.58439
## traitPCA1:traitPCA1.animal                                 1.925901  23.07216
## attr(,"Probability")
## [1] 0.95
```

```
#MeanTL + PCA2
posterior.mode((m112.mcmc$VCV[,c(1,3,9,11)]))
```

```
## traitMean.telomere.adult:traitMean.telomere.adult.animal
##                                                16.2063173
##                 traitPCA2:traitMean.telomere.adult.animal
##                                                -0.4444579
##                 traitMean.telomere.adult:traitPCA2.animal
##                                                -0.4444579
##                                traitPCA2:traitPCA2.animal
##                                                 0.6586806
```

```
HPDinterval((m112.mcmc$VCV[,c(1,3,9,11)]))
```

```
##                                                                lower      upper
## traitMean.telomere.adult:traitMean.telomere.adult.animal  4.0315776 100.356369
## traitPCA2:traitMean.telomere.adult.animal                -8.7364171   8.206627
## traitMean.telomere.adult:traitPCA2.animal                -8.7364171   8.206627
## traitPCA2:traitPCA2.animal                                0.2059591   3.040411
## attr(,"Probability")
## [1] 0.95
```

```
#MeanTL + PCA3
posterior.mode((m112.mcmc$VCV[,c(1,4,13,16)]))
```

```
## traitMean.telomere.adult:traitMean.telomere.adult.animal
##                                               16.20631733
##                 traitPCA3:traitMean.telomere.adult.animal
##                                               -0.08836524
##                 traitMean.telomere.adult:traitPCA3.animal
##                                               -0.08836524
##                                traitPCA3:traitPCA3.animal
##                                                0.27611395
```

```
HPDinterval((m112.mcmc$VCV[,c(1,4,13,16)]))
```

```
##                                                                 lower      upper
## traitMean.telomere.adult:traitMean.telomere.adult.animal  4.03157756 100.356369
## traitPCA3:traitMean.telomere.adult.animal                -6.57440140   5.841732
## traitMean.telomere.adult:traitPCA3.animal                -6.57440140   5.841732
## traitPCA3:traitPCA3.animal                                0.03701813   1.961709
## attr(,"Probability")
## [1] 0.95
```

Proportion of variance explained by phylogeny

```
#For telomeres
TL_phylogeny<-m112.mcmc$VCV[,'traitMean.telomere.adult:traitMean.telomere.adult.animal']/(m112.mcmc$VCV
posterior.mode(TL_phylogeny)
```

```
##      var1
## 0.1343779
```

```
median(TL_phylogeny)
```

```
## [1] 0.2112206
```

```
mean(TL_phylogeny)
```

```
## [1] 0.244221
```

```
HPDinterval(TL_phylogeny)
```

```
##           lower    upper
## var1 0.04174255 0.532838
## attr(,"Probability")
## [1] 0.95
```

```
hist(TL_phylogeny, breaks=100)
abline(v=posterior.mode((TL_phylogeny)), col="red")
abline(v=median((TL_phylogeny)), col="blue")
abline(v=mean((TL_phylogeny)), col="green")
```

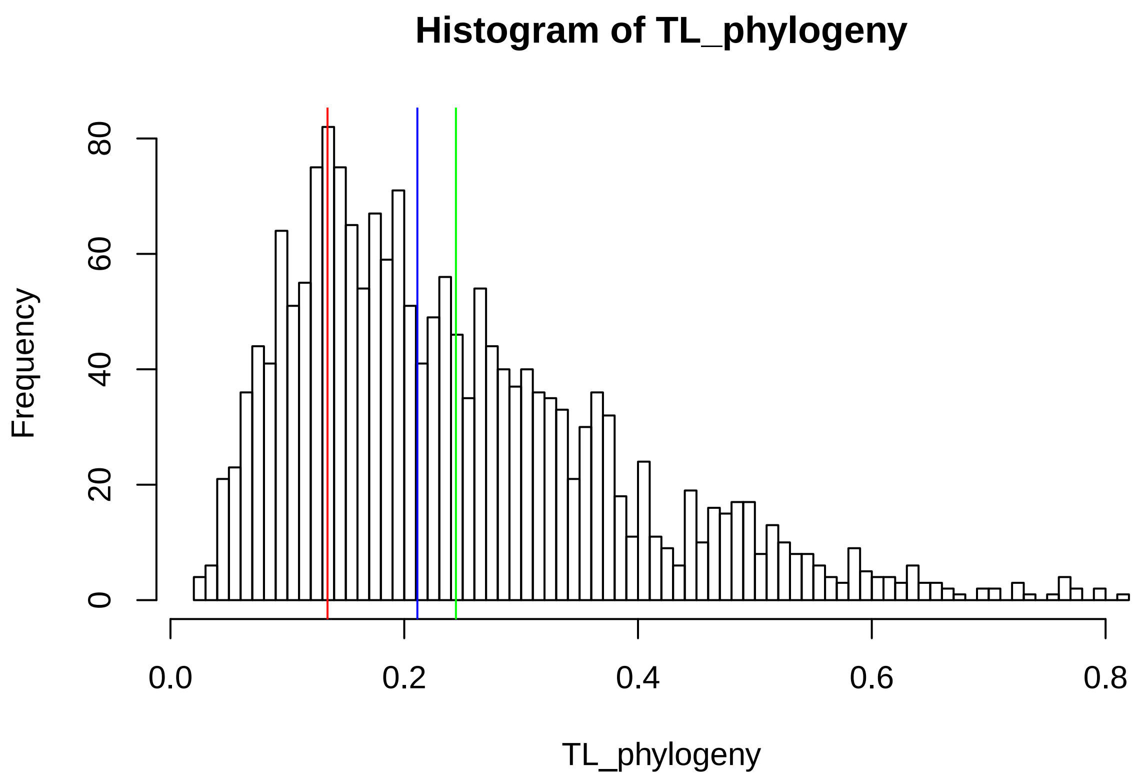


```r
#For PC1
PC1_phylogeny<-m112.mcmc$VCV[,'traitPCA1:traitPCA1.animal']/(m112.mcmc$VCV[,'traitPCA1:traitPCA1.animal

mean(PC1_phylogeny)
```

```
## [1] 0.7666461
```

```r
median(PC1_phylogeny)
```

```
## [1] 0.7957121
```

```r
posterior.mode(PC1_phylogeny)
```

```
##      var1
## 0.8683733
```

```r
HPDinterval(PC1_phylogeny)
```

```
##          lower     upper
## var1 0.4915267 0.9636186
## attr(,"Probability")
## [1] 0.95
```

```r
hist(PC1_phylogeny, breaks=15)
abline(v=posterior.mode((PC1_phylogeny)), col="red")
abline(v=median((PC1_phylogeny)), col="blue")
abline(v=mean((PC1_phylogeny)), col="green")
```

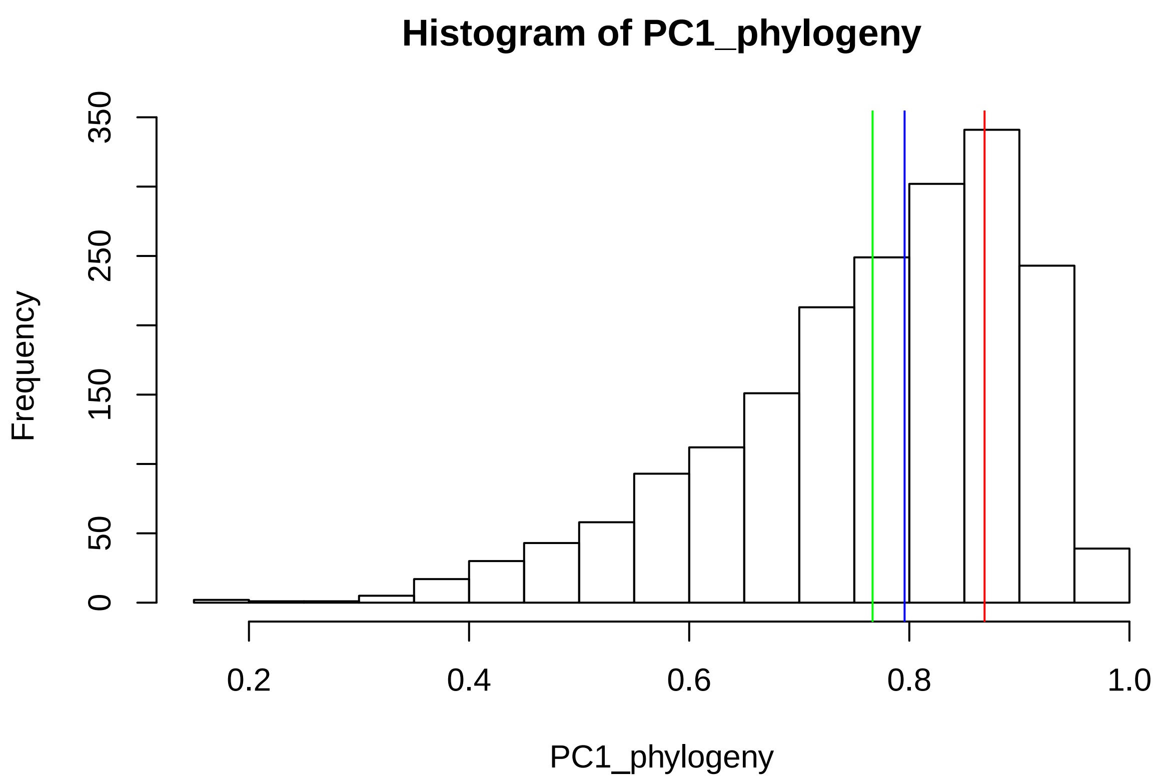


```r
#For PC2
PC2_phylogeny<-m112.mcmc$VCV[,'traitPCA2:traitPCA2.animal']/(m112.mcmc$VCV[,'traitPCA2:traitPCA2.animal

mean(PC2_phylogeny)
```

```
## [1] 0.6861445
```

```r
median(PC2_phylogeny)
```

```
## [1] 0.7080719
```

```r
posterior.mode(PC2_phylogeny)
```

```
##      var1
## 0.7945383
```

```r
HPDinterval(PC2_phylogeny)
```

```
##          lower     upper
## var1 0.3892549 0.9443612
## attr(,"Probability")
## [1] 0.95
```

```r
hist(PC2_phylogeny, breaks=15)
abline(v=posterior.mode((PC2_phylogeny)), col="red")
abline(v=median((PC2_phylogeny)), col="blue")
abline(v=mean((PC2_phylogeny)), col="green")
```

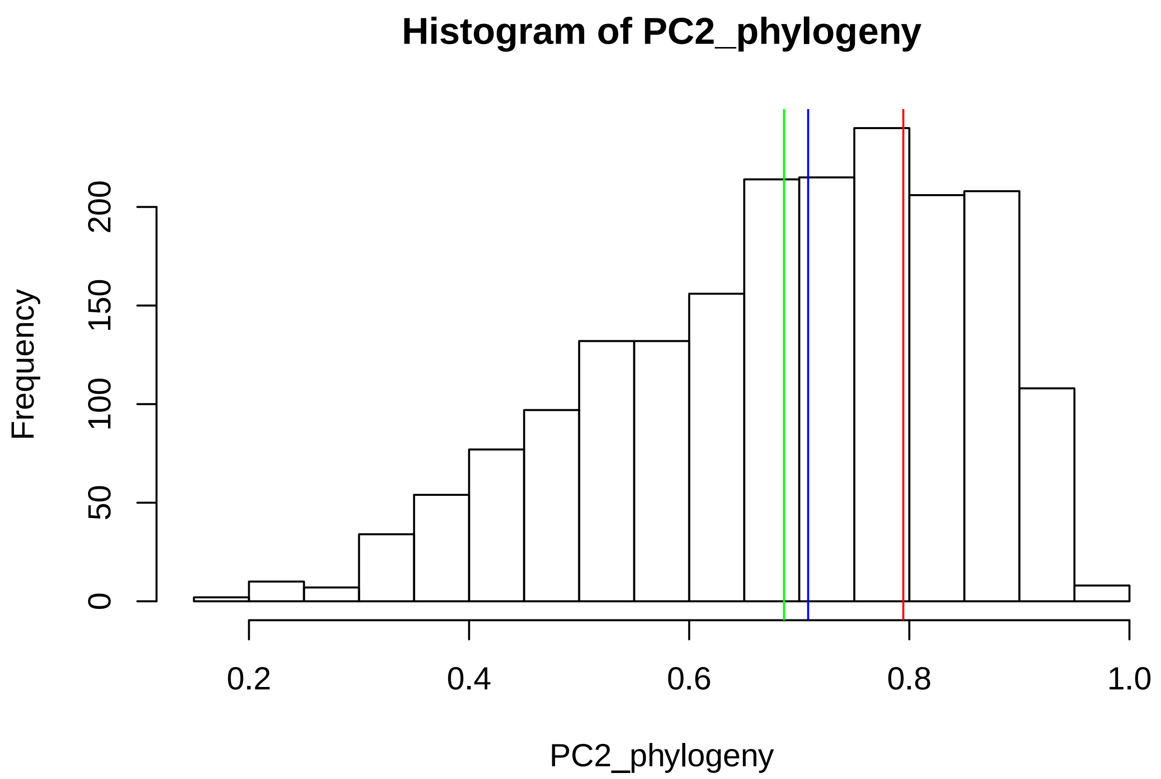


```r
#For PC3
PC3_phylogeny<-m112.mcmc$VCV[,'traitPCA3:traitPCA3.animal']/(m112.mcmc$VCV[,'traitPCA3:traitPCA3.animal

mean(PC3_phylogeny)
```

```
## [1] 0.3619078
```

```r
median(PC3_phylogeny)
```

```
## [1] 0.3250123
```

```r
posterior.mode(PC3_phylogeny)
```

```
##      var1
## 0.1923041
```

```r
HPDinterval(PC3_phylogeny)
```

```
##           lower     upper
## var1 0.04538315 0.7515136
## attr(,"Probability")
## [1] 0.95
```

```r
hist(PC3_phylogeny, breaks=15)
abline(v=posterior.mode((PC3_phylogeny)), col="red")
abline(v=median((PC3_phylogeny)), col="blue")
abline(v=mean((PC3_phylogeny)), col="green")
```

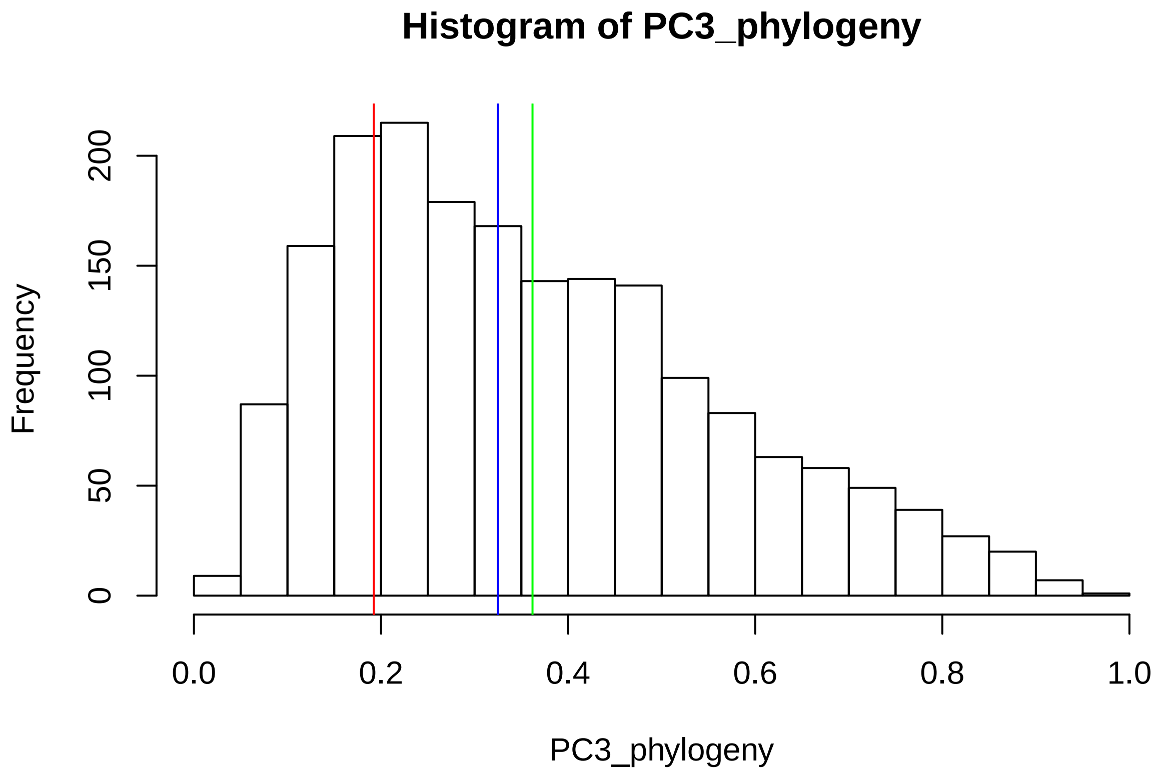

Phylogeny correlation, mode, and 95% CRI (correlation between MeanTL and PCs, explained by phylogeny)

#We can use the MCMCglmm function posterior.cor() to convert the posterior (co)variance components to posteriors of the correlation. #To this end, we need to identify the columns holding the posteriors (varX covXY, covYX, varY). #The following output illustrates the principle: # Phylogeny correlation, mode, and 95% CRI

```r
#MeanTL + PCA1
PCa1cor<-posterior.cor(m112.mcmc$VCV[,c('traitMean.telomere.adult:traitMean.telomere.adult.animal',"trai
posterior.mode(PCa1cor[,2])
```

```
##       var1
## -0.5721795
```

```r
HPDinterval(PCa1cor[,2])
```

```
##           lower     upper
## var1 -0.9451916 0.6062939
## attr(,"Probability")
## [1] 0.95
```

```r
hist(PCa1cor[,2], breaks=100)
abline(v=posterior.mode((PCa1cor[,2])), col="red")
abline(v=median((PCa1cor[,2])), col="blue")
abline(v=mean((PCa1cor[,2])), col="green")
```

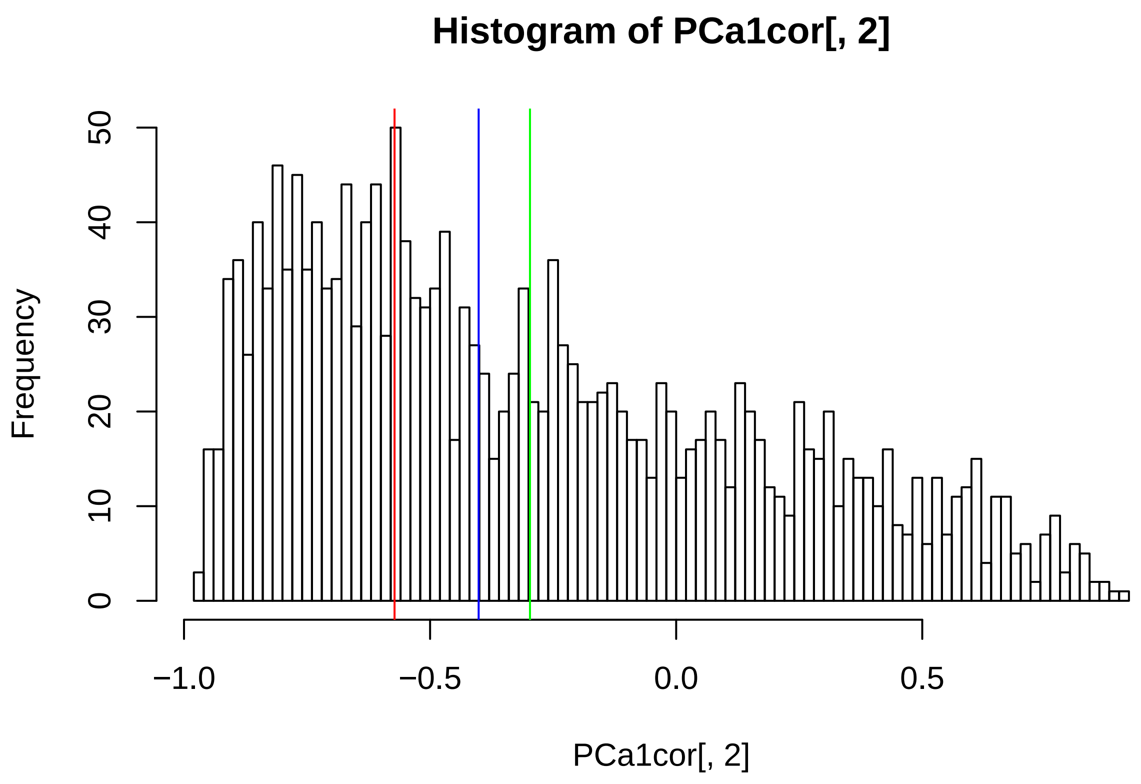


```r
#MeanTL + PCA2
PCa2cor<-posterior.cor(m112.mcmc$VCV[,c('traitMean.telomere.adult:traitMean.telomere.adult.animal',"trai
posterior.mode(PCa2cor[,2])
```

```
##        var1
## -0.0741601
```

```
HPDinterval(PCa2cor[,2])
```

```
##          lower     upper
## var1 -0.77643 0.8431683
## attr(,"Probability")
## [1] 0.95
```

```
hist(PCa2cor[,2], breaks=100)
abline(v=posterior.mode((PCa2cor[,2])), col="red")
abline(v=median((PCa2cor[,2])), col="blue")
abline(v=mean((PCa2cor[,2])), col="green")
```

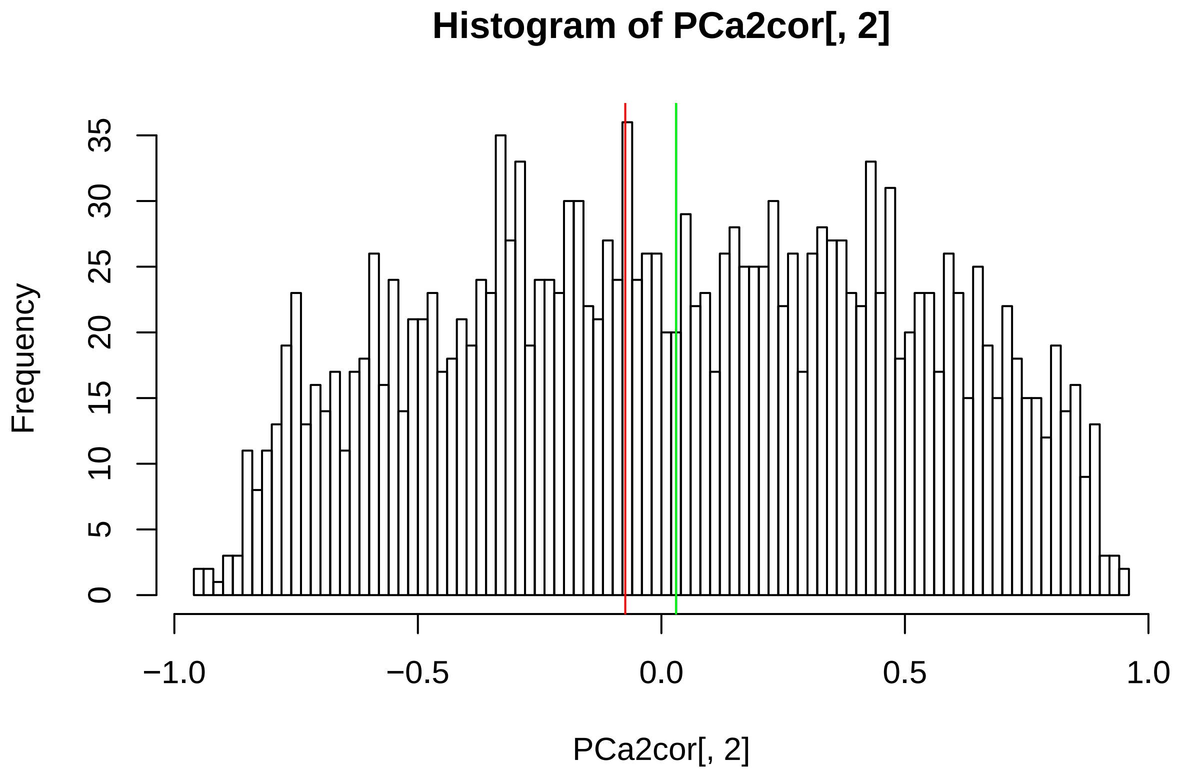


```
#MeanTL + PCA3
PCa3cor<-posterior.cor(m112.mcmc$VCV[,c('traitMean.telomere.adult:traitMean.telomere.adult.animal',"tra
posterior.mode(PCa3cor[,2])
```

```
##         var1
## -0.03712005
```

```r
HPDinterval(PCa3cor[,2])
```

```
##           lower     upper
## var1 -0.8287246 0.8014177
## attr(,"Probability")
## [1] 0.95
```

```r
hist(PCa3cor[,2], breaks=100)
abline(v=posterior.mode((PCa3cor[,2])), col="red")
abline(v=median((PCa3cor[,2])), col="blue")
abline(v=mean((PCa3cor[,2])), col="green")
```

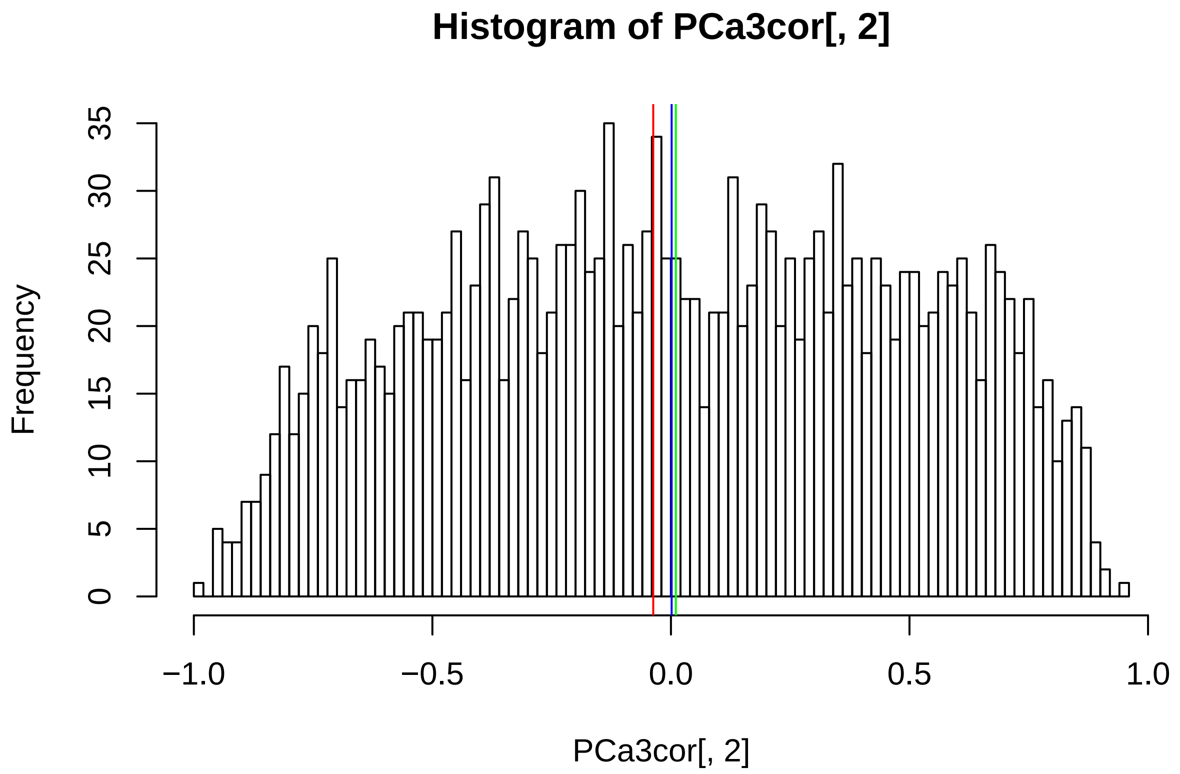

Residual correlations (Correlation between MeanTL and PCs, not explained by phylogeny), mode and 95% CRI

```r
#MeanTL + PCA1
PCa1corres<-posterior.cor(m112.mcmc$VCV[,c("traitMean.telomere.adult:traitMean.telomere.adult.units","t
posterior.mode(PCa1corres[,2])
```

```
##       var1
## -0.1919689
```

```r
HPDinterval(PCa1corres[,2])
```

```
##            lower     upper
## var1 -0.4653528 0.2488882
## attr(,"Probability")
## [1] 0.95
```

```r
hist(PCa1corres[,2], breaks=100)
abline(v=posterior.mode((PCa1corres[,2])), col="red")
abline(v=median((PCa1corres[,2])), col="blue")
abline(v=mean((PCa1corres[,2])), col="green")
```

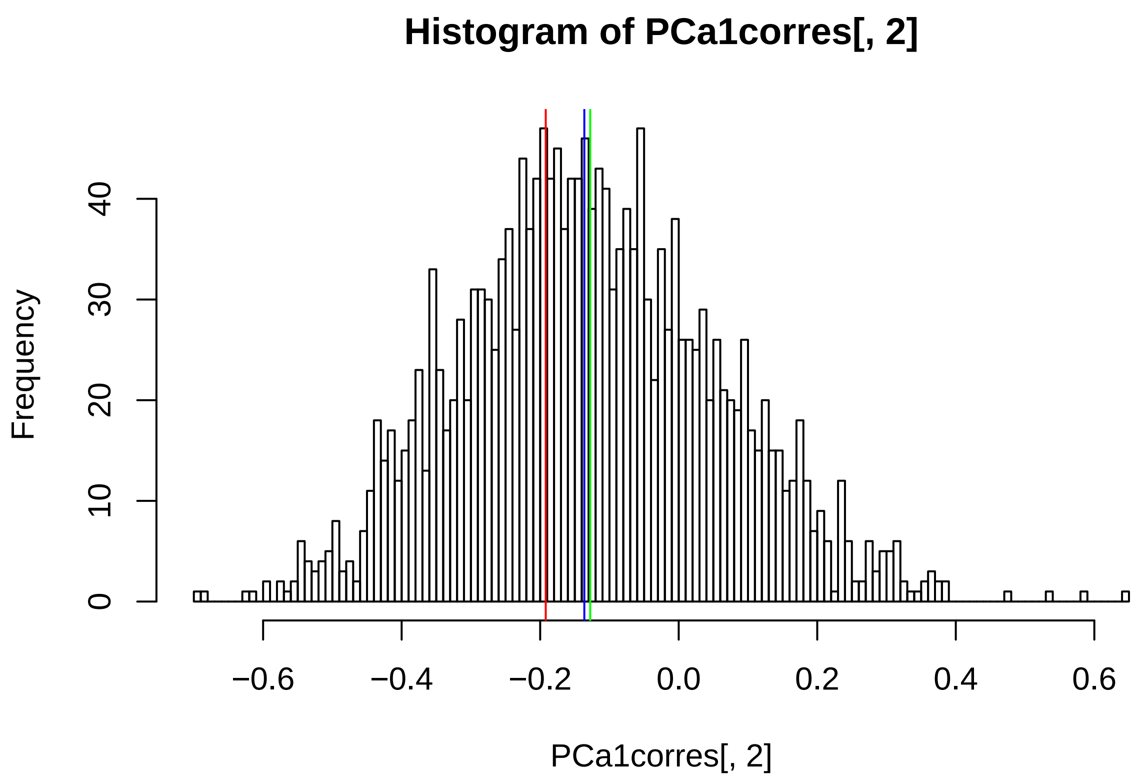


```r
#MeanTL + PCA2
PCa2corres<-posterior.cor(m112.mcmc$VCV[,c("traitMean.telomere.adult:traitMean.telomere.adult.units","t
posterior.mode(PCa2corres[,2])
```

```
##      var1
## 0.1468357
```

```r
HPDinterval(PCa2corres[,2])
```

```
##            lower     upper
## var1 -0.1596638 0.5889144
## attr(,"Probability")
## [1] 0.95
```

```r
hist(PCa2corres[,2], breaks=100)
abline(v=posterior.mode((PCa2corres[,2])), col="red")
abline(v=median((PCa2corres[,2])), col="blue")
abline(v=mean((PCa2corres[,2])), col="green")
```

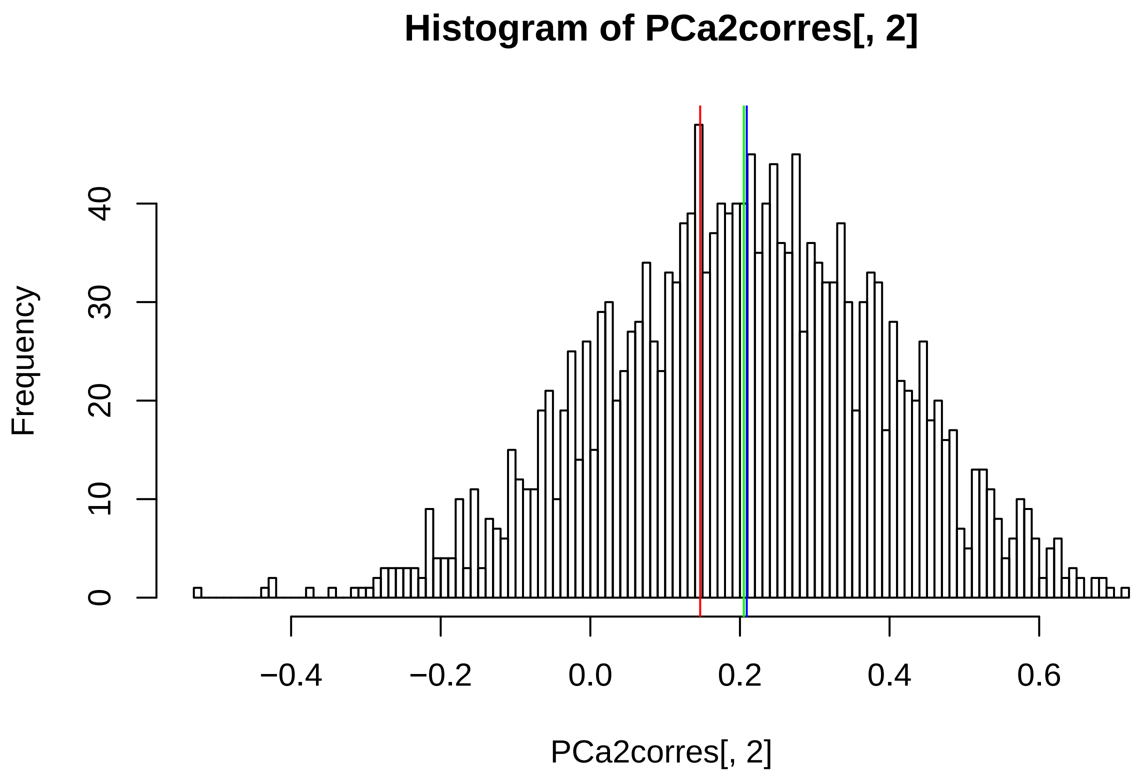


```r
#MeanTL + PCA3
PCa3corres<-posterior.cor(m112.mcmc$VCV[,c("traitMean.telomere.adult:traitMean.telomere.adult.units","t
posterior.mode(PCa3corres[,2])
```

```
##        var1
## -0.05467054
```

```r
HPDinterval(PCa3corres[,2])
```

```
##           lower    upper
## var1 -0.3966478 0.271099
## attr(,"Probability")
## [1] 0.95
```

```r
hist(PCa3corres[,2], breaks=100)
abline(v=posterior.mode((PCa3corres[,2])), col="red")
abline(v=median((PCa3corres[,2])), col="blue")
abline(v=mean((PCa3corres[,2])), col="green")
```

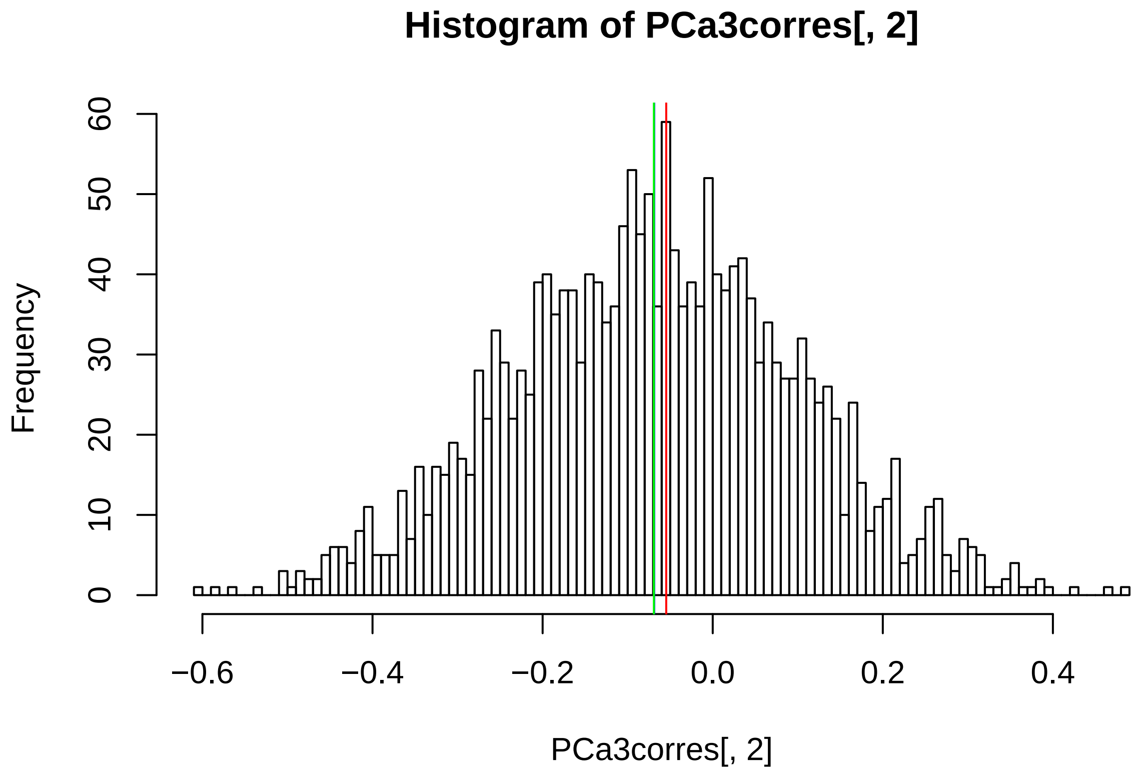
Histogram of PCa3corres[, 2]
Frequency
0
10
20
30
40
50
60
−0.6
−0.4
−0.2
0.0
0.2
0.4
PCa3corres[, 2]
